# Safety and pharmacokinetics of dolutegravir in pregnant mothers with HIV infection and their neonates: A randomised trial (DolPHIN-1 study)

**DOI:** 10.1371/journal.pmed.1002895

**Published:** 2019-09-20

**Authors:** Catriona Waitt, Catherine Orrell, Stephen Walimbwa, Yashna Singh, Kenneth Kintu, Bryony Simmons, Julian Kaboggoza, Mary Sihlangu, Julie-Anne Coombs, Thoko Malaba, Josaphat Byamugisha, Alieu Amara, Joshua Gini, Laura Else, Christie Heiburg, Eva Maria Hodel, Helen Reynolds, Ushma Mehta, Pauline Byakika-Kibwika, Andrew Hill, Landon Myer, Mohammed Lamorde, Saye Khoo

**Affiliations:** 1 Department of Molecular & Clinical Pharmacology, Institute of Translational Medicine, University of Liverpool, Liverpool, United Kingdom; 2 Infectious Disease Institute, Makerere University College of Health Sciences, Kampala, Uganda; 3 Royal Liverpool University Hospital, Liverpool, United Kingdom; 4 Desmond Tutu HIV Foundation, Cape Town, South Africa; 5 Department of Medicine, Imperial College London, London, United Kingdom; 6 Division of Epidemiology and Biostatistics and Centre for Infectious Diseases Epidemiology & Research, School of Public Health & Family Medicine, University of Cape Town, Cape Town, South Africa; 7 Department of Obstetrics and Gynaecology, Makerere University College of Health Sciences, Kampala, Uganda; Elizabeth Glaser Pediatric AIDS Foundation, UNITED STATES

## Abstract

**Background:**

The global transition to use of dolutegravir (DTG) in WHO-preferred regimens for HIV treatment is limited by lack of knowledge on use in pregnancy. Here we assessed the relationship between drug concentrations (pharmacokinetics, PK), including in breastmilk, and impact on viral suppression when initiated in the third trimester (T3).

**Methods and findings:**

In DolPHIN-1, HIV-infected treatment-naïve pregnant women (28–36 weeks of gestation, age 26 (19–42), weight 67kg (45–119), all Black African) in Uganda and South Africa were randomised 1:1 to dolutegravir (DTG) or efavirenz (EFV)-containing ART until 2 weeks post-partum (2wPP), between 9^th^ March 2017 and 16^th^ January 2018, with follow-up until six months postpartum. The primary endpoint was pharmacokinetics of DTG in women and breastfed infants; secondary endpoints included maternal and infant safety and viral suppression. Intensive pharmacokinetic sampling of DTG was undertaken at day 14 and 2wPP following administration of a medium-fat breakfast, with additional paired sampling between maternal plasma and cord blood, breastmilk and infant plasma.

No differences in median baseline maternal age, gestation (31 vs 30 weeks), weight, obstetric history, viral load (4.5 log_10_ copies/mL both arms) and CD4 count (343 vs 466 cells/mm^3^) were observed between DTG (n = 29) and EFV (n = 31) arms. Although DTG C_trough_ was below the target 324ng/mL (clinical EC90) in 9/28 (32%) mothers in the third trimester, transfer across the placenta (121% of plasma concentrations) and into breastmilk (3% of plasma concentrations), coupled with slower elimination, led to significant infant plasma exposures (3–8% of maternal exposures). Both regimens were well-tolerated with no significant differences in frequency of adverse events (two on DTG-ART, one on EFV-ART, all considered unrelated to drug). No congenital abnormalities were observed. DTG resulted in significantly faster viral suppression (P = 0.02) at the 2wPP visit, with median time to <50 copies/mL of 32 vs 72 days. Limitations related to the requirement to initiate EFV-ART prior to randomisation, and to continue DTG for only two weeks postpartum.

**Conclusion:**

Despite low plasma DTG exposures in the third trimester, transfer across the placenta and through breastfeeding was observed in this study, with persistence in infants likely due to slower metabolic clearance. HIV RNA suppression <50 copies/mL was twice as fast with DTG compared to EFV, suggesting DTG has potential to reduce risk of vertical transmission in mothers who are initiated on treatment late in pregnancy.

**Trial registration:**

clinicaltrials.gov NCT02245022

## Abstract 2

Saye Khoo and co-authors study the safety and pharmacokinetics of the antiretroviral drug dolutegravir in pregnant women initiating treatment in the third trimester.

## Author summary

**Why was this study done?:**

- In low- and middle-income settings, many women continue to present with untreated HIV in late pregnancy. Reducing maternal HIV viral load by the time of delivery is the most important single factor in preventing mother to child transmission of HIV.
- Current efavirenz-based regimens do not suppress HIV viral load sufficiently quickly when initiated in late pregnancy. In non-pregnant adults, dolutegravir-based therapy was associated with significantly faster viral suppression; however data for safety, efficacy and pharmacokinetics in pregnancy are lacking.
- We hypothesised that in women presenting in late pregnancy, dolutegravir would increase the likelihood of attaining undetectable viral load by delivery. However, it was first necessary to evaluate whether the standard dose of 50 mg once daily was sufficient in the third trimester of pregnancy, and to quantify the amount of drug to which the infant was exposed via the placenta and through breastfeeding.

**What did the researchers do and find?:**

- 60 mothers initiating therapy in third trimester were randomised to receive efavirenz-based (standard of care) or dolutegravir-based therapy.
- For those on dolutegravir, intensive pharmacokinetic sampling was undertaken at steady-state and post-partum, accompanied by paired cord and maternal blood samples at delivery, and paired maternal plasma and breast milk, and infant plasma sampling.
- We observed high in-utero transfer, and modest breastmilk transfer of dolutegravir to the infant. This, coupled with the delayed elimination of dolutegravir in newborn infants, resulted in high dolutegravir exposure in infants in-utero and in the first two weeks of life.

**What do these findings mean?:**

- Standard dolutegravir dosing appears safe and is potentially beneficial in late pregnancy.
- The high infant exposures to dolutegravir in utero, and in the first week of life, may offer additional prophylaxis against HIV transmission.
- Dolutegravir resulted in superior virological suppression compared to efavirenz among women commencing antiretroviral therapy in late pregnancy.

## Introduction

Approximately 1.5 million HIV-infected women become pregnant worldwide each year, the majority from low- or middle- income countries. In sub-Saharan Africa, a significant proportion of women are diagnosed late in pregnancy, with as much as 20% initiating anti-retroviral therapy (ART) in the third trimester in South Africa [1]. Late ART initiation in pregnancy is a major concern as it is associated with a 7-fold higher risk of mother-to-child transmission (MTCT) compared to women who initiated ART prior to 28 weeks of gestation, and a doubling of infant mortality in the first year of life [2].

In non-pregnant adults, dolutegravir (DTG) reduces HIV viral load (VL) to <50 copies/mL after a median of 28 days, compared to 84 days for efavirenz (EFV) [3] which is currently standard-of-care in many countries. Generic manufacture of DTG-containing fixed dose combinations has made this drug affordable and accessible for low income countries, and many countries are considering the place of DTG in national guidelines.

DolPHIN-1 (Dolutegravir in pregnant HIV mothers and their neonates, NCT02245022) was an open-label randomised clinical trial of DTG compared with EFV-based standard-of-care ART in women presenting with untreated HIV in the third trimester of pregnancy. The primary objective was to investigate the steady-state pharmacokinetics of DTG in HIV-infected women during the third trimester of pregnancy and after two weeks postpartum as defined by the area under the concentration-time curve of DTG between zero and 24 hours (AUC_0-24_). Secondary pharmacokinetic endpoints included cord to maternal plasma DTG ratio (C:M ratio), maternal breast milk to plasma DTG ratio (M:P ratio), and infant DTG concentrations at maternal steady state and at one, two and three days following maternal discontinuation of DTG. Secondary endpoints also included viral load in each arm at delivery and the change in viral load over the first four weeks of therapy.

## Methods

The study was conducted in accordance with the Consort Statement (Consort Checklist is available as S1 Table). Subjects were identified at the Mulago National Referral Hospital, Kampala, Uganda and Gugulethu Community Health Centre, Cape Town, South Africa with study procedures undertaken at the Infectious Diseases Institute, Makerere University College of Health Sciences, and at the Desmond Tutu HIV Foundation Clinical Trials Unit, respectively. Ethical approvals were obtained from the University of Liverpool Research Ethics Committee, the Joint Clinical Research Centre Research Ethics Committee, Uganda; and the University of Cape Town Human Research Ethics Committee, South Africa. Regulatory approvals were obtained from the Uganda National Council of Science and Technology, the Ugandan National Drug Authority and the South African Medicines Control Council. Initial recruitment occurred on 9^th^ March 2017, with final follow-up of the last participant on 6^th^ December 2018. Study protocol and statistical analysis plan are available as supplementary files, S1 Text and S2 Text.

### Sample size considerations

Previous data on 50mg once daily DTG dosing in North American HIV-infected non-pregnant healthy volunteers yielded an AUC 43,400 ng.h/mL at steady state (coefficient of variation [CV] 20%)[4]. Assuming similar variance in HIV-positive pregnant African women, recruitment of 30 subjects would yield >95% power (paired design) to show a difference of +25% difference in mean AUC (the FDA upper boundary for bioequivalence) at α = 0.05. However, data for other antiretrovirals such as the protease inhibitors suggest that variance is greater in pregnant women. It was therefore judged that recruitment of 60 pregnant women (30 on DTG) would both allow detection (at 80% power) of a DTG AUC difference of 12% (CV 20%), 16% (CV 30%), 22% (CV 40%), 27% (CV50%), so that even with doubling in CV, there would be >80% power to detect change in AUC outside the FDA bounds for bioequivalence (80–125%) and also allow meaningful exploration of differences in virological dynamics between DTG and standard of care.

### Study procedures

Between 9^th^ March 2017 and 16^th^ January 2018, potential participants were identified and screened at antenatal clinics associated with the study sites. Participants were enrolled if they were willing and able to provide informed consent; to comply with scheduled visits, treatment plans, laboratory tests and other study procedures; were aged at least 18 years; and had untreated HIV in late pregnancy, defined as greater than 28 and less than 36 weeks’ gestation by best available methods of estimation. Participants were excluded if they had received any antiretroviral drugs in the previous six months; had ever received integrase inhibitors; were anaemic (haemoglobin less than 8 g/dL); had elevations in serum levels of alanine aminotransferase (ALT) greater than 5 times the upper limit of normal (ULN) or ALT >3xULN and bilirubin >2xULN (with >35% direct bilirubin); had active hepatitis B infection; a history or clinical suspicion of unstable liver disease (as defined by the presence of ascites, encephalopathy, coagulopathy, hyperbilirubinaemia, oesophageal or gastric varices or persistent jaundice); had severe pre-eclampsia, or other pregnancy related events such as renal or liver abnormalities (grade 2 or above proteinuria, elevation in serum creatinine (>2.5 x ULN), total bilirubin, ALT or AST); or clinical depression or evidence of suicidal ideation.

HIV-infected pregnant women were randomised 1:1 to receive once daily DTG 50mg plus tenofovir disoproxil fumarate with either lamivudine or emtricitabine (DTG-ART) or standard of care (SoC) consisting of once daily EFV plus plus tenofovir disoproxil fumarate with either lamivudine or emtricitabine (EFV-ART). Due to the national policy requirements in both Uganda and South Africa for pregnant women to commence ART on the day of HIV diagnosis [5, 6], balanced against the need to screen against eligibility criteria for safety, all consenting women were commenced on EFV-based ART. Screening bloods were reviewed between three and seven days later, and at this point participants were enrolled in the study and randomised to either DTG-ART or EFV-ART. DTG was provided for the study by ViiV Healthcare.

### Randomisation procedures

Permuted block randomization in three blocks was used to ensure an even distribution of the first 16 participants across arms for a scheduled interim safety analysis, followed by a single block for the subsequent 44 participants. Randomization schedules were generated using Sealed Envelope (www.sealedenvelope.com) stratified for each site.

### Pharmacokinetic assessments

Pharmacokinetic analysis was undertaken in mother-infant pairs receiving DTG-ART, and involved sampling of i) maternal plasma (in 3^rd^ trimester and post-partum), ii) paired cord: maternal plasma, iii) paired maternal plasma:whole breastmilk, iv) plasma of breastfed infants and v) serial sampling (following DTG discontinuation) of maternal plasma and breast milk, and infant plasma. DTG was administered with a medium fat breakfast. Briefly, rich sampling (pre-dose, and at 0.5, 1, 2, 3, 4, 6, 8, 24 hours post observed dosing) was undertaken in mothers within 2 weeks after starting DTG. At delivery, where possible, a sample of paired maternal and cord blood was taken. At the post-partum visit, a further intensive pharmacokinetic assessment was performed in each mother. Additionally, breast milk (BM) was sampled pre-dose (BM_trough_) and at 2–6 hours post-dose (BM_max_). Since infants feed continuously throughout a maternal dose interval, we sought to estimate the lower bounds of infant exposure (Infant_trough_) by sampling infants at maternal trough, and the upper bounds of infant exposure (Infant_max_) by mandating a feed at maternal peak (T_max_; 2-3h post-dosing) then sampling infants 1h later. Finally, the ‘tail’ of elimination of DTG on maternal plasma and BM was characterised by serial sampling at 48, 72 and 96 hours following the last dose of DTG. In order to avoid excessive sampling, infants were randomly allocated to one of these three sampling points following discontinuation of maternal DTG.

DTG in plasma was extracted using liquid-liquid extraction and analyzed using a validated reversed phase liquid chromatography with a lower limit of quantification (LLOQ) set at 10 ng/ml and precision of 11% at the lowest QC (30ng/mL) [7]. Plasma samples were stored at -80°C before shipment to the Bioanalytical Facility, University of Liverpool for bioanalysis. Breast milk samples were spotted on Whatman 903 Protein Saver cards (GE Healthcare, Little Chalfont, Buckinghamshire, UK). The cards were air-dried at room temperature and stored with desiccant sachets and a humidity indicator in individual gas impermeable zip-lock bags at 4°C until extraction. Extraction was by liquid-liquid extraction using TBME, and dolutegravir–d5 as internal standard. Linearity was maintained from 10 ng/mL to 4000 ng/mL. Inter- and intra-day accuracy and precision were ranged from 7–11%, 3–7% and 4–6% at low, medium and high QC levels and accuracy was within ±13–14%, ±3–5% and 0–2% for low, medium and high QC levels and ±0–4% for the LLOQ [8].

### Safety assessments

Maternal safety assessments included clinical review and measurement of full blood count, urea, sodium, potassium, creatinine (and calculation of estimated glomerular filtration rate using the Cockroft-Gault formula), creatine phosphokinase, alanine aminotransferase, bilirubin and glucose. All clinical adverse events were categorised according to the Medical Dictionary for Regulatory Activities (MedRA; version 21.1), and assessed for severity according to the DAIDS Table for Grading the Severity of Adult and Pediatric Adverse Events (2004). Causality assessments were undertaken by a dedicated clinical pharmacist using the Liverpool Causality Assessment Tool [9]. Participants were reviewed for safety and tolerability after 7, 14 and 28 days on treatment, and after 56 days if delivery had not taken place. Following delivery, safety assessments were undertaken at 14 days and six months postpartum. The Edinburgh Postnatal Depression Scale was administered prenatally to establish a baseline for each participant, and at two weeks and six months postpartum [10].

Obstetric outcomes were assessed, including mode of delivery, length of rupture of membranes and any delivery complications. Neonatal assessments included surface examination for congenital anomalies, APGAR scores, gestational age at delivery (using best available estimation of gestational age, based on dates of last normal menstrual period and ultrasound measurement), neonatal length, weight and head circumference.

### HIV viral load

HIV RNA was quantitated at each site using Roche COBAS AmpliPrep/TaqMan HIV-1 Test, version 2 (lower limit of quantification of 20 copies/mL). All participants had HIV viral load performed at screening. Since all participants immediately commenced EFV-ART, but were then randomised to continue or switch to DTG-ART up to seven days later, participants allocated to DTG-ART had a repeat HIV viral load performed at switch, to evaluate the impact of prior EFV-ART. A subsequent protocol amendment allowed capture of similar data on participants randomised to EFV-ART. Viral load measurements were repeated after 14 and 28 days on treatment, and at two weeks postpartum.

Viral load results were reviewed by the Independent Data and Safety Monitoring Board (IDSMB) at two scheduled interim analyses involving the first 16 participants (8 on DTG-ART). The first of these took place after the first 8 subjects (recruited between 28-32w gestation) had delivered, the restriction of the upper limit of gestation at recruitment being applied so that there would be time to adjust her treatment regimen should an inadequate virological response be detected. Following this, a further review took place after the next 8 subjects (recruited between 28-36w gestation) had delivered. This was to ensure that the standard DTG dose of 50mg once daily did not risk exposing women to an impotent regimen in the third trimester. The following stopping criteria were predefined for these initial participants in the DTG-ART arm: at two weeks of ART, VL response of <1 log_10_ reduction or remaining ≥10 000 copies/ mL triggered assessment of adherence or factors affecting absorption and a further viral load in two weeks. At four weeks of ART, VL reduction <1 log10 or evidence of blunting of virological response between weeks two and four prompted immediate switch to an EFV-based regimen. If there was >1 log_10_ reduction, but VL remained ≥ 10 000 copies/ml, DTG was continued but HIV VL was performed two-weekly until VL <1000 copies/ml. This interim IDSMB review was completed and the study progressed to full enrolment.

### Statistical analysis

#### Pharmacokinetic data

Pharmacokinetic parameters including the DTG trough concentration (C_24_), defined as the concentration at 24 hours after the observed drug dose, the maximum observed plasma concentration (C_max_), the elimination half-life (t_1/2_), time point at Cmax (T_max_), and total drug exposure, expressed as the area under the plasma concentration–time curve from 0–24 hours after dosing (AUC_0–24_) were calculated using non-compartmental modeling techniques, to enable computation of PK parameters from the time-course of measured drug concentrations (WinNonlin^®^, Phoenix, version 6.1, Pharsight, Mountain View, CA).

Drug concentrations were summarised by descriptive analyses, including estimating the geometric mean and associated 90% confidence intervals, mean, standard deviation, median, maximum, and minimum for all quantitative variables. Within subject differences between antepartum and postpartum PK (C_24_, C_max_, AUC_0-24_) were estimated using geometric mean ratios (GMR) and associated 90% confidence intervals (90% CI). For calculation of the M:P and IP:BM ratios, only those mother-infant pairs where DTG was above LLQ were included. Results were expressed as GM and range.

DTG plasma concentrations were compared to the drug’s in-vitro protein adjusted IC90 of 64ng/mL [11], as well as an in-vivo target of 324ng/mL derived from a phase IIa dose ranging study of 10 days of DTG monotherapy[12].

#### Virologic response

Viral load over time was presented as log_10_ HIV RNA over time on ART for each individual and the median for each treatment arm. The proportion of patients with HIV RNA in the following categories were presented by timepoint: <50, 50–199, 200–999, and ≥1,000 copies/mL. The proportion of subjects in each arm with HIV RNA <50 copies/mL at two weeks postpartum were compared by Chi-squared test. Two approaches to handle missing viral load data were used: 1) missing viral load equals failure [>50 copies/mL] (M = F) in which subjects with missing data at two weeks post-partum were assessed as experiencing failure, and 2) missing viral load equals excluded (M = X) in which subjects with missing data at two weeks post-partum were excluded from the analysis. As a sensitivity analysis, the Wilcoxon rank-sum test was used to compare log_10_HIV RNA at 2 weeks post-partum between arms for patients with an evaluable result at this timepoint (M = X). Kaplan-Meier survival curves were used to estimate time to viral suppression (defined as HIV RNA <50 copies/mL) by treatment group and were compared by the log-rank test. Analyses were performed using Stata v14.2 (StataCorp, Texas, USA) and p<0.05 was used to determine significance. Virological failure was defined as <1 log_10_ reduction by week 4 of treatment or evidence of blunting of virological response between weeks 2 and 4.

## Results

**Fig 1:**
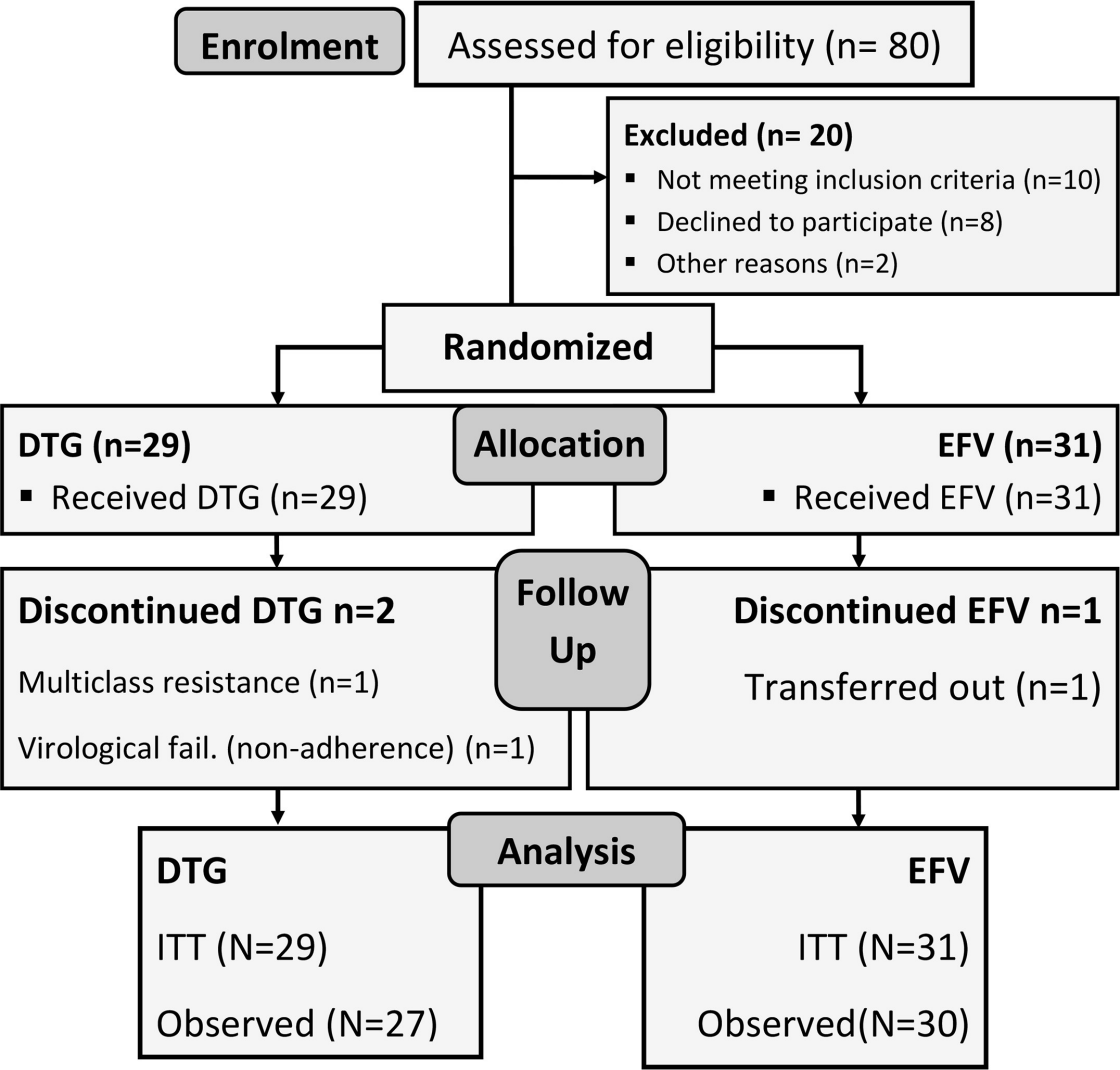
Consort diagram.

Twenty-nine participants were randomised to receive DTG-ART and 31 to EFV-ART, as illustrated in the participant flowchart in Fig 1. No differences in baseline maternal age (median 27 vs 25 years), gestation (31 vs 30 weeks), weight (65 vs 68 Kg), obstetric history, viral load (4.5 log_10_ copies/mL both arms) and CD4 count (343 vs 466 cells/mm^3^) occurred between DTG-ART and EFV-ART arms (Table 1). The median times from screening (first ART) to delivery were 60 days (IQR: 48–73) in the DTG-ART arm and 54 days (IQR: 37–77) in the EFV-ART arm. The EFV lead-in period prior to randomisation in the both arms ranged from 1 to 8 days (median: 4 days).

**Table 1:** Patient characteristics at screening.

	DTG (N = 29)	SoC (N = 31)
Age (years, median [range])	27 (19–42)	25 (19–35)
Black African (no. %)	29 (100)	31 (100)
Country of enrolment (no. %)		
South Africa	14 (52)	15 (48)
Uganda	15 (48)	16 (52)
Weight (kg, median [range])	68 (45–103)	65 (48–119)
BMI (kg/m^2^, median [range])	26 (19–40)	25 (21–46)
Estimated gestational age (weeks, median [range])	31 (27–35)	30 (27–36)
CD4 count (mm^3^, median [range])	343 (41–712)	466 (32–932)
HIV viral load (log 10 copies/ mL, median [range])	4.46 (2.43–5.14)	4.48 (2.88–6.07)
EFV lead-in time (days from screening to randomisation, median [range])	5 (1–8)	4 (1–8)
Time from first ART to delivery (days from screening, median [range])	60 (24–105)	54 (6–128)

### Pharmacokinetic data

**Fig 2:**
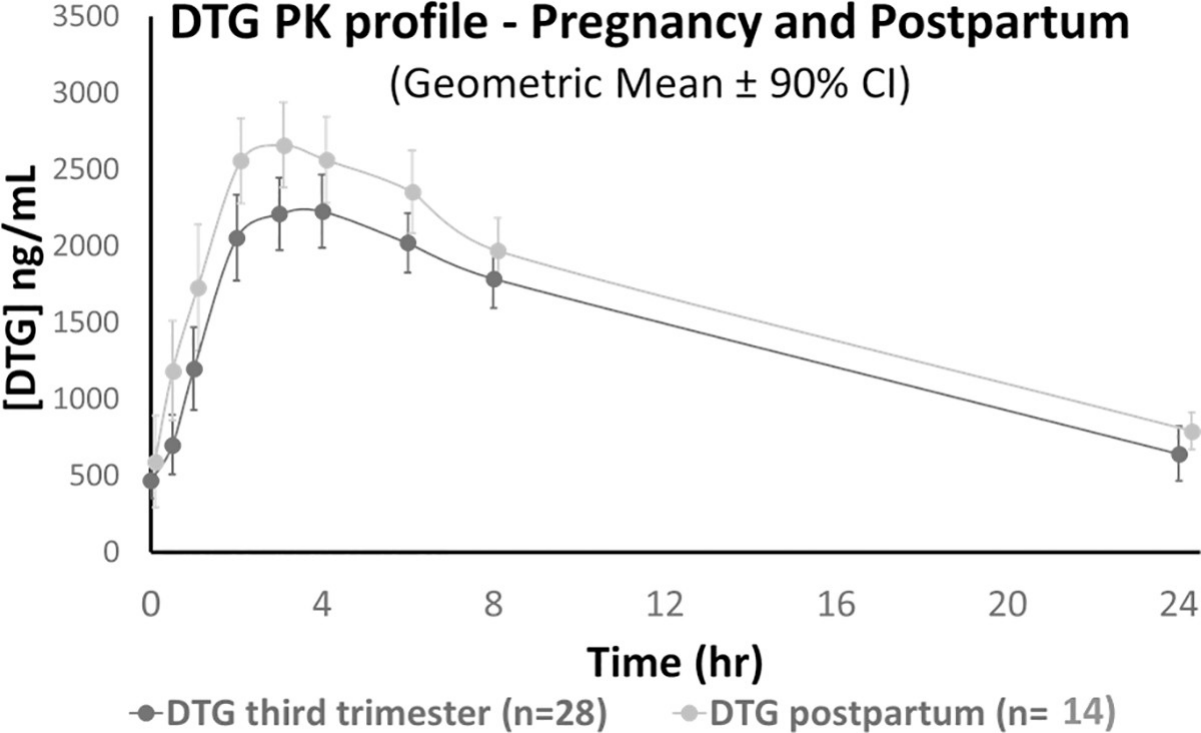
Maternal plasma DTG concentrations in 3^rd^ trimester and postpartum (median 10 days [range 7–18]).

Twenty-nine participants underwent intensive PK sampling during the third trimester. One participant had undetectable DTG concentrations in all samples, was deemed to be non-adherent to treatment (with no significant change in HIV viral load) and was excluded from analysis. Of the remaining twenty-eight participants, in third trimester, C_max_, C_24_ and AUC_0-24_ (geometric mean, range) were 2435 (1462–3986) ng/mL, 642 (188–3088) ng/mL and 35322 (19196–67922) ng.h/mL respectively. Twenty three participants underwent intensive post-partum PK sampling following delivery; the six participants who underwent sampling before seven days postpartum were excluded from analysis. The remaining 17 participants were sampled at a median of 10 (range 7–18) days following delivery, with C_max_, C_24_ and AUC_0-24_ of 2899 (1397–4224) ng/mL, 777 (348–1210) ng/mL and 40127 (22795–59633) ng.h/mL, respectively. No significant differences were observed in the geometric mean ratios of C_max_, C_24_ and AUC_0-24_ in 14 mothers who underwent sampling in the third trimester of pregnancy and at post-partum visit (Fig 2, Table 2).

**Table 2:** Maternal pharmacokinetic parameters in third trimester and two weeks postpartum and breast milk and infant plasma pharmacokinetics at median 10, range 7–18 days postdelivery.

**Maternal plasma**			
	T3 (GM, range) (n = 28)	Postpartum* (GM, range) (n = 17)	GMR (90% CI) (n = 17)
C_max_ ng/mL	2534 (1462–3986)	2899 (1397–4224)	0.83 (0.72–0.96)
C_24_ ng/mL	642 (188–3088)	777 (348–1210)	0.80 (0.63–1.02)
AUC_0-24_ ng.h/mL	35322 (19196–67922)	40127 (22795–59633)	0.85 (0.67–1.10)
**Breast Milk and Infant Plasma with Ratios to Paired Maternal Plasma**			
	GM (range) (n = 17) GM 9.8 days post	BM:MP ratio (GM, 90% CI) (n = 17)	IP:MP ratio (GM, 90% CI) (n = 17)
BM_max_ ng/mL	84.6 (43.8–171)	0.03 (0.03–0.04)	
BM_trough_ ng/mL	22.3 (3.0–64.3)	0.03 (0.02–0.04)	
Infant_max_ ng/mL	66.7 (21–654)		0.03 (0.00–0.06)
Infant_trough_ ng/mL	60.9 (16.3–479)		0.08 (0.00–0.17)

*Postpartum sampling = median (range) 10 days (7–18) post delivery

One BM and three infant samples were BLQ, and excluded from the calculation of ratios.

Paired cord and maternal blood samples were available in 16 mother-infant pairs. In one individual, both samples were below the limit of quantitation (BLQ), and non-adherence was reported. Analysis of the remaining 15 samples revealed a median C:M ratio of 1.21 (range 0.51–2.11) (Table 2).

**Fig 3:**
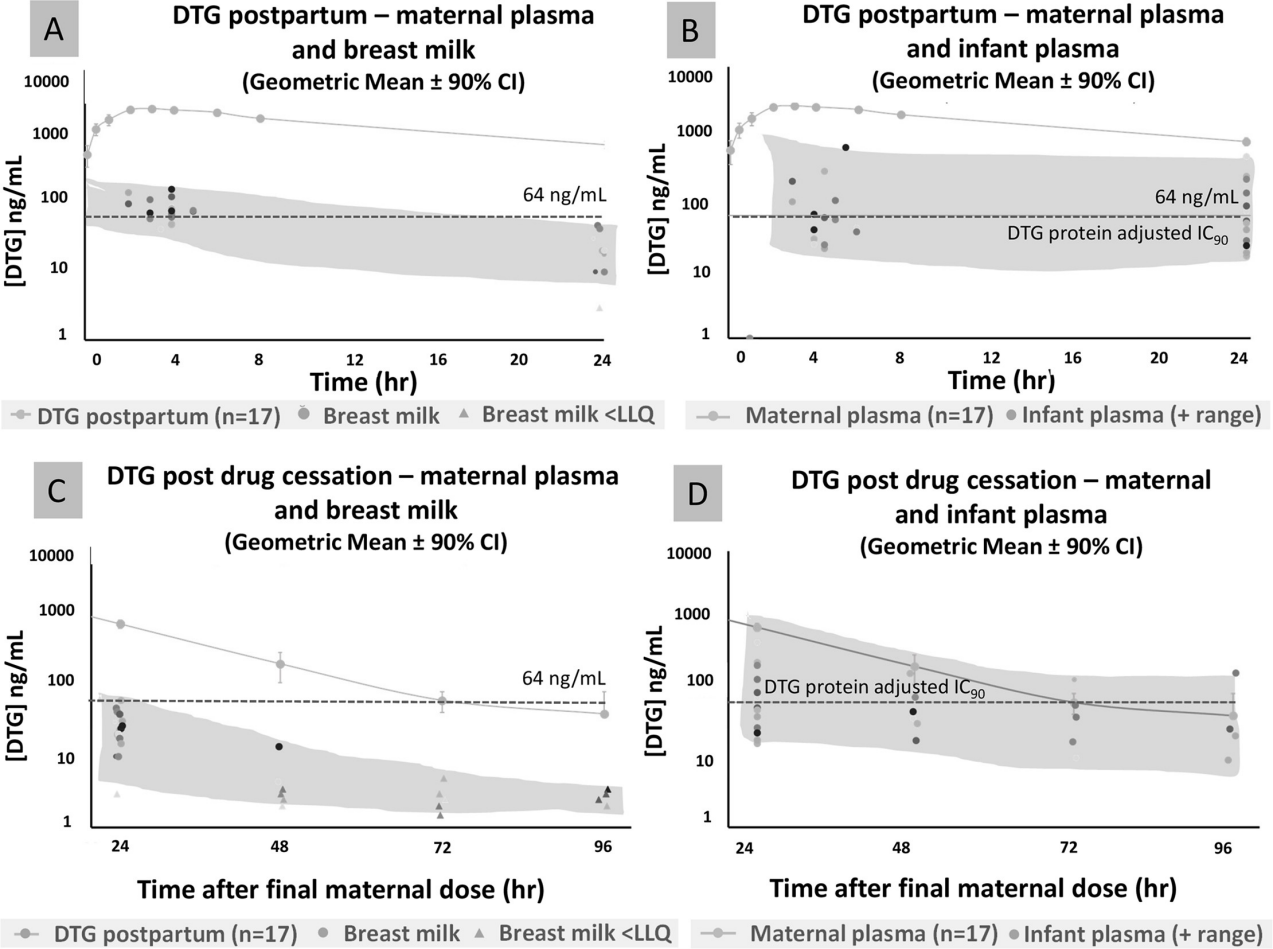
Breast milk and infant DTG concentrations at steady state and terminal elimination. A: Maternal plasma and breast milk DTG concentrations at steady-state B: Maternal plasma and infant plasma DTG concentrations at steady-state C: Maternal plasma and breast milk DTG concentrations at 24–96 hours after final maternal dose D: Maternal plasma and infant plasma DTG concentrations at 24–96 hours after final maternal dose.

DTG was detectable in breast milk with a BM_max_ of 84.6 (43.8–171) ng/mL and a BM_trough_ of 22.3 (3.0–64.3) ng/mL. A milk to plasma (M:P) ratio of 0.03 was observed throughout the dosing interval (Fig 3A, Table 2). Following discontinuation, DTG was detectable in a single BM sample at 48 hours following final maternal dose (14 ng/mL), but undetectable in all other samples at 48, 72 and 96 hours. (Fig 3C).

DTG was detectable in the plasma of breastfed infants with an Infant_max_ of 66.7 (21–654) ng/mL and an Infant_trough_ of 60.9 (16.3–479) ng/mL (Fig 3B, Table 2) at a median of 10 (range 7–18) days of age. The infant plasma to maternal plasma (IP:MP) ratios were 0.03 (0.00–0.06) at Infant_max_ and 0.08 (0.00–0.17) at Infant_trough._ After discontinuation of maternal DTG, detectable concentrations were noted in 100%, 80% and 80% breastfed infants at 48, 72 and 96 hours following final maternal dose, respectively (Fig 3D).

### Safety

Both regimens were well-tolerated in mothers, with no significant differences in adverse events between arms (see S1 and S2 Tables), with and without adjusting for initiation with EFV in the DTG-ART arm. In the DTG-ART arm, 25 (86.2%) and 4 (13.8%) mothers delivered by normal, and caesarean section, respectively; in mothers allocated to EFV-ART, the figures were 21 (67.7%) and 10 (32.3%) of pregnancies. Three mothers experienced at least one serious adverse event (SAE). Of these, 2 were in the DTG arm: i) low hemoglobin assessed as unrelated, and ii) hospitalisation due to maternal malaria and urinary tract infection associated with raised ALT, bilirubin, hypokalemia and hyponatremia. It was notable that the mother had ingested herbal medications prior to onset of the event, which was assessed as possibly related and resolved after discontinuation of DTG. This pregnancy resulted in a stillbirth that was related to a tight umbilical cord around the neck, and considered unrelated to study drug. One SAE of pre-eclampsia in a mother in the EFV-ART arm was assessed as unlikely to be related to ART.

Of 28 live births in the DTG-ART arm, median (range) gestational age at delivery was 39 (35–43) weeks, compared with 38 (34–42) weeks for the 31 live births in the EFV-ART arm, with no significant differences for gestational age or birth weight (3 [2–4] kg DTG-ART, 3 [2–4] kg EFV-ART) between study arms. No congenital anomalies were observed in the DTG-ART arm. Two infants in the EFV-ART arm had congenital malformations, one with syndactyly considered unlikely to be related to maternal study drug and one with multiple skeletal, limb and cardiac malformations (possibly TARP [Talipes equinovarus, Atrial septal defect, Robin sequence, and Persistent left superior vena cava] syndrome) considered not related to the mother’s study drug. The latter infant was born pre-term and small for gestational age, and had congenital syphilis. A third infant in the EFV-ART arm suffered from neonatal sepsis considered not related to maternal exposure and made a full recovery.

### Virologic response

**Fig 4:**
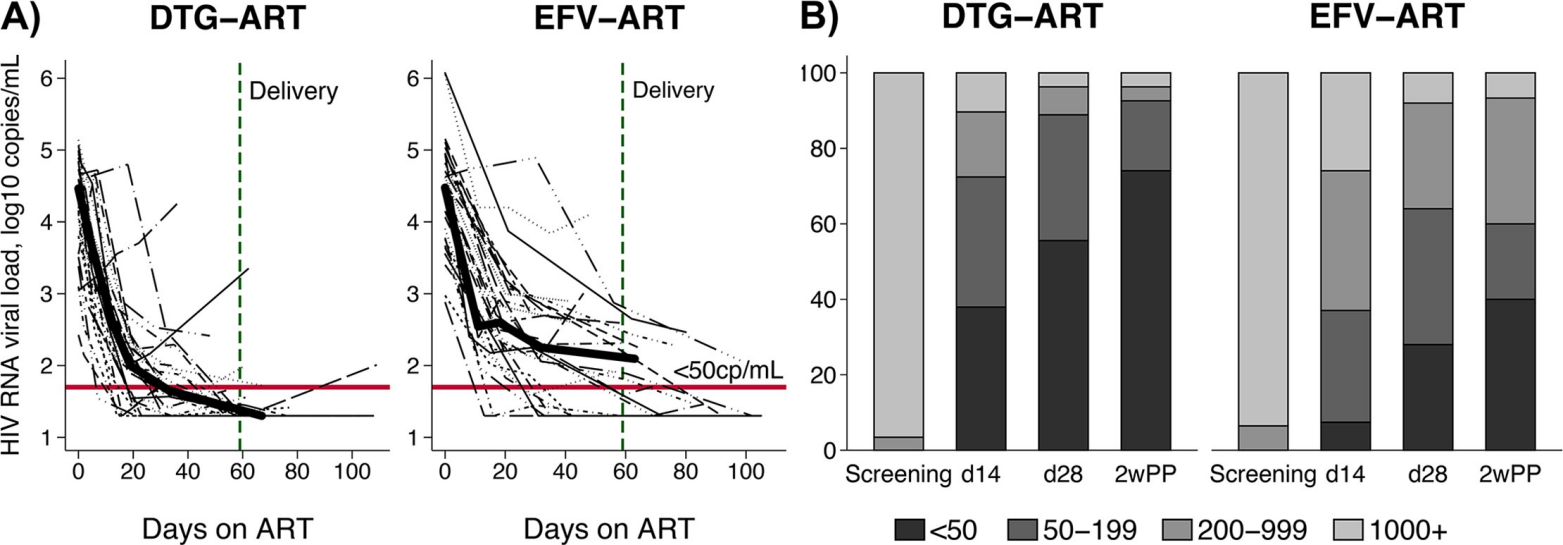
4A log_10_ HIV RNA results for each individual over time on ART. The thick line shows the median viral load at each time point (marked at the median day for each visit since screening [median days on any ART]) for each group. The vertical dashed line shows the median day of delivery (59 days) and the horizontal line shows the HIV RNA 50 copies/mL threshold. 4B Proportion of participants with HIV-RNA <50, 50–199, 200–999, and 1000+ copies/mL at each time point by arm (observed results; individuals with missing results at each timepoint were excluded [M = X]).

**Fig 5:**
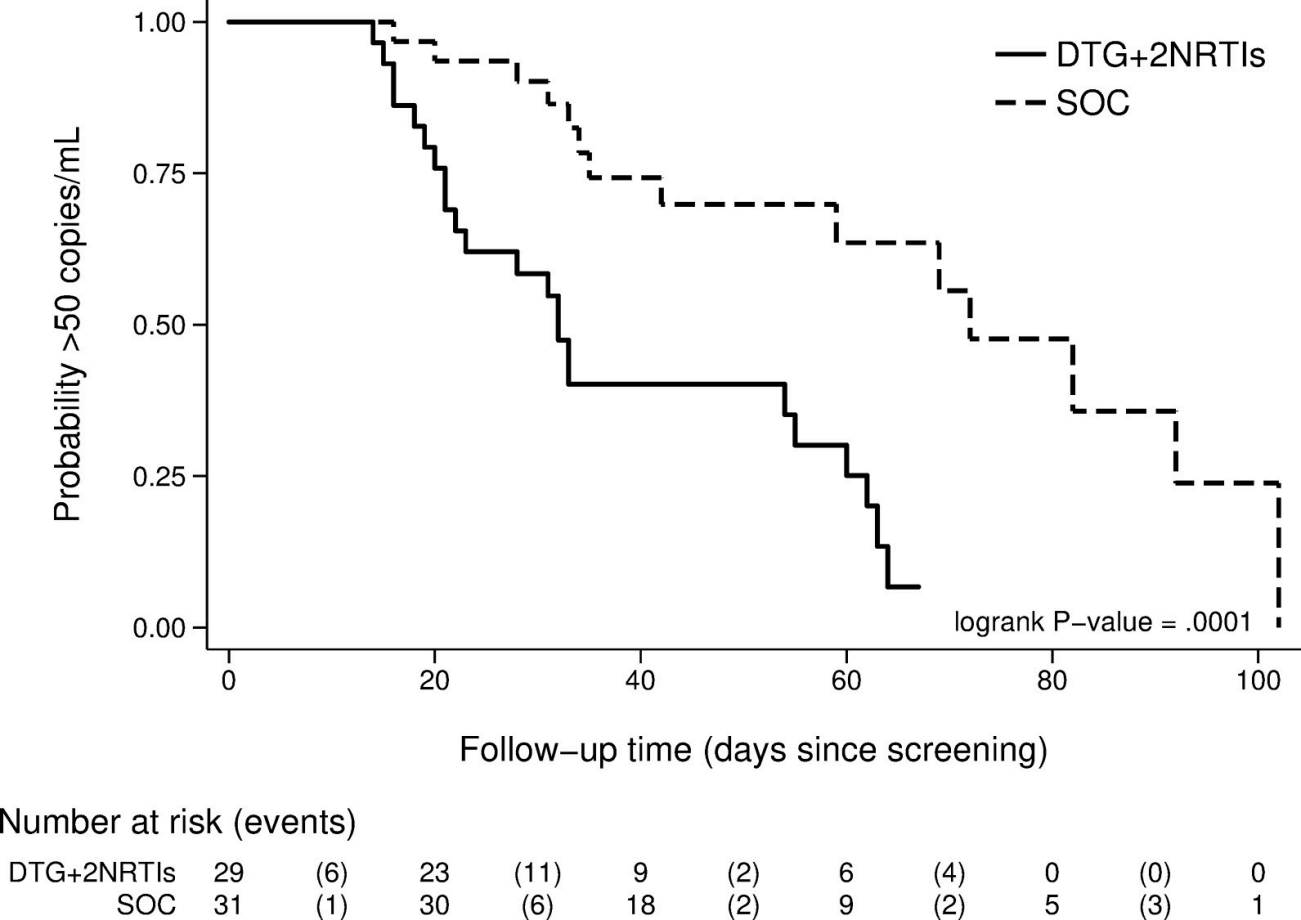
Kaplan-Meier curve indicating time to HIV viral load <50 copies/mL.

Fig 4A shows the viral load over time for each participant by group and the median viral load over time. Fig 4B shows the proportions of participants in each viral load category by study visit. At 2 weeks post-partum, the proportion with HIV RNA <50 was significantly different between arms in both the M = F (p = 0.019) and M = X (p = 0.010) analyses. Proportion undetectable was 69.0% (20/29) and 74.1% (20/27) in the DTG arm and 38.7% (12/31) and 40.0% (12/30) in the EFV-ART arm, in the M = F and M = X analyses, respectively. In analyses of log_10_ HIV RNA at 2wkPP, viral load was significantly lower in the DTG-ART arm compared with EFV-ART (p = 0.007). There was no difference in days since first ART between arms (67 IQR 55–94 for DTG-ART and 63 IQR 42–82 for EFV-ART). Fig 5 shows the probability of undetectable viral load over time, with a significant (log rank, P = 0.002) difference in time to virological suppression between the DTG-ART and EFV-ART arms.

Three patients discontinued prior to the 2-week post-partum visit (2 DTG-ART and 1 EFV-ART). One participant in the DTG-ART arm discontinued for lack of efficacy after week 4. The patient had undetectable DTG concentrations in third trimester and admitted non-adherence. Another individual in the DTG-ART arm experienced resistance and had a viral load of 2217 copies/mL at the post-partum visit. She had multi-class resistance demonstrated on her baseline sample (M41L, L201W, T215Y, M184V, Y188L, M46I, I84V, I54V, V32I, V82A, L33F, K43T) and attained virological suppression after transition to a regimen containing DTG and ritonavir-boosted darunavir. The two patients that discontinued prior to the post-partum visit for other reasons (one in each arm) both had a viral load <200 copies/mL at the point of discontinuation (4 weeks).

## Discussion

DolPHIN 1 demonstrated similar maternal DTG concentrations between the third trimester of pregnancy and postpartum, with both transplacental and transmammary exposure to the infant. Furthermore, superior virological responses were demonstrated with DTG-ART compared to EFV-ART when initiated in late pregnancy, and DTG appeared to be safe and well-tolerated in this population.

DTG exposures were lower in both the third trimester (C_24_ geometric mean 642 ng/mL) and postpartum (C_24_ geometric mean 696 ng/mL) than those previously reported in non-pregnant participants [13]. C_24_ was at or below the clinically-derived EC_90_ (324ng/mL)[12] in 9/28 (32%) and 6/27 (22%) mothers, respectively. However, it is notable that all but one DTG concentration was above the protein-adjusted IC_90_ of 64 ng/mL [12], and that with two exceptions that could be explained by complete non-adherence in one, and baseline multi-class resistance in another, all mothers responded virologically. Total exposure, defined by the AUC_0-24,_ was 35,322 ng.h/mL. This is similar to findings from the US IMPAACT P1026s study in pregnant HIV-infected women [14]. DTG is highly bound to plasma proteins, and work from the PANNA consortium has demonstrated comparable free DTG concentrations in pregnancy compared with postpartum women, related to lower serum albumin in the third trimester [15]; this supports that the impact of the moderate reduction in total (free and bound) DTG is minimal. In contrast to the IMPAACT P1026s data [14], we failed to demonstrate any significant difference in PK parameters when comparing third trimester to postpartum concentrations, except for a 17% reduction in C_max_ (P = 0.05, Table 2). This is most likely related to our early post-partum sampling when maternal physiology had not yet returned to the non-pregnant state [16].

Consistent with other studies, DTG was found to cross the placenta with a C:M ratio of 1.21 [14]. This is the first report of M:P ratio in a cohort of breastfeeding mother-infant pairs on DTG, and corroborates data from a case report [17]. This transplacental and breast milk exposure, coupled with the delayed infant clearance due to immaturity of infant UGT1A1 indicates that breastfed infants may have prolonged exposure to maternal DTG. This is seen in the prolonged elimination of DTG from infant plasma after cessation of maternal DTG: breast milk concentrations were undetectable after 24 hours, whereas 80% of infants still had measurable plasma DTG 96 hours after the final maternal dose. Population PK modelling is currently being undertaken to characterise DTG disposition in mothers, fetuses, infants and breast milk, incorporating DolPHIN-1 and other data. DolPHIN-1 evaluated a small number of infants exposed to maternal DTG, meaning two questions warrant further evaluation in larger cohorts. Firstly, whilst no drug attributable adverse events were noted in this study, scenarios of clinical concern include premature infants with further reduced UGT1A1 activity [18]. Secondly, where MTCT occurs despite maternal ART, evaluation of the impact of low dose infant exposure to DTG in selecting for resistant virus is required.

DolPHIN-1 provides confirmatory evidence that the superior virological responses observed with DTG-based combination therapy in non-pregnant adults is also seen in pregnancy. Given that differences are most marked during the first 12 weeks of therapy, DTG has a potential role in prevention of mother to child transmissions among women who are initiated on ART in the third trimester.

In women initiating ART in the third trimester of pregnancy, DTG-ART appeared safe and well-tolerated, although our sample size is small. Preliminary data from Botswana reported an increased frequency of neural tube defects among mothers who conceived on DTG [19] leading to recommendations that women of childbearing potential receiving DTG should be offered access to contraception until further data are available [20]. However, no other safety concerns have been described in women who initiate DTG later on in pregnancy [21, 22], and our results suggest a DTG-based regimen was significantly more likely to achieve a maternal HIV viral load of <50 copies/mL when compared to EFV-based ART initiated in third trimester. As maternal viral load at delivery is the principal determinant of MTCT, use of DTG-containing regimens in late-presenting pregnant women could potentially reduce the risk MTCT [23].

### Limitations

Two important limitations of the study related to the requirement to initiate immediate EFV-ART at HIV diagnosis, and the need to limit exposure of newborn and breastfed infants to what was not a recommended first-line regimen during the study period. However, randomisation ensured balance between arms in initial EFV-ART exposure, and these limitations would only have reduced any real differences between arms. The ongoing DolPHIN-2 study (NCT03249181) will provide more detailed evaluation of the safety and effectiveness of DTG in women and their infants where DTG-ART is initiated in the third trimester with follow-up until 72 weeks postpartum.

Furthermore, some women attended for postpartum visit earlier than the proposed two weeks, potentially minimising differences in DTG exposure as a result of late pregnancy. Notwithstanding, we demonstrate here that standard doses of DTG are sufficient for mothers in the third trimester of pregnancy.

### Conclusions

In summary, we found that DTG-ART resulted in a significantly shorter time to undetectable viral load, which is likely to be important in reducing MTCT when ART is initiated in late pregnancy. This is despite low steady-state exposures of DTG in the third trimester of pregnancy. We also observed significant transplacental transfer of DTG, which together with breast milk transfer, and likely delayed clearance of DTG resulted in significant DTG exposures in newborn infants.

## Supporting information

S1 Table Consort checklist. (DOC)

S2 Table Maternal and infant birth outcomes. (DOCX)

S3 Table Summary of adverse events. (DOCX)

S1 Text DolPHIN-1 protocol. (PDF)

S2 Text DolPHIN-1 statistical analysis plan. (PDF)
