# Supplementary material for: Safety and pharmacokinetics of dolutegravir in pregnant mothers with HIV infection and their neonates: A randomised trial (DolPHIN-1 study)
Source: PLoS Med. 2019 Sep 20;16(9):e1002895. doi: 10.1371/journal.pmed.1002895 (PMC6754125; doi:10.1371/journal.pmed.1002895)
Supplement: S2 Table — (DOCX) [file pmed.1002895.s002.docx]

**Supplementary Table 2 Maternal and infant birth outcomes**

|  | **DTG (n=29)** | **SoC (n=31)** |
| --- | --- | --- |
| Mode of delivery |  |  |
| Normal | 25 (86.2%) | 21 (67.7%) |
| C-section | 4 (13.8%) | 10 (32.3%) |
| Outcome of delivery |  |  |
| Normal health baby | 28 (96.6%) | 29 (93.5%) |
| Still birth | 1 (3.4%)^1^ | - |
| Congenital malformation | - | 2 (6.5%)^1,2^ |
| Gestation at birth, weeks | 39 (35-43) | 38 (34-42) |
| Length of baby, cm | 51 (44-58) | 50 (33-55) |
| Weight of baby, kg | 3 (2-4) | 3 (2-4) |
| Serious adverse event, at least one |  |  |
| Maternal | 2 (6.9%)^3^ | 1 (3.2%)^4^ |
| Infant | - | 3 (9.7%)^5^ |
| Results are number (%) or median (interquartile range) as appropriate | | |
| ^1^The still birth & both congenital malformation cases were deemed unrelated to study treatment | | |
| ^2^One case of syndactyly; one case with multiple defects: multiple skeletal and limb defects, cardiac defects, cleft palate, & hyporeflexia (likely Larsen or TARP syndrome). The infant was also pre-term & had congenital syphilis | | |
| ^3^One case of decreased haemoglobin (not related); one case with malaria + urinary tract infection (possibly related), still birth (unlikely related), & ALT + bilirubin increase + hypokalaemia + hyponatraemia (possibly related) | | |
| ^4^One case of hypertension + pre-eclampsia (unlikely related) | | |
| ^5^Includes the two cases with congenital malformations; one case of neonatal sepsis (not related) | | |
