# Supplementary material for: Safety and pharmacokinetics of dolutegravir in pregnant mothers with HIV infection and their neonates: A randomised trial (DolPHIN-1 study)
Source: PLoS Med. 2019 Sep 20;16(9):e1002895. doi: 10.1371/journal.pmed.1002895 (PMC6754125; doi:10.1371/journal.pmed.1002895)
Supplement: S3 Table — (DOCX) [file pmed.1002895.s003.docx]

**Supplementary Table 3 Summary of adverse events**

| **Adverse event** | **By randomisation arm** | | | | **As treated** | | | |
| --- | --- | --- | --- | --- | --- | --- | --- | --- |
|  | **DTG (n=29)** | | **SoC (n=31)** | | **DTG** | | **SoC** | |
|  | **All** | **≥G3** | **All** | **≥G3** | **All** | **≥G3** | **All** | **≥G3** |
| **Blood and lymphatic system** | **6** | **2** | **5** |  | **4** | **2** | **7** |  |
| Anaemia | 6 | 2 | 4 |  | 4 | 2 | 6 |  |
| Iron deficiency anaemia | 0 |  | 1 |  | 0 |  | 1 |  |
| **Cardiac** | **0** |  | **1** |  | **0** |  | **1** |  |
| Tachycardia | 0 |  | 1 |  | 0 |  | 1 |  |
| **Gastrointestinal** | **16** |  | **14** |  | **7** |  | **23** |  |
| Abdominal pain lower | 2 |  | 0 |  | 2 |  | 0 |  |
| Abdominal pain upper | 1 |  | 0 |  | 1 |  | 0 |  |
| Dental care | 1 |  | 0 |  | 1 |  | 0 |  |
| Diarrhoea | 5 |  | 2 |  | 3 |  | 4 |  |
| Dyspepsia | 2 |  | 1 |  | 1 |  | 2 |  |
| Gastritis | 1 |  | 0 |  | 1 |  | 0 |  |
| Haemorrhoids | 1 |  | 1 |  | 0 |  | 2 |  |
| Nausea | 4 |  | 5 |  | 1 |  | 8 |  |
| Toothache | 2 |  | 0 |  | 2 |  | 0 |  |
| Vomiting | 9 |  | 9 |  | 4 |  | 14 |  |
| **General disorders and administration site** | **2** |  | **3** |  | **2** |  | **3** |  |
| Asthenia | 0 |  | 1 |  | 0 |  | 1 |  |
| Chest pain | 1 |  | 0 |  | 1 |  | 0 |  |
| Fatigue | 0 |  | 2 |  | 0 |  | 2 |  |
| Pyrexia | 1 |  | 0 |  | 1 |  | 0 |  |
| **Infections and infestations** | **17** |  | **18** |  | **15** |  | **20** |  |
| Bacterial vaginosis | 0 |  | 2 |  | 0 |  | 2 |  |
| Body tinea | 1 |  | 0 |  | 1 |  | 0 |  |
| Escherichia urinary tract infection | 0 |  | 1 |  | 0 |  | 1 |  |
| Gastroenteritis | 0 |  | 1 |  | 0 |  | 1 |  |
| Hordeolum | 1 |  | 0 |  | 1 |  | 0 |  |
| Influenza | 1 |  | 1 |  | 1 |  | 1 |  |
| Lower respiratory tract infection | 2 |  | 2 |  | 2 |  | 2 |  |
| Malaria | 0 |  | 1 |  | 0 |  | 1 |  |
| Nasopharyngitis | 0 |  | 1 |  | 0 |  | 1 |  |
| Oral candidiasis | 0 |  | 1 |  | 0 |  | 1 |  |
| Pharyngitis | 0 |  | 1 |  | 0 |  | 1 |  |
| Sinusitis | 1 |  | 0 |  | 1 |  | 0 |  |
| Streptococcal urinary tract infection | 1 |  | 0 |  | 1 |  | 0 |  |
| Syphilis | 1 |  | 0 |  | 0 |  | 1 |  |
| Trichomoniasis | 1 |  | 1 |  | 0 |  | 2 |  |
| Upper respiratory tract infection | 6 |  | 3 |  | 6 |  | 3 |  |
| Urinary tract infection | 5 |  | 9 |  | 4 |  | 10 |  |
| Virologic failure | 1 |  | 0 |  | 1 |  | 0 |  |
| Vulvovaginal candidiasis | 3 |  | 2 |  | 2 |  | 3 |  |
| Wound sepsis | 0 |  | 1 |  | 0 |  | 1 |  |
| **Injury, poisoning and procedural complications** | **1** |  | **4** |  | **1** |  | **4** |  |
| Laceration | 0 |  | 1 |  | 0 |  | 1 |  |
| Procedural pain | 1 |  | 3 |  | 1 |  | 3 |  |
| **Investigations** | **3** | **1** | **2** |  | **1** | **0** | **4** | **1** |
| Blood pressure increased | 1 |  | 0 |  | 1 |  | 0 |  |
| Creatinine renal clearance decreased | 0 |  | 2 |  | 0 |  | 2 |  |
| Haemoglobin decreased | 2 | 1 | 0 |  | 0 | 0 | 2 | 1 |
| **Metabolism and nutrition** | **6** |  | **8** |  | **2** |  | **12** |  |
| Decreased appetite | 1 |  | 4 |  | 0 |  | 5 |  |
| Hypoalbuminaemia | 2 |  | 2 |  | 0 |  | 4 |  |
| Hypoglycaemia | 0 |  | 1 |  | 0 |  | 1 |  |
| Hypokalaemia | 1 |  | 0 |  | 0 |  | 1 |  |
| Hyponatraemia | 4 |  | 4 |  | 2 |  | 6 |  |
| **Musculoskeletal and connective tissue** | **1** |  | **0** |  | **1** |  | **0** |  |
| Arthralgia | 1 |  | 0 |  | 1 |  | 0 |  |
| **Nervous system** | **19** |  | **11** |  | **11** |  | **19** |  |
| Dizziness | 16 |  | 10 |  | 6 |  | 20 |  |
| Headache | 8 |  | 2 |  | 7 |  | 3 |  |
| Paraesthesia | 1 |  | 1 |  | 1 |  | 1 |  |
| Syncope | 1 |  | 0 |  | 1 |  | 0 |  |
| **Pregnancy, puerperium and perinatal conditions** | **1** |  | **2** |  | **1** |  | **2** |  |
| Gestational hypertension | 1 |  | 0 |  | 1 |  | 0 |  |
| Premature labour | 0 |  | 2 |  | 0 |  | 2 |  |
| **Psychiatric** | **3** |  | **2** |  | **1** |  | **4** |  |
| Abnormal dreams | 1 |  | 0 |  | 0 |  | 1 |  |
| Nightmare | 2 |  | 1 |  | 1 |  | 2 |  |
| Perinatal depression | 0 |  | 1 |  | 0 |  | 1 |  |
| **Renal and urinary** | **4** |  | **2** |  | **3** |  | **3** |  |
| Chronic kidney disease | 1 |  | 0 |  | 1 |  | 0 |  |
| Haematuria | 2 |  | 0 |  | 1 |  | 1 |  |
| Leukocyturia | 3 |  | 1 |  | 2 |  | 2 |  |
| Polyuria | 0 |  | 1 |  | 0 |  | 1 |  |
| Proteinuria | 3 |  | 0 |  | 2 |  | 1 |  |
| **Reproductive system and breast** | **0** |  | **1** |  | **0** |  | **1** |  |
| Vulvovaginal pruritus | 0 |  | 1 |  | 0 |  | 1 |  |
| **Respiratory, thoracic and mediastinal** | **5** |  | **4** |  | **5** |  | **4** |  |
| Cough | 3 |  | 4 |  | 3 |  | 4 |  |
| Hiccups | 1 |  | 0 |  | 1 |  | 0 |  |
| Oropharyngeal pain | 1 |  | 0 |  | 1 |  | 0 |  |
| **Skin and subcutaneous tissue** | **4** |  | **1** |  | **2** |  | **3** |  |
| Pruritus | 2 |  | 0 |  | 1 |  | 1 |  |
| Rash | 0 |  | 1 |  | 0 |  | 1 |  |
| Rash papular | 1 |  | 0 |  | 1 |  | 0 |  |
| Urticaria | 2 |  | 0 |  | 1 |  | 1 |  |
| Abbreviations: DTG, dolutegravir arm; SoC, standard of care arm  Shows number subjects experiencing at least one adverse event per row from the screening visit. Rows in bold are System Organ Class and subitems are Preferred Term. Sum of Preferred Terms do not always equal System Organ Class total as some individuals experienced more than one event within each class. *All* includes any adverse event, & *≥G3*, includes any adverse event considered grade 3 or higher. Individuals were initiated on SoC at screening, prior to randomisation, & as such *"As treated"* classifies any adverse events occurring prior to randomisation in the DTG arm as SoC | | | | | | | | |
