# Supplementary material for: Safety and pharmacokinetics of dolutegravir in pregnant mothers with HIV infection and their neonates: A randomised trial (DolPHIN-1 study)
Source: PLoS Med. 2019 Sep 20;16(9):e1002895. doi: 10.1371/journal.pmed.1002895 (PMC6754125; doi:10.1371/journal.pmed.1002895)
Supplement: S1 Text — (PDF) [file pmed.1002895.s004.pdf]

Safety and Pharmacokinetics of Dolutegravir in Pregnant HIV Mothers and their Neonates: A Pilot Study

DolPHIN I – Dolutegravir in Pregnant HIV mothers and Neonates

**SHORT TITLE:**	**DolPHIN 1Study**
**PROTOCOL NUMBER:**	PK-12
**EudraCT NUMBER**	
**NCT:**	
**VERSION NUMBER:**	21
**SPONSORS:**	University of Liverpool
**FUNDING:**	ViiV Limited
**AREA**	HIV
**Type**	Phase II

GCP Compliance Statement

This trial will be conducted in compliance with the protocol, with the principles of ICH Guideline E6 for Good Clinical Practice, the Declaration of Helsinki, and all applicable regulatory requirements.

CHIEF INVESTIGATOR/PROTOCOL CHAIR
Professor Saye KhooMD, FRCP
University of Liverpool
70 Pembroke Place
Liverpool L693GF, United Kingdom

EMAIL and TELEPHONE
Phone: +441517945560
Email: khoo@liverpool.ac.uk

INVESTIGATORS :

Principal Investigator
Mohammed Lamorde, MRCP, PhD
Head, Clinical Trials Unit
Infectious Diseases Institute,
Makerere University College of Health Sciences (MakCHS), Kampala, Uganda
Phone: +256414307291
E-mail: mlamorde@idi.co.ug

Principal Investigator
Professor Catherine Orrell
J52 Old Main Building, Groote Schuur Hospital
Main Road, Observatory
Cape Town, 7925
Phone: +27216506958
E-mail: catherine.orrell@hiv-research.org.za

Principal Investigator
Professor Landon Myer
Gugulethu Community Health Centre, Corner NY1 and NY3, Gugulethu, Cape Town
Phone: +2721406 6661
E-mail: landon.myer@uct.ac.za

ABBREVIATIONS

ACTG	AIDS clinical trial group
AE	Adverse event
ALT	Alanine transaminase
AST	Aspartate transaminase
ARV	Antiretroviral
AUC	Area under the curve
bd	Twice daily
C_{12}	Concentration at 12 h
CI	Confidence interval
C_{MAX}	Maximum concentration
C_{MIN}	Minimum concentration
CRF	Case report form
CV	Coefficient of variation
CYP	Cytochrome P450
IDSMB	Independent Data & Safety Monitoring Board
DTG	Dolutegravir
EFV	Efavirenz (an NNRTI)
FDA	U.S. Food and Drug Administration
GCP	Good clinical practice
GFR	Glomerular filtration rate
HIV	Human immunodeficiency virus
INSTI	Integrase strand transfer inhibitor
IP	Investigational product
LFTs	Liver function tests
NNRTI	Non-nucleoside reverse transcriptase inhibitor
NOAEL	No observed adverse effect level
NVP	Nevirapine (an NNRTI)
NRTI	Nucleoside reverse transcriptase inhibitor
PD	Pharmacodynamic
PK	Pharmacokinetics
PMTCT	Prevention of mother-to-child transmission of HIV
qd	Once-daily
RCT	Randomized controlled trial
SAE	Serious adverse event
SSA	Sub-Saharan Africa
SUSAR	Suspected unexpected adverse reaction
TSC	Trial Steering Committee
UGT	Uridinediphosphate glucuronosyl transferase
ULN	Upper limit of normal
VL	HIV viral load
WHO	World Health Organization

1 Contents

2 PROTOCOL SYNOPSIS 6
3 LAY SUMMARY 7
4 ETHICAL JUSTIFICATION, AND RISK-BENEFIT ANALYSIS 7
4.1 Ethical Justification 8
4.2 Risk Assessment for Dolutegravir in Pregnancy 10
4.3 Risk Management Plan 14
5 DESIGN, OBJECTIVES AND ENDPOINTS 17
5.1 Study Design 17
5.2 Primary Objectives 17
5.3 Secondary Objectives 17
5.4 Primary Endpoint 17
5.5 Secondary Endpoints 17
6 SUBJECT POPULATION 18
6.1 Number of subjects 18
6.2 Inclusion criteria 18
6.3 Exclusion criteria 19
6.4 Selection of Participants 20
6.5 Withdrawal of subjects 20
7 STUDY TREATMENTS 20
7.1 Treatment Plans and Regimens 20
7.2 Dolutegravir 21
7.3 Prior and Concomitant Medication 21
7.4 Infant Nevirapine 22
8 STUDY SCHEDULES AND PROCEDURES 23
8.1 Study Schedule 24
8.2 Screening 25
8.3 Randomisation 25
8.3.1 Drug dispensing visit, Day 1 26
8.3.2 Visit 2, Day 7 (tolerance +/- 1 day) 26
8.3.3 Visit 3, Day 14 (tolerance +/- 2 days) 26
8.3.4 Visit 4, Day 28 (tolerance +/- 7 days) 27

8.3.5 Visit 5, Delivery ... 27
8.3.6 Visit 6, 2 weeks post-delivery (tolerance +/- 7 days) ... 27
8.3.7 Visit 7, 1, 2 or 3 days following switch to Standard of Care (SoC) ... 28
8.3.8 Visit 8, month 6 post-delivery (tolerance +/- 1 month) ... 28
8.4 Clinical Assessment/Early Termination Visit ... 28
9 SAFETY MONITORING ... 28
9.1 Intensive Virological Monitoring of First 8 Patients receiving DTG ... 29
9.2 Maternal Assessments ... 29
9.3 Infant Assessment ... 30
10 MANAGEMENT OF ADVERSE EVENTS ... 31
10.1 Adverse Events ... 31
10.2 Serious Adverse Events ... 32
10.3 Specific Toxicities/Adverse Event Management ... 33
10.3.1 Liver chemistry stopping and follow up criteria ... 33
10.3.2 Allergic Reaction ... 35
10.3.3 Rash ... 35
10.4 Safety Reporting ... 36
10.4.1 Pregnancy Reporting ... 36
10.5 Adverse Event Reporting ... 37
10.6 Management of Post-recruitment Illness ... 37
11 DATA AND STATISTICAL ANALYSIS ... 37
12 STUDY MANAGEMENT ... 38
12.1 Liverpool ... 38
12.2 Sample Processing ... 38
12.3 Clinical Monitoring ... 38
12.4 Laboratory Monitoring ... 38
12.5 Data Safety and Monitoring Group ... 38
12.6 Termination of the trial ... 39
13 DATA HANDLING ... 39
13.1 Recording of Data ... 39
13.2 Source Documentation and Study Records ... 39
13.3 Data management ... 39
13.4 Archiving and storage of data ... 40

14 QUALITY CONTROL AND QUALITY ASSURANCE 40
14.1 Monitoring Arrangements 40
14.2 Quality Assurance 40
15 ETHICS & ADMINISTRATION 40
15.1 Ethics Approval 40
15.2 Regulatory Approval 41
15.3 Publication Policy 41
15.4 Drug Accountability 41
15.5 Financial Aspects 41
16 REFERENCES 41
17 PROTOCOL SIGNATURE PAGE 43
18 APPENDIX 1 SAFETY QUESTIONNAIRE 44
19 APPENDIX 2 DAIDS GRADING SCALE 46
20 APPENDIX 3 LIVERPOOL CAUSALITY ASSESSMENT TOOL FOR ADVERSE DRUG REACTIONS 65
21 APPENDIX 4 COMMUNITY CONSULTATION 66
22 APPENDIX 5 EDINBURGH POSTNATAL DEPRESSION SCALE 72

2 PROTOCOL SYNOPSIS

**Aim**	To evaluate dolutegravir (DTG) pharmacokinetics in pregnant HIV-infected women
**Rationale**	In developing countries many women present with a new HIV diagnosis in late pregnancy, and are at high risk of transmitting infection during delivery. Moreover, women may acquire NNRTI resistance from primary transmission, or use of nevirapine (NVP) in previous pregnancies. In these circumstances, DTG is likely to be more effective in reducing mother to child transmission of HIV than NNRTI-based regimens.
**Study design**	HIV positive pregnant women presenting with untreated HIV infection in late (≥28 -36 weeks gestation) pregnancy will be randomised 1:1 to receive DTG (50mg once daily) or standard of care (nevirapine or efavirenz) + 2 NRTIs. PK (0-24h) profile will be sampled in third trimester and post-partum. Although this is primarily a PK study (and has been powered as such) randomisation is included to allow comparison of plasma HIV VL responses against standard of care (NVP or EFV) and is essential for evaluation of secondary endpoints of safety and efficacy of DTG in pregnancy.
**Inclusions**	• Able to provide informed consent<br>• Willing to participate,<br>• Women age 18 years and above<br>• Pregnant<br>• Untreated HIV infection in late pregnancy at ≥28 – 36 weeks gestation
**Exclusions**	• Received antiretroviral drugs in previous 6 months<br>• Ever received integrase inhibitors<br>• Serum haemoglobin < 8.0 g/dl<br>• Elevations in serum levels of alanine aminotransferase (ALT) >5 times the upper limit of normal (ULN) or ALT >3xULN and bilirubin >2xULN (with >35% direct bilirubin)<br>• eGFR < 50ml/min<br>• Active Hepatitis B infection, history or clinical suspicion of unstable liver disease, or subjects with severe liver disease.<br>• Severe pre-eclampsia (e.g. HELLP), or other pregnancy related events such as renal or liver abnormalities (e.g. grade 2 or above proteinuria,

elevation in serum creatinine (above 2.5 x ULN), total bilirubin ALT or AST)
- Paternal non-consent (where disclosure to male partner has been made)
- Clinical depression or clinical judgement suggests increased risk of suicidality

**Primary Endpoints**	• $AUC_{0\text{-}24}$h of DTG in third trimester and post-partum
**Secondary Endpoints**	• Safety and tolerability of DTG<br>• Proportion with undetectable VL at delivery<br>Change in viral load over the first 4 weeks of therapy<br>• Plasma protein bound/unbound PK<br>• Cord:maternal plasma DTG ratio<br>• Breast milk:maternal plasma DTG ratio<br>• DTG levels in the breastfed infant with mother at steady-state , and time for DTG to become undetectable in infant blood<br>• Proportion of mother to child transmission of HIV at six months postpartum
**Number recruited**	N=30 per group

3 LAY SUMMARY

Antiretroviral therapy (ART) in pregnancy is able to effectively reduce mother-to-child transmission (MTCT) of HIV. If untreated, the risk of transmission is around 25% (greater with high viral loads) but ART administered optimally during pregnancy may reduce this risk to 1-2%. In order to successfully prevent infection, ART should be started in the first or second trimester, and should reduce maternal plasma viral load to undetectable levels. Unfortunately throughout low-middle income countries, MTCT rates are unacceptably high with an estimated 240 000 newborn children infected annually. The main causes for this are undiagnosed or late diagnosis of maternal infection, suboptimal adherence to therapy and drug resistance, particularly in mothers who have previously received single dose nevirapine.

In sub-Saharan Africa (SSA), women frequently engage with health services late in pregnancy, and new HIV diagnoses in the third trimester (≥28 weeks of pregnancy) are not uncommon. Risk of MTCT is high, especially as NNRTI-based therapy takes a median of 2 months to significantly reduce the HIV viral load, making it unlikely that commencement of these drugs in late pregnancy will offer protection of the infant from intrapartum transmission.

4 ETHICAL JUSTIFICATION, AND RISK-BENEFIT ANALYSIS

Universal HIV testing of pregnant women in low/middle income countries continues to detect new HIV diagnoses, and many of these mothers present late in pregnancy (≥28 weeks gestation). First-line use of nevirapine (NVP) in this setting with limited virological monitoring, coupled

with widespread administration of single-dose NVP to prevent mother-to-child transmission (PMTCT) has increased the prevalence of NVP drug resistance in these communities. The pattern of resistance mutations frequently confers resistance to efavirenz (EFV), the alternative NNRTI used in these populations.

The combination of late presentation and significant risk of NNRTI resistance makes dolutegravir (DTG), an Integrase Strand Transfer Inhibitor (INSTI) an ideal choice in these women, providing safety and efficacy is established- this study seeks to evaluate the PK of DTG in pregnancy, and to evaluate VL responses, safety and tolerability. Should DTG prove safe and without major pharmacokinetic alterations resulting from pregnancy, these data may inform the design of a subsequent efficacy study.

4.1 Ethical Justification

Vertical transmission of HIV remains a significant challenge in developing countries and antiretroviral prophylaxis for PMTCT is an important tool towards elimination of paediatric infections. Between 2009 and 2010, coverage of antiretroviral prophylaxis for prevention of mother to child transmission was 42% and an estimated 1.48 million infants were born to women living with HIV (WHO 2010). Global efforts are geared towards improving access to antiretrovirals for PMTCT by simplifying antiretroviral treatment protocols while ensuring optimal outcomes for HIV-infected women and their children (WHO 2010; WHO 2012).

Under consolidated antiretroviral guidelines issued by the WHO in 2013, efavirenz-based ART is now recommended the preferred NNRTI option for HIV-1 infected patients, including among women of childbearing age and pregnant women(WHO 2013). Uganda adopted the Option B+ strategy for PMTCT of HIV in April 2013, and South Africa in July 2014. Under this strategy, *lifelong* ART is offered to all pregnant ART naïve women irrespective of CD4 count with efavirenz-based ART as the preferred treatment option. However, the effectiveness of this regimen could be compromised in the event of large populations of women who may have been either exposed to single dose nevirapine in the past or among women with transmitted NNRTI resistance.

The justification for studying DTG in pregnancy includes:

a) **Likely widespread availability of generic DTG** in the coming years. The manufacturer has indicated its willingness to make DTG available in low income countries, and is currently engaging with generic manufacturers. Estimates from the Clinton Foundation suggest that generic manufacture of DTG will make this drug affordable either as an alternative first line, or else second line agent (Hill 2013). Clinical guidelines from the US and Europe currently rank INSTIs such as raltegravir and elvitegravir alongside NNRTIs as preferred first line agents. INSTIs may replace NNRTIs in first line regimens due to their good safety and toxicity profile, lower propensity for drug interactions, and superior efficacy. Large phase III RCTs comparing raltegravir (Rockstroh, Dejesus et al. 2013), elvitegravir (Sax, DeJesus et al. 2012) and dolutegravir (van Lunzen, Maggiolo et al. 2012) against efavirenz-based therapy have shown superior virological outcome at 48 weeks, faster time to undetectable viral load, lower incidence of adverse events, and fewer treatment discontinuations, and these findings have recently been confirmed in a meta-analysis (Messiaen, Wensing et al. 2013).

b) **Low risk for serious drug-drug interactions.**The potential for drug-drug interactions is significantly less for INSTIs compared with other antiretrovirals (relative risk compared

with raltegravir: boosted protease inhibitors [RR = 4.96], non-nucleoside reverse transcriptase inhibitors [RR = 2.48](Patel, Abdelsayed et al. 2011). These are particularly important considerations for low/middle income country settings where adults with newly diagnosed HIV infection often present with tuberculosis, or during antenatal screening in late pregnancy. Here DTG may carry significant advantages over NNRTIs, and other INSTIs. In the absence of alternatives to rifampicin-based TB therapy, DTG and raltegravir exposures are only moderately reduced (Dooley, Sayre et al. 2013), compared to larger (50-90%) reductions in concentrations of boosted PIs, nevirapine and elvitegravir. Dolutegravir is metabolized by glucuronidation (UGT1A1) with some contribution from CYP3A.

c) **Rapid viral load drop** potentially beneficial in late diagnosis during pregnancy. In Sub-Saharan Africa, pregnant mothers tend to engage with health services later in pregnancy compared with Europe, and new HIV diagnoses resulting from universal testing at ≥28w gestation are frequent. DTG results in a very rapid reduction in viral load; median time to undetectable viral load in the SINGLE study was 28 days vs. 84 days with Atripla ($P<0.0001$) (Walmsley, Antela et al. 2012). Use of DTG may lower risk of mother-to-child transmission of HIV in late presenters. Although meta-analysis shows no increase in birth defects with maternal efavirenz (Ford, Mofenson et al. 2010), there has been recent suspicion of neurological toxicity in a large French registry (Sibiude, Madelbrot et al. 2013) and neurodevelopmental delay in a South African cohort (Westreich, Rubel et al. 2010).

d) **Proven efficacy in patients with established drug resistance** to other antiretrovirals. In most resource-poor settings, options for anti-retroviral therapy are limited (2011), and the emergence of HIV drug resistance gives cause for concern (Hedt, Wadonda-Kabondo et al. 2008; WHO 2012). In a cross-sectional study conducted among HIV-1 antiretroviral naïve patients in five African countries, the highest prevalence of transmitted resistance was observed in Kampala, Uganda 12.3% (22 of 179; 7.5-17.1) (Hamers, Wallis et al. 2011). In Malawi, the prevalence of primary drug resistance in new infections was 6.1% which is comparable with figures of ~3% for South Africa (Manasa, Katzenstein et al. 2012), and 5.7% for Zambia (Hamers, Siwale et al. 2010), with over half of all mutations conferring resistance to non-nucleoside reverse transcriptases (NNRTI) (Wadonda-Kabondo, Banda et al. 2012). Studies across Africa have shown that treatment response to NNRTIs in mothers and children exposed to nevirapine (particularly single dose) during pregnancy is blunted (Musiime, Ssali et al. 2009). In this context, a novel class of ARV which is safe, affordable and effective is an urgent need

e) **Safety profile in pregnancy.**DTG is classified as FDA pregnancy Category B. There are no adequate and well-controlled studies in pregnant women. Because animal reproduction studies are not always predictive of human response, and dolutegravir was shown to cross the placenta in animal studies, the manufacturer recommends that this drug should be used during pregnancy only if clearly needed (ViiV 2013).

f) **Necessity of investigating DTG PK in pregnant women.** Alongside the clinical and humanitarian imperative to provide effective treatment for preventing HIV transmission in pregnant mothers with NNRTI-resistant HIV, there is an equally important ethical imperative to establish the safety, efficacy and pharmacokinetics of DTG in pregnant mothers and their breastfed infants in SSA, especially given the implementation of WHO Option B+, and the potential for widespread use of DTG in the forseeable future. Previous experience suggests it is universally the case that pregnant women in SSA receive new antiretrovirals (ARV) ahead of any proper evaluation, and clinical studies (if undertaken at

all) occur at a much later stage after many mothers and infants have been exposed. To ensure that this treatment does not risk therapeutic failure due to lower than anticipated exposure in pregnancy, intensive virological monitoring and other safeguards will be put in place for the first group of women enrolled in the study (see section 9.1), with scrutiny from the IDSMB.

g) **Importance of undertaking the study in SSA.** Any pivotal study if undertaken in developed countries would have limited generalizability to an African setting because i) ARVs tend to be initiated later in gestation in newly presenting pregnant women, ii) the standard of care across SSA is likely to be an efavirenz or nevirapine-based regimen iii) infant breastfeeding is recommended in the presence of ARVs ii) host factors such as BMI, pharmacogenomics, ethnicity etc may impact on pharmacokinetics (PK) of ARVs.

h) Continued administration of DTG for 2 weeks post-partum (whilst breastfeeding) to characterize PK in non-pregnant state. Under standard of care, the infant will receive 6 weeks of ART prophylaxis (as nevirapine with or without zidovudine), and would be exposed to the NNRTI/NRTI/NRTI combination received by the mother under standard of care. In this study, infants will be exposed DTG via the breastmilk for a 2 week period following birth. We believe this risk to be acceptable given the potential benefits. Furthermore, the risk is unlikely to exceed any potential risk of *in utero* exposure to DTG. Drug-drug interactions between directly administered NVP and DTG ingested via the breastmilk are unlikely to have clinical significance, although we will study this. The potential risks of breastfeeding are fewer than those of giving formula feeding during the two week period that mothers continue to receive DTG postpartum, but the mother will be switched to standard of care regimen (EFV-based) at 2 weeks postpartum to minimise any potential risks to the infant of prolonged low-dose exposure through breast milk. (This time point chosen as a compromise between reversion of maternal PK to baseline non-pregnant state against minimising any potential risks to the infant)Replacement of breast feeding with formula feeding during the first two weeks post-partum would be unethical due to the loss of the immune benefits of colostrum to the neonate, risks intrinsic to formula feeding in low resource settings and increased risk of HIV transmission if mixed feeding occurs (Teasdale et al, 2011) The potential risks of low-level ARV causing drug-resistance in the infant should the PMTCT fail exist for the standard of care NNRTIs and NRTIs; it is not anticipated that DTG will carry a greater risk but nevertheless this is best monitored within a clinical trial setting before widespread uptake without monitoring.

4.2 Risk Assessment for Dolutegravir in Pregnancy

FDA Category B. No studies have been conducted with DTG in pregnant women as pregnant women were excluded from the Phase IIb/III DTG clinical studies and subjects who became pregnant were required to discontinue from the studies.

Fifty-two pregnancies were reported across the DTG clinical studies and compassionate use program through 16Jan2016 (ViiV, unpublished data); 37 for DTG- containing ART and 15 for comparator. Of the 37 pregnancies in subjects receiving DTG, 21 were reported in the ART-naïve population (including one in a partner potentially exposed to DTG via the seamen of a male subject), 12 in the ART-experienced (integrase inhibitor [INSTI]-naïve) population, 3 in the ART experienced (INSTI-resistant) population and one was reported in a completed Phase I, healthy volunteer study (DTG).

Of the 52 pregnancies reported across the DTG clinical studies, 23 resulted in delivery of a normal healthy baby (including the partner pregnancy and a twin pregnancy), 13 in elective termination, one was ectopic (EFV/TDF/FTC) and 7 resulted in spontaneous abortions (3 for DTG, 2 for EFV/TDF/FTC and 1 each for RAL and DRV+RTV; all between 2 and 10 weeks gestation). In seven cases, the pregnancy was either ongoing or unknown (all DTG). During one pregnancy, involving an ART-experienced (INSTI-naïve) subject exposed to DTG plus abacavir, atazanavir and RTV, a routine ultra sound at 31 weeks gestation indicated a congenital anomaly of double outlet right ventricle with ventricular septal defect in the fetus. Surgery was performed following delivery but the Infant died on DOL 4 due to post-operative complications. The events were considered unrelated to IP by the reporting investigator. With the exception of this case, no other congenital anomalies have been reported. None of the cases resulting in adverse pregnancy outcome were considered reasonably attributable to IP by the reporting investigators. To date there has been no preclinical findings or evidence for an increased risk for teratogenic effects for DTG.

The overall nonclinical reproductive and developmental toxicity profile for DTG in rats and rabbits suggests that DTG is not teratogenic and has a low potential for fetal risk. There were no effects on fertility or early embryonic development in rats orally administered DTG at ~1000 mg/kg/day in males or females. The no adverse effect level (NOAEL) was 1000 mg/kg/day, which corresponds to ~33X the expected human exposure for a 50 mg once daily, based on gender averaged mean exposures achieved in the 4 week rat toxicity study. No adverse effects on fetal development were observed in pregnant rats orally administered DTG at ~1000 mg/kg/day. The NOAEL for maternal and fetal toxicity was1000 mg/kg/day, which corresponds to ~38X the expected human exposure for a 50 mg once daily dose. In an embryofetal development (EFD) study in rabbits, DTG was orally administered at 40, 200, or 1000 mg/kg/day to pregnant rabbits. Suppressed body weight gain (13.6% on gestation Day 19), decreased food consumption (up to 53%) and scant or no faeces/urine associated with the decreased food consumption were noted in the 1000 mg/kg/day dams. Maternal toxicity at this dose precluded dosing DTG at higher doses in rabbits because maternal toxicity can confound teratogenicity assessment. Therefore, the dose of 1000 mg/kg/day was the maximal dose that could be administered in this embryo fetal development EFD study.

The NOAEL was 200 mg/kg/day for maternal general toxicity (~0.27X the expected human exposure for a 50 mg once daily dose) and 1000 mg/kg/day for maternal reproductive function and embryofetal development (0.56X the expected human exposure for a 50 mg once daily dose,). Of note, there were no teratogenic effects at 1000 mg/kg/day, a dose that exceeded the NOAEL for maternal toxicity. In a pre-and postnatal development study, DTG was administered to female rats at doses of 5, 50 or 1000 mg/kg/day from Day 6 of gestation to Day 20 of lactation. Suppressed body weight gain and decreased food consumption were noted in dams (F0) in the 1000 mg/kg/day group during the lactation period, which were associated with mild decreases in body weights in the offspring in the 1000 mg/kg/day group from pre-weaning until adolescence. There were no adverse effects on maternal pregnancy, parturition, lactation or offspring (F1) survival, behavioral or reproductive function. The NOAEL for maternal reproductive function was 1000 mg/kg/day (~32X above the expected human exposure for a 50 mg once daily dose, based on exposures achieved in female rats in the 4 week toxicity study). Due to the decreased body weights of the offspring observed at higher doses, the NOAEL for

pre- and postnatal development of the offspring (F1) was 50 mg/kg/day. This is~25X above the expected human exposure for a 50 mg once daily dose (extrapolated from gender mean exposures achieved in the rat 14 day toxicity study). Based on the fact that effects on offspring body weights were noted at doses where maternal toxicity was observed, and the presence of considerable safety margins expected at the proposed clinical doses, there is minimal risk for adverse effects on postnatal development in offspring of mothers receiving DTG.

DTG is excreted in the milk of lactating rats. Following oral administration (50 mg/kg) to lactating rats on Day 10 postpartum, total radiocarbon concentrations in milk were up to 2-fold greater than those in maternal blood. The metabolite profile of milk indicated that parent DTG represented more than 95% of the total radiocarbon, consistent with the findings in plasma from female rats in an earlier study. These data suggest that F1 offspring in the pre- and postnatal toxicity study were exposed to the drug via the milk. Following oral administration of DTG (50 mg/kg) to pregnant rats on Day 18 post conception, DTG-related material was found, by quantitative whole body autoradiography (QWBA) analysis, to be widely distributed to the fetuses over the 24- hour sampling period. These data indicate that DTG is able to cross the placental barrier.

DTG is primarily metabolized through UGT1A1 with minimal renal excretion (<1% of total dose given orally). DTG is a drug with low clearance (~1 L/hr for CL/F after oral dosing), low volume of distribution (~12.5 L for Vd/F after oral dosing), and high plasma protein binding (>99%). The following physiologic changes during pregnancy may have impact on DTG PK

Table 1

Possible Physiological Changes During Pregnancy*	Potential Impact on DTG PK
Changes in total body weight and body fat composition.	Increase clearance (CL) as well as the volume of distribution (Vd)
Delayed gastric emptying and prolonged gastrointestinal transit time.	Increase oral bioavailability, delayed time to observed maximal drug concentration (tmax)
Increase in extra cellular fluid and total body water.	Increase Vd
Increased cardiac output, increased stroke volume, and elevated maternal heart rate.	Unlikely to have effect
Decreased albumin concentration with reduced protein binding.	Increase unbound fraction, CL, and Vd
Increased blood flow to the various organs (e.g., kidneys, uterus).	Unlikely to have effect

Increased glomerular filtration rate.	Unlikely to have effect
Changed hepatic enzyme activity, including phase I CYP450 metabolic pathways (e.g., increased CYP2D6 activity), xanthine oxidase, and phase II metabolic pathways (e.g., N-acetyltransferase	May affect CL

*FDA Guidance for Industry (2004): Pharmacokinetics in Pregnancy — Study Design, Data Analysis, and Impact on Dosing and Labeling.

DTG has shown to be not prone to changes by demographic factors (age, gender, weight, etc) based on population PK analyses. Accumulated safety data showed that DTG is in general well tolerated and no PK/Pharmacodynamic (PD) relationship for safety measures has been identified. Therefore, despite the potential impacts of physiological changes on DTG PK, it is expected that DTG PK should not change significantly during pregnancy. The clinical dose of 50mg once daily will be used in this study and no therapeutic drug monitoring is needed. However, to ensure that this assumption is true within the study population, the first 8 patients commenced on DTG will have their antepartum PK samples batched and shipped to Liverpool for analysis ahead of the first IDSMB meeting so that any clinically relevant reductions in AUC that might compromise effectiveness of the regime can be identified early.

4.3 Risk Management Plan

Table 2 sets out ethical considerations and safeguards which will be used in a series of community, clinical and scientific consultations:

Potential Risk of Clinical Significance Data/Rationale for Risk Mitigation Strategy	Potential Risk of Clinical Significance Data/Rationale for Risk Mitigation Strategy	Potential Risk of Clinical Significance Data/Rationale for Risk Mitigation Strategy
Fetuses are most susceptible to potential teratogenic effects of drugs during 1st trimester	With other ART agents, there are concerns with regard to teratogenicity (e.g., EFV) and maternal hepatic/rash toxicity (i.e. NVP).<br>ZDV, ABC and 3TC have high level of transplacental transfer.Data available for teratogenic risk for DTG is described in 4.3.	There is no specific mitigation of risk to the fetus. However, the risk is felt to be low based on all available data for DTG. Frequent visits to monitor clinical and laboratory parameters and routine care by the HIV care provider (via this protocol), and routine care by appropriate provider of healthcare to pregnant women
Pregnancy can increase risks of HIV progression, complications and perinatal transmission	The safe use of DTG in human pregnancy has not been established. DTG has been shown to cross the placenta in reproductive toxicity studies	Treatment of the underlying HIV disease. Additionally women taking ART during pregnancy can reduce risk of transmission to infant (i.e. early control of viral replication)
Hypersensitivity reactions (HSR) and rash	Drug-related hypersensitivity risk is established with current first line ART regimens in local use. HSR has been observed uncommonly with DTG. Rash was commonly reported in DTG Phase IIb/III clinical trials; episodes were generally mild to moderate in intensity; no episodes of severe rash, such as Stevens-Johnson Syndrome, toxic epidermal necrolysis and erythema multiforme were reported.	Specific/detailed toxicity management guidance is provided for suspected HSR with DTG (see Section 10.3.2) or rash with DTG (see Section 10.3.3)
Drug induced liver injury (DILI) and other clinically significant liver chemistry elevations	Non-clinical data suggested a possible, albeit low, risk for hepatobiliary toxicity with DTG. Drug-related hepatitis is considered an uncommon risk for ART containing DTG regardless of dose or treatment population. For subjects with hepatitis B (HBV) virus and/or hepatitis C (HCV) virus co-infection, improvements in immunosuppression as a result of HIV virologic and immunologic responses to DTG	Subjects meeting the following criteria during the screening period are excluded from participating (Section 4.3). Alanine aminotransferase (ALT) >5 times the upper limit of normal (ULN) or ALT >3xULN and bilirubin >2xULN (with >35% direct bilirubin).Subjects positive

	containing ART, along with inadequate therapy for HBV coinfected subjects, likely contributed to significant elevations in liver chemistries.	for HBV at screening (hepatitis B virus surface antigen positive [+HBsAg]) will have a full Hepatitis B panel performed and those with evidence of active disease (e antigen positive) will be excluded, as will those with an anticipated need for HCV therapy during the study.<br><br>Specific/detailed liver stopping criteria and toxicity management guidance is provided for suspected DILI or other clinically significant liver chemistry elevations (Section 10.3.1)
Gastrointestinal (GI) intolerance	Non- clinical studies showed upper and lower GI toxicity, including vomiting, diarrhoea and gastric erosions observed in monkey toxicology studies (thought to be related to local and not systemic toxicity). Mild to moderate GI intolerance (mainly diarrhoea and nausea) is associated with DTG treatment in a small proportion of subjects; however there were no indications of an increased risk for peptic ulcers or serious erosions.	Routine monitoring of GI symptoms will be performed.
Renal function	Mild elevations of creatinine have been observed with DTG which are related to a likely benign effect on creatinine secretion with blockade of OCT-2 receptor. DTG has been shown to have no significant effect on glomerular filtration rate (GFR) or effective renal plasma flow. Measurement of albumin/creatinine ratio confirmed there was no difference in the effect of DTG on albumin excretion compared with EFV or RAL. DTG is eliminated by renal excretion and exposure increases in patients with renal dysfunction.	Subjects with a Calculated eGFR <50ml/min will have modification of NRTI dose as per National Guidelines Specific/detailed toxicity management guidance is provided for subjects who develop a decline in renal function.
Creatine phosphokinase (CPK) elevations	Asymptomatic CPK elevations mainly in association with exercise have been reported with DTG therapy.	Specific detailed toxicity management guidance is provided for subjects who develop Grade 3 to 4 CPK elevations.

Increased occurrence of immune reconstitution inflammatory syndrome (IRIS)	With rapid HIV-1 RNA decline and early recovery of CD4+ cell counts there could,theoretically, be an increase in cases of IRIS.<br>The increased risk for HBV and HCV IRIS with DTG containing ART is addressed above; there was a low rate of other medical conditions frequently implicated in IRIS cases.	Subjects positive for HBsAb at screening or with active HCV illness (anticipated to require therapy) are excluded from participating. Subjects will have routine laboratory monitoring
Psychiatric disorders	Psychiatric disorders including suicidal ideation and behaviours are common in HIV infected patients. The psychiatric profile for DTG (incl. suicidality, depression, bipolar and hypomania, anxiety and abnormal dreams) was similar or favourable compared with other ART. The reporting rate for insomnia was statistically higher for blinded DTG+ABC/3TC compared to EFV/TDF/FTC in ING114467;however, this was not duplicated in any other Phase IIb/III study conducted with DTG.	Subjects who in the investigator’s judgment, poses a significant suicidality risk, will be excluded from participating in the study. Because of the elevated risk in the HIV- infected population, treatment emergent assessment of suicidality will be monitored during this study. Investigators are advised to consider mental health consultation or referral for subjects who experience signs of suicidal ideation or behaviour.

5 DESIGN, OBJECTIVES AND ENDPOINTS

5.1 Study Design

Open label randomized trial of DTG in late pregnancy. HIV+pregnant women (untreated at ≥28-36 weeks gestation will be randomized 1:1 to receive a DTG-based regimen compared with standard of care (regimen not containing INSTI).

5.2 Primary Objectives

1) To investigate the steady-state pharmacokinetics of DTG in HIV infected women during the third trimester of pregnancy and after two weeks post-partum

5.3 Secondary Objectives

1) To evaluate safety and tolerabilityof DTG in pregnant women
2) To determine the proportions of bound and unbound drug
3) To explore changes in PK between the third trimester of pregnancy and post-partum
4) To evaluate placental transfer of drug, using cord blood samples
5) To evaluate drug exposure in breastmilk with mother at steady-state (2 weeks postpartum)
6) Evaluation of neonatal drug exposure at maternal steady state
7) Evaluation of time for infant levels to become undetectable

   Whilst the study has been powered to conclusively address the PK objectives listed above, the following data will also be collected to allow a complete description of the cohort.

8) Proportion of mother to child transmission in each arm at six months postpartum
9) Pharmacogenomic factors influencing transplacental and breastmilk transfer of drug

5.4 Primary Endpoint

$AUC_{0\text{-}24}$of DTG in pregnant women in third trimester and 2 weeks postpartum

5.5 *Secondary Endpoints

- Safety and tolerability of DTG
- Proportion of women in each arm with VL< 50 copies/mL, and <400 copies/mL at delivery
- Change in viral load over the first 4 weeks of therapy
- PK of the unbound fraction of drug
- Cord:maternal plasma DTG ratio
- Maternal plasma: breastmilk DTG ratio at steady state, and at 1, 2 and 3 days after discontinuation of DTG (and transfer onto standard of care ARVs)
- Infant DTG levels at maternal steady state (2 weeks postpartum) and at 1, 2 and 3 days after maternal discontinuation of DTG
- Incidence and severity of adverse events and laboratory abnormalities
- Absolute values and changes over time in laboratory parameters

- Proportion of subjects who discontinue treatment due to adverse events
- Prevention of mother to child transmission (evaluated as six months postpartum)
- Potential pharmacogenomic factors affecting DTG PK in pregnancy and transfer to infant via placenta or breastmilk

6 SUBJECT POPULATION

6.1 Number of subjects

Previous data on 50mg qd DTG dosing in North American HIV-infected non-pregnant adults yielded an AUC 43.4µg.h/mL at steady state in healthy volunteers (CV 20%)(Min, Sloan et al. 2011). No data exist regarding within patient variability, but this is unlikely to exceed inter-individual variability. Assuming the variance holds true for HIV-positive pregnant African women, recruitment of 30 subjects would yield >95% power (paired design) to show a difference of +25% difference in mean AUC (the FDA upper boundary for bioequivalence) at α=0.05. However data forother antiretrovirals, such as the protease inhibitors, suggest that variance is greater in pregnant women. Sensitivity analyses of power-sample size relationships across a range of variances (CV 20-50%) are shown below.

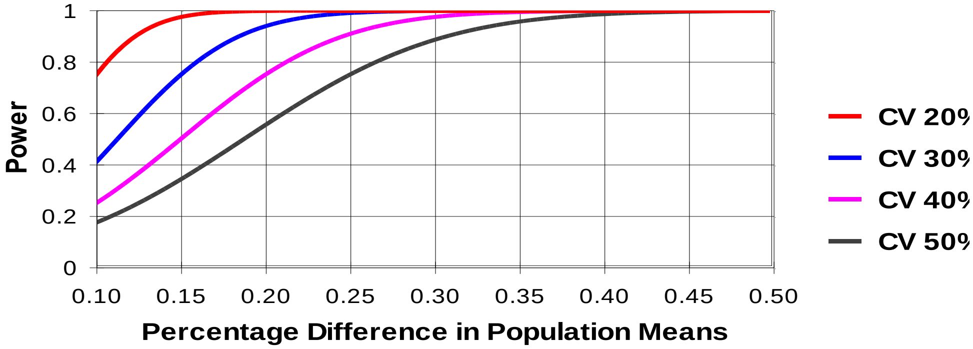

We have judged that recruitment of 60 pregnant women (30 on DTG) would a) allow us to detect with 80% power a dolutegravir AUC difference of 12% (CV20%), 16% (CV 30%), 22% (CV 40%), 27% (CV50%), so that even with doubling in CV, there is >80% power to detect change in AUC outside the FDA bounds for bioequivalence [0.8-1.25] and b) allow meaningful comparison in virological dynamics between DTG and standard of care.

6.2 Inclusion criteria

Participants must meet all of the following inclusion criteria to be eligible for enrollment into the study:

1. Evidence of a personally signed and dated informed consent document indicating that the participant (or a legal representative) has been informed of all pertinent aspects of the study.
2. Participants who are willing and able to comply with scheduled visits, treatment plan, laboratory tests, and other study procedures.
3. Women age 18 years or older
4. Pregnant

5.Untreated HIV infection in late pregnancy(≥28 weeks and <36 weeks gestation) (NB first 8 patients (the first 4 on each arm) will be between 28 and 32 weeks gestation)

6.3 Exclusion criteria

Participants presenting with any of the following will not be included in the study:

1. Received any antiretroviral drugs in previous 6 months
2. Ever received integrase inhibitors
3. Serum hemoglobin <8.0 g/dl
4. eGFR<50 ml/min*
5. Elevations in serum levels of alanine aminotransferase (ALT) >5 times the upper limit of normal (ULN) or ALT >3xULN and bilirubin >2xULN (with >35% direct bilirubin).
6. Active hepatitis B infection (patients with chronic stable HBV infection evidenced by positive HBsAg without evident clinical consequences remain eligible)*
7. History or clinical suspicion of unstable liver disease (as defined by the presence of ascites, encephalopathy, coagulopathy, hyperbilirubinaemia, oesophageal or gastric varices or persistent jaundice).
8. Subjects with severe pre-eclampsia (e.g. HELLP), or other pregnancy related events such as renal or liver abnormalities (e.g. grade 2 or above proteinuria, elevation in serum creatinine (>2.5 x ULN),total bilirubin,ALTor AST)*
9. Paternal non-consent for infant participation (where disclosure has taken place – see below)
10. Clinical depression or clinical judgment suggests increased risk of suicidality

*Hb, eGFR, ALT, bilirubin and Hepatitis B serology will be undertaken at Screening, with the results made available at the earliest opportunity.

6.3.1 Considerations of Paternal Consent (Ugandan Site Only with reference to UNCST guidance)

Whilst the father is a key stakeholder in a pregnancy, and in an ideal world, would be involved in all decisions relating to the wellbeing of both mother and child, this is not a clear-cut issue. A woman diagnosed with HIV in late pregnancy is a vulnerable individual, whom we have a duty of care to protect. It may not be in her best interests to immediately disclose her positive HIV status to her male partner, as this may risk violence and potential abandonment. It is therefore not possible to establish clear inclusion and exclusion criteria relating to paternal consent, although if a male partner who is cognisant and supportive of the mother's HIV treatment expresses that he does not wish for participation in the study to proceed due to the potential risks of exposure of his unborn child to DTG, this shall be viewed as an exclusion criterion.

The mother shall be asked whether she gives consent for her partner to receive an abbreviated version of the PIL (Appendix 3); this shall contain the fundamental details of the study, the contact details of members of the study team and space for a signature to confirm that the father has read and understood this information. No witness signature is required as this is not a legal process.

6.4 Selection of Participants

Patients will be identified and screened at the antenatal clinics associated with the study. Pharmacokinetic sampling and subsequent study visits will be conducted at IDI

6.5 Withdrawal of subjects

A subject is free to withdraw from the study at any time. In addition, the Investigator may decide, for reasons of medical prudence, to withdraw a subject. Safety bloods and HIV viral load results will be communicated to the clinicians responsible for routine care of the patients, and they can choose to withdraw a patient from the study at any time. If a subject discontinues study medication dosing, every attempt should be made to keep the subject in the study and continue to perform the required study-related procedures and follow-up procedures. If this is not possible or acceptable to the subject or Investigator, the subject may be withdrawn from the study.
Study medication may be discontinued in the following instances:

- If the subject withdraws her consent.
- If the Investigator considers in the interest of the subject (i.e. intercurrent illness, unacceptable toxicity) that it is best for herto withdraw herconsent.
- The subject's clinician considers it in their best interests
- The subject fails to comply with the protocol requirements or fails to cooperate with the Investigator.
- The subject starts treatment with any disallowed medication without prior notification and consent of the Investigators.
- The subject experiences a liver event (Section 10.3.1), hypersensitivity reaction (Section 10.3.2) or severe skin rash (Section 10.3.3).
- The HIV viral load has not declined sufficiently during the first four weeks of therapy (see detail in Sections 8.3 and 9.1)

The date and reasons for the withdrawal will be clearly stated on the subject's CRF. Non-compliant subjects may be withdrawn and replaced at the discretion of the investigator.

7 STUDY TREATMENTS

7.1 Treatment Plans and Regimens

1) Dolutegravir group (DTG+2 NRTIs) – to make best comparison with standard of care, these NRTIs should be those recommended by national policy.
   Patients randomized to the study drug will be commenced on an antiretroviral regimen comprising DTG 50mg once daily in combination with 2 NRTIs
2) Standard of care (SoC)
   Patients randomized to receive standard of care will receive the currently used antiretroviral regimens in keeping with national policy.

3) Backbone 2 NRTIs at treating physician's discretion, according to National guidelines. Those with positive HBsAg will routinely be offered TDF and 3TC (this is in fact the standard of care NRTI backbone in both Uganda and South Africa)

Additional Considerations for Hepatitis B (HBV) co-infected patients: Investigators should consult current treatment guidelines (e.g., IAS) when considering choice of NRTIs to combine with DTG for subjects with chronic HBV infection (HBsAg positive OR anti-HBc positive with HBV DNA present). In addition, clinical trial and marketed use of 3TC, FTC and TDF have shown that some subjects with chronic HBV disease may experience clinical or laboratory evidence of recurrent hepatitis upon discontinuation of 3TC, FTC or TDF, which may have more severe consequences in subjects with decompensated liver disease. If 3TC, FTC or TDF is discontinued in subjects co-infected with HBV, periodic monitoring of both liver chemistry tests and markers of HBV replication should be considered.

7.2 Dolutegravir

In August 2013, the FDA approved DTG for HIV-1 treatment in adults and children over 12 years of age and weighing > 40kg. Appendix A contains detailed safety data for Tivicay(ViiV 2013). Dolutegravir is available as 50mg tablets which do not require any special storage conditions. The absolute bioavailability has not been established. Peak concentrations of DTG are achieved 2.5 – 4.5 hours post dose, with no significant effect of diet (Song, Borland et al. 2012), and the terminal half-life of 15 hours supports once daily dosing without the need for a boosting agent (Castellino, Moss et al. 2013)

The safety assessment of DTG in HIV-1 infected treatment naïve subjects is based on the analyses of 48 week data from two ongoing, international, multicentre double blind trials, SPRING-2 (ING113086) (van Lunzen, Maggiolo et al. 2012; Raffi, Rachlis et al. 2013) and SINGLE (ING114467) (Walmsley, Antela et al. 2012) in which a total of 1655 patients received DTG, in combination with 2 NRTIs. The nature and frequency of adverse events are summarized in Table 2.

In SPRING-2, involving 822 patients across 100 sites in North America, Europe and Australia, dolutegravir (coformulated with tenofovir/emtricitabine or abacavir/lamivudine) compared favourably with raltegravir both in terms of virological non-inferiority and with a comparable adverse event profile. The most commonly reported adverse effects were minor gastrointestinal disturbance (nausea or diarrhea), headache and nasopharyngitis. In both raltegravir and dolutegravir study arms, 2% of patients suffered adverse events necessitating a change in regimen. Three patients receiving DTG suffered a serious adverse event, namely aphasia, diarrhoea and hepatitis, each occurring in a single subject; no deaths related to the study drug (Raffi, Rachlis et al. 2013).Table 2: Treatment-emergent adverse drug reactions of at least moderate intensity (Grades 2 to 4) and ≥2% frequency in treatment-naïve subjects in SPRING-2 and SINGLE trials (week 48 analysis).

7.1 Prior and Concomitant Medication

Patients should continue to take their prescribed medication, unless it is specifically contraindicated to take this medication with dolutegravir as indicated in the prescribing information for DTG. Both prior and concomitant medication prescribed during the course of the study will be recorded on the CRF.

Patients who are concurrently prescribed medication containing divalent cations, such as ferrous sulphate or antacids, will be given specific advice to ingest dolutegravir either two hours before or six hours after these medications.

Table 3

System Organ Class/ Preferred Term	SPRING-2		SINGLE	
	TIVICAY 50 mg Once Daily + 2 NRTIs (N = 403)	Raltegravir 400 mg Twice Daily + 2 NRTIs (N = 405)	TIVICAY 50 mg + EPZICOM Once Daily (N = 414)	ATRIPLA Once Daily (N = 419)
**Psychiatric**				
Insomnia	<1%	<1%	3%	2%
Abnormal dreams	<1%	<1%	<1%	2%
**Nervous System**				
Dizziness	<1%	<1%	<1%	5%
Headache	<1%	<1%	2%	2%
**Gastrointestinal**				
Nausea	1%	1%	<1%	3%
Diarrhea	<1%	<1%	<1%	2%
**Skin and Subcutaneous Tissue**				
Rash[a]	0	<1%	<1%	6%
**Ear and Labyrinth**				
Vertigo	0	<1%	0	2%

7.2 Infant Nevirapine

Under routine PMTCT care, infants will be administered nevirapine syrup (with or without zidovudine/3TC) for the first four to twelve weeks of life (depending on risk stratification). These infants will potentially be exposed to a small dose of DTG via breastfeeding (data regarding the breastmilk elimination of DTG are not available, hence this is an important secondary endpoint of DolPHIN). As DTG is a substrate of UGT1A1 with some contribution from CYP3A, NVP has the potential to decrease the levels of DTG, but this is unlikely to be of clinical significance as the infant DTG is not intended to be of therapeutic benefit.

8 STUDY SCHEDULES AND PROCEDURES

The schedule of assessments for each subject enrolled in the study is presented in the study flowchart and detailed in the text below. The shaded section illustrates how proposed study visits relate to the scheduled antenatal and postnatal care of the mother and infant; the study design has taken into consideration the logistical challenges of mothers attending clinic appointments in the early neonatal period and has sought to overlap with the scheduled visits occurring under standard of care.

8.1 Study Schedule

Study Procedure	Screening	Randomisation	Study Period (Weeks post DTG start)									
**Time**	**0 (~28 weeks gestation)**	**1-7 days post screening**	**1**	**2**	**4**	**8**		**+2 wks post partum**	**1 day post change to SoC**	**2 days post change to SoC**	**3 days post change to SoC**	**End of Study***
Routine Obstetric Care	ANC V2 (24-28 weeks)		ANC V3 (32-36 weeks)			ANC V4 (> 36 weeks)		Postnatal visit 1 (~1 week post)				Postnatal visit 2 (~6 months post)
Recruitment	X						**Estimated date of delivery**					
Informed Consent	X											
Clinical assessment[1]	X		X	X	X	X		X	X	X	X	
Safety questionnaire - mother	X		X	X	X	X		X	X	X	X	
Safety questionnaire - infant								X	X	X	X	
Safety bloods[2]	X		X	X	X	X		X				
HIV viral load	X	X[5]		X	X			X				
CD4 count	X							X				
Blood for maternal genomics				X								
Randomisation 1:1 DTG vs SoC		X										
Randomisation 1:1 to return on Day 1-3 after switch to SoC								X				
Dispense SoC ART	X											
Dispense Study Treatment[3]		X	X	X	X	X		X				
Blood PK (DTG arm)[4]				X				X	X	X	X	
Cord& Maternal DTG level							X					
BM DTG level								X	X	X	X	
Infant DTG level								X	X	X	X	
Edinburgh Postnatal Depression Scale								X				X

*ANC =Antenatal Clinic *If additional visit required for follow-up beyond 2 weeks postpartum*

1 *Clinical assessment – clinical questionnaire, blood pressure, heart rate, respiratory rate, urinalysis*

2 *Safety bloods: FBC, U&E, LFT, eGFR, ALT*

3 *SoC will be dispensed at screen to comply with local guidelines. If the participant is assigned at randomisation to the DTG arm, she will be switched to that regimen at day 1.*

4 *Patients in EFV arm will not undergo rich PK sampling, but have a single blood sample taken for 'safety bloods' and HIV viral load.*

5 *For women who are switching from efavirenz to DTG after randomisation.*

8.2 Screening

Screening will take place in the antenatal clinics at the study sites. Any newly diagnosed patient will be counseled regarding their HIV diagnosis and the need to commence anti-retroviral therapy for life, both to reduce the risk of viral transmission to the infant, and for the health of the mother. Counseling will be undertaken by trained nurses and counsellors working in the ARTclinic. Whilst it is emphasized that the woman should commence ARTas soon as possible in order to increase the likelihood of an undetectable viral load by the time of delivery, it is also important to ensure the mother is engaged with treatment, understands the diagnosis, the need for treatment, the importance of robust adherence to therapy and the need to attend for follow-up visits at the appointed time. It is possible that this step cannot be completed in a single clinic visit on a single day, and therefore provision should be made for the patient to have a follow-up appointment within a few days.

Each potentially eligible subject must sign an Informed Consent Form prior to the conduct of any screening procedures. Subjects will be given the opportunity to ask any questions regarding the trial at this stage. Screening evaluations will be used to determine the eligibility of each candidate for study enrolment. Each subject being screened for study enrolment evaluation will be assigned a unique screening subject number. Only one unique identifier will be allocated to a subject.

The screening visit will evaluate:

- Demographic details including age, gender, ethnicity
- Full medical, drug (including contraceptives, herbal and recreational drugs) and social history
- Weight, height and vital signs (temperature, blood pressure, heart rate, and respiratory rate)
- Full blood count with a differential white cell count
- Chemistry panel including eGFR (calculated from Cockroft-Gault formula)
- Liver function tests
- HIV-1 and 2 antibodies
- CD4 count
- HIV viral load
- Hepatitis B antibodies
- Urinalysis
- Results of these ‘screening bloods’ will become available within a week from screening,with appropriate clinical care provided, regardless of continued participation.

Standard of care ART will be dispensed at screening, as policy requires women to start ART the same day they attend antenatal care and are diagnosed HIV-positive.

8.3 Randomisation

Once inclusion and exclusion criteria are complete, eligible subjects will be randomised into the 2 arms on a 1:1 ratio using the randomisation schedule. Because of the intensified safety monitoring of the first 16 patients enrolled in the study (as detailed under the specific visits below), a permuted block randomization will be undertaken to avoid asymmetrical allocation: block 1 (N=8); block 2 (N=8); block 3 (N=44). Subjects will be assigned to study treatment in

accordance with the computer generated randomization schedule. The randomization schedule will be generated using permuted block randomisation by Randomizer for Clinical Trials (Institute for Medical Informatics, Statistics and Documentation, Medical University of Graz).Randomisation can occur on the Day 1 visit or prior to that. The participant does not need to be present.

Following enrollment, subsequent study visits will take place at the research site, but there will be close liaison with the antenatal care clinics regarding the clinical care of the participants.

8.3.1 Drug dispensing visit, Day 1

Once the study team is satisfied that the patient understands her diagnosis, the need for treatment, and the importance of adherence, she will be provided with a 2 week supply of DTG+2NRTI or SoC and given both verbal and written instructions regarding their administration. **Those randomised to the DTG arm will switch from SoC to DTG and 2 NRTIs. Those randomised to SoC will remain on that regimen**. A further blood sample for HIV viral load will be taken from those subjects switching to DTG. She will be given a contact number whereby she can raise any questions with a member of the study team at any time. In the event of no further problems, a follow-up appointment shall be made for the following week.

8.3.2 Visit 2, Day 7 (tolerance +/- 1 day)

Clinical assessment will be undertaken, involving questions regarding the occurrence of any adverse events or difficulties in taking the medication, a physical examination, and performance of the panel of ‘safety’ bloods. If uneventful, a follow-up appointment shall be made for the following week.

8.3.3 Visit 3, Day 14 (tolerance +/- 2 days)

Patients randomized to the DTG arm will be asked to attend the research facility at 7.30 am, having not yet taken her morning dose of ART. Following a clinical assessment (including safety questionnaire), as detailed under Visit 2, Day 7, an intravenous cannula will be inserted into either antecubital fossa, and a trough blood sample taken at Time 0. Drug will then be administered with a standard breakfast and blood samples taken after 0.5, 1, 2, 3, 4, 6 and 8 hours (5ml on each occasion, making a total of 40ml). Blood for measurement of HIV viral load will also be taken*. The cannula will then be removed. The patient will be asked to attend at 08:00 the following day for a 24 hour blood sample (tolerance +/- 3 hours). Following this, the patient will be provided with a further 2 weeks medication, in addition to a reminder of how to contact the study team in the event of a query

Patients randomized to the SoC arm will have a clinical assessment identical to the DTG arm and blood taken for HIV viral load (these samples will be batched rather than analysed in real-time).

*HIV viral load will be processed with results within 7 days for the first eight patients in the DTG arm with the appropriate action on the result being detailed in Section 9.1

8.3.4 Visit 4, Day 28 (tolerance +/- 7 days)

Clinical assessment will be undertaken, involving questions regarding the occurrence of any adverse events or difficulties in taking the medication, a physical examination, and performance of the panel of 'safety' bloods. Blood for HIV viral load will be taken.If uneventful, a follow-up appointment shall be made for the four weeks' time. She shall be reminded of plans for labour and delivery, and provided with written information to take with her to the labour ward containing details of the study and how to contact the study team, in addition to the information she has already received.

8.3.5 Visit 5, Delivery

Following delivery, a sample of cord blood will be taken to measure DTG levels and a corresponding sample of blood will be obtained from a mother. If the birth has been uncomplicated, the patient will be given an appointment to attend the research facility in a further two weeks.In the South African centre, where possible, participation will be documented in the infant 'road to health' booklet.

8.3.6 Visit 6, 2 weeks post-delivery (tolerance +/- 7 days)

This study visit will coincide with the 1 week post-delivery assessment which will occur under SoC. This visit will focus on safety endpoints, including maternal bloods and a neonatal review of the infant by the Study Paediatrician.Both maternal and infant safety questionnaires (Appendix 3) will be administered in addition to the Edinburgh Postnatal Depression Scale (Appendix 5). Patients receiving SoC will be asked to provide a blood sample for HIV viral load. In the South African centre, if not already done postpartum, participation in the study will be documented in the infant 'Road to Health' booklet.

Patients randomized to the DTG arm will be asked to attend the research facility at 7.30 am, having not yet taken her morning dose of ART. Following a clinical assessment, as undertaken on previous study visits, an intravenous cannula will be inserted into either antecubital fossa,and a trough blood sample taken at Time 0. Drug will then be administered with a standard breakfast and blood samples taken after 0.5, 1, 2, 3, 4, 6 and 8 hours (5ml on each occasion, making a total of 40ml). Blood for CD4 count and HIV viral load will also be taken. The cannula will then be removed. Blood (0.5mL) will also be sampled from the baby on two occasions to evaluate neonatal DTG exposure (one timed relative to a feed at anticipated maternal tmax, and one at a random time point relative to maternal dosing). The mother will be asked to provide two samples(3-5 ml) of manually expressed breast milk to measure the elimination of DTG in breast milk. The subject will be asked to attend at 08:00 the following day for a 24 hour blood sample (tolerance +/- 3 hours).

Following this visit, the mother will be counselled with regard to Standard of Care (SoC) antiretroviral therapy and follow-up schedules. DTG will be discontinued and she will be commenced onto the recommended NNRTI-based first-line regimen.

Participants will then be randomized 1:1:1 to attend the following day, two days or three days later for a further single blood sample to measure DTG levels in maternal blood, breast milk and infant plasma.

8.3.7 Visit 7, 1, 2 or 3 days following switch to Standard of Care (SoC)

Mothers will be assessed to ensure there have been no unexpected difficulties with regard to commencing SoC ART. A single blood sample from the mother and heel-prick blood spot from the infant will be taken for DTG levels. A sample of BM will also be collected for DTG measurement.

8.3.8 Visit 8, month 6 post-delivery (tolerance +/- 1 month)

We will incorporate an additional postnatal visit for both mother and infant, at 6 months following delivery. At this time point, a detailed neonatology evaluation will be conducted by the study paediatrician.
If following full clinical assessment, there are no outstanding issues relating to the study, the End of Study formswill be completed and the patient transferred to Standard of Care HIV management, including first line national policy ARTand follow-up within the ART clinic.

8.4 Clinical Assessment/Early Termination Visit

For early study termination, the mother (and infant) will be invited for review 10 weeks after finishing study medication or early termination. The following evaluations will be performed:Vital signs (temperature, blood pressure, heart rate, and respiratory rate)

- Haematology with a differential white blood cell count
- Chemistry panel
- Review of Adverse Events
- Concomitant medications review

The reason for the early termination of the subject should be clearly documented on the subject’s CRF. The subject will not then be required to attend for a follow up visit unless deemed necessary in the opinion of the investigator (e.g. due to adverse event or to determine pregnancy outcome status if still pregnant at the time of early termination).
Following completion of DTG, the patient should be transferred on to the routinely administered ART combination which comprises standard of care and followed-up by the antiretroviral clinic in accordance with National Guidelines. In many cases, this will be to an EFV-based regimen; using cross-study comparisons to historical data, DTG does not appear to affect the pharmacokinetics of this agent and therefore a wash-out period is not necessary. EFV has the potential to reduce DTG levels, and therefore a clinically significant interaction is unlikely.

9 SAFETY MONITORING

Safety assessments will be conducted to characterize the safety and tolerability of
DTG administered during pregnancy, including monitoring and recording all AEs and SAEs, regular monitoring of haematology and blood chemistry, physical exams and monitoring and recording birth and maternal outcomes.

9.1 Intensive Virological Monitoring of First 8 Patients receiving DTG

To ensure that use of DTG-based ART in pregnancy does not risk therapeutic failure due to lower than anticipated exposure in pregnancy, three additional safeguards will be put in place for the first group of women enrolled in the study:

1) The first eight patients to be randomized (the first four on DTG) will be at 28-32 weeks gestation, allowing sufficient time for a switch to standard of care before delivery if required. Viral loads will be undertaken in real time (turnaround time 7 days; baseline samples may be batched with week 2 sample). The IDSMB will review virological data from these 4 mothers on DTG. The following criteria for virological response will be utilized:

At 2 weeks

HIV viral load	Action
<1 log_{10} reduction or remains ≥10 000 copies/ml	Assess adherence or factors affecting absorption<br>Further viral load in real-time at Day 28 visit
>1 log_{10} reduction and <10 000 copies/ml	No need for further real-time VL monitoring

At 4 weeks

HIV viral load	Action
<1 log_{10} or evidence of blunting of virological response between weeks 2 and 4	Immediate switch to the Standard of Care EFV-based regimen
>1 log_{10} reduction, but ≥ 10 000 copies/ml	Continue DTG, but perform HIV VL two-weekly until <1000 copies/ml
>1 log_{10} reduction and <10 000 copies/ml	No need for further real-time VL monitoring

Patients and their clinicians may choose to withdraw participation at any point, and the clinical judgement around what constitutes an adequate virological response will also involve the treating medical team.

2) The next eight mothers to be randomized will have the same virological scrutiny (for the 4 women assigned to DTG) but may include women presenting up to 36 weeks gestation, as per the eligibility criteria of the study. These data will also be scrutinized by the IDSMB.

3) Recruitment will be suspended pending IDSMB review, and the IDSMB may choose at any point to request suspension of recruitment if it considers safety a major concern.

4) PK samples collected two weeks after start of therapy on these 8 women receiving DTG will be analysed for the interim IDSMB review to ensure that the AUC is not reduced to a level which might risk therapeutic failure.

9.2 Maternal Assessments

Laboratory baseline screening is outlined in Section 8 and includes:

Full blood count (including automated WBC differential, platelets, haemoglobin, haematocrit), Urea, sodium, potassium, creatinine, creatinine phosphokinase, bilirubin, glucose.Glomerular Filtration Rate (GFR) will be estimated. Blood will be stored for maternal plasma HIV viral load and resistance assays. Urinalysis (dipstick).

Clinical screening is also outlined in Section 8.

The Edinburgh Postnatal Depression Scale (Appendix 5) will be administered at the early postnatal visit, with prompt liaison with psychiatric services should a risk of suicidality be identified. At IDI, Kampala, there is a regular mental health clinic within the HIV Prevention, Care and Treatment centre. At DTHF the participant would be assessed by the DTHF unit psychologist and referred to the psychiatric services at the Gugulethu site. These services include a psychiatric sister and a mental health clinic managed by a psychiatric registrar. See also Section 10.3.4.

.

9.3 Infant Assessment

The following assessment information will be collected infants: Full blood count (including automated WBC differential, platelets, haemoglobin, haematocrit), urea, sodium, potassium, creatinine, creatinine phosphokinase, bilirubin, glucose within the first two weeks of birth.
In addition, if possible, the infant outcomes will also be captured and the following parameters willbe captured on the neonate at the time of delivery:

- Mode of delivery, length of rupture of membranes, use of foetal scalp monitors, and any complications, since can alter transmission risk.
- Gestational age
- Neonatal length and weight
- Neonatal head circumference
- Absence or presence of congenital malformations, with description ofmalformations where present
- APGAR scores
- IUGR

Extended follow-up of infants. Between 3-6 months of age a paediatric assessment of infants will be offered to all participants. This is not mandated by the protocol. Assessments will be performed as per local guidelines with including a history and physical examination. Infant laboratory assessments performed at baseline will be repeated.

10 MANAGEMENT OF ADVERSE EVENTS

10.1 Adverse Events

An adverse event (AE) is any untoward medical occurrence in a subject administered a pharmaceutical product and which does not necessarily have a causal relationship with the study treatments. An AE can therefore be any unfavourable and unintended sign (including an abnormal laboratory finding), symptom, or disease temporally associated with the use of a medicinal (investigational) product, whether or not considered related to the medicinal (investigational) product.

AEs observed by the Investigator, or reported by the subject, and any remedial action taken, will be recorded in the subject's CRF and should be verifiable in the subject's notes throughout the study. The nature of each event, time of onset after drug administration, duration and severity will be documented together with the Investigator's opinion of the causal relationship to the treatment.

Procedures such as surgery should not be reported as AEs. However, the medical condition for which the procedure was performed should be reported if it meets the definition of an AE. For example, an acute appendicitis that begins during the AE reporting period should be reported as the adverse event and the resulting appendectomy noted on the CRF. Planned procedures such as surgery planned prior to the subject's enrolment into the study need not be reported as AEs if these are documented as planned at the screening visit.

Clinically significant changes in physical examination and blood safety profiles should also be recorded as AEs.

Severity should be recorded and graded according to the **Division of Aids Table for Grading the Severity of Adult and Paediatric Adverse Events** (2004)(Appendix 2).

Note: There is a distinction between the gravity and the intensity of an AE. Severe is a measure of intensity; thus, a severe reaction is not necessarily a serious AE. For example, a headache may be severe in intensity but would not be classified as serious unless it met one of the criteria for serious events.

The relationship to the study drugs of each AE will be assessed using the Liverpool Causality Tool (Appendix 3) with the following definitions:

DEFINITE: distinct temporal relationship with drug treatment. Known reaction to agent or chemical group, or predicted by known pharmacology. Event cannot be explained by subject's clinical state or other factors.

PROBABLE: reasonable temporal relationship with drug treatment. Likely to be known reaction to agent or chemical group, or predicted by known pharmacology. Event cannot easily be explained by subject's clinical state or other factors.

POSSIBLE: reasonable temporal relationship with drug treatment. Event could be explained by subject's clinical state or other factors.

UNLIKELY: poor temporal relationship with drug treatment. Event easily explained by subject's clinical state or other factors.

UNRELATED: the event occurs prior to dosing. Event or intercurrent illness is due wholly to factors other than drug treatment.

All AEs, however minor, will be documented in the CRF whether or not the Investigator concludes the event to be related to drug treatment.

The AE reporting period will be from the screening visit until the subjects final study visit. In addition, any untoward event that may occur within 30 days of discontinuation of investigational product that the Investigator assesses as possibly, probably or definitely related to the study drug medication should also be reported as an AE.

AEs may be directly observed, reported spontaneously by the subject or by questioning the subject at each study visit.

All AEs should be followed up until they are resolved or the subject's participation in the study ends (i.e. until the final CRF is completed for that subject). In addition, all serious and non-serious AEs assessed by the Investigator as possibly related to the investigational medication should continue to be followed even after the subject's participation in the study is over. Such events should be followed until resolution, or until no further change can reasonably be expected. Deaths occurring more than 30 days after the final dose, which are considered to be unrelated to the study medication, should not be reported as a Serious Adverse Event.

10.2 Serious Adverse Events

A serious adverse event (SAE) is any untoward medical occurrence that at any dose:

i) Results in death
ii) Is life threatening
iii) Requires in patient hospitalisation or prolongation of existing hospitalisation
iv) Results in persistent or significant disability/ incapacity, or
v) Is a congenital anomaly/ birth defect.
vi) All events of possible drug-induced liver injury with hyperbilirubinemia defined as ALT ≥3xULN and bilirubin ≥2xULN (>35% direct) in patients treated with DTG.

NOTE: bilirubin fractionation is performed if testing is available. If testing is unavailable, record presence of detectable urinary bilirubin on dipstick indicating direct bilirubin elevations and suggesting liver injury. If testing is unavailable and a subject meets the criterion of total bilirubin ≥2xULN, then the event is still reported as an SAE. If PT is obtained, include values on the SAE form. PT elevations >1.5 x ULN suggest severe liver injury.

Liver events: Liver event CRFs and liver imaging and/or liver biopsy CRFs (see Section 10.3.1) should be completed and reported to the Principal Investigator within one week of event onset.

Suicidal Ideation or Behaviours: If any subject experiences a possible suicidality-related adverse event (PSRAE) while participating in this study that is considered by the Investigator to meet International Conference on Harmonization (ICH)-E2A definitions for seriousness, the Investigator will collect information using a PSRAE CRF form in addition to reporting the event on a SAE CRF form. A PSRAE may include, but is not limited to, an event that involves suicidal ideation, a preparatory act toward imminent suicidal behaviour, a suicide attempt, or a completed suicide. The investigator will exercise his or her medical and scientific judgment in deciding whether an event is possibly suicide-related. PSRAE forms should be completed and reported to the Principal Investigator within one week of the investigator diagnosing a possible suicidality-related serious adverse event.

10.3 Specific Toxicities/Adverse Event Management

General guidelines for the management of specific toxicities that are considered to be related or possibly related to DTG are provided below.

Subjects who permanently discontinue DTG for reasons of toxicity should be followed weekly until resolution of the AE and encouraged to complete the withdrawal and FU study evaluations as noted above.

10.3.1 Liver chemistry stopping and follow up criteria

Liver chemistry threshold stopping criteria have been designed to assure subject safety and to evaluate liver event etiology during administration of IP and the follow-up period. IP will be stopped if any of the following liver chemistry criteria are met

- ALT ≥3xULN and bilirubin ≥2xULN (>35% direct bilirubin; bilirubin fractionation required)
    - NOTE: serum bilirubin fractionation should be performed if testing is available. If testing is unavailable, record presence of detectable urinary bilirubin on dipstick, indicating direct bilirubin elevations and suggesting liver injury. If testing is unavailable and a subject meets the criterion of total bilirubin ≥ 2xULN, then the event meets liver stopping criteria;
- ALT ≥8xULN;
- ALT ≥3xULN (if baseline ALT is < ULN) with symptoms or worsening of acute hepatitis or hypersensitivity such as fatigue, nausea, vomiting, right upper quadrant pain or tenderness, fever, rash, or eosinophilia, OR;
- ALT ≥3x baseline ALT with symptoms or worsening of acute hepatitis or hypersensitivity such as fatigue, nausea, vomiting, right upper quadrant pain or tenderness, fever, rash, or eosinophilia;
- ALT ≥5xULN and <8xULN that persists > 2 weeks (with bilirubin <2xULN and no signs or symptoms of acute hepatitis or hypersensitivity);
- ALT ≥5xULN but <8xULN and cannot be monitored weekly for >2 weeks;

Subjects who develop ALT ≥5xULN should be followed weekly until resolution or stabilization (ALT <5xULN on 2 consecutive evaluations).

When liver chemistry stopping criterion is met, do the following:

- **Immediately discontinue DTG and withdraw the subject from the study. Subjects should not restart DTG due to the risk of a recurrent reaction.**
- Evaluate for HELLP (Hypertension Elevated Liver enzymes Low Platelets) Syndrome and pregnancy-related steatosis in subjects with ongoing pregnancy.
- Notify the Principal Investigator of the event within 24 hours of learning its occurrence;

- Events of possible drug-induced liver injury with hyperbilirubinemia defined as ALT ≥3xULN and bilirubin ≥2xULN (>35% direct) will be reported to the Principal Investigator as SAEs using the SAE CRF (see Section 10.2).
- Complete the liver event CRF for all events meeting liver stopping criteria, and report to the Principal Investigator within one week of first becoming aware of the event (see Section 10.2);
- Complete the liver imaging and/or liver biopsy CRFs if these tests are performed, and report to the Principal Investigator (see Section 10.2);
- Perform liver event follow up assessments (described below), and monitor the subject until liver chemistries resolve, stabilize, or return to baseline values as described below;
- Make every reasonable attempt to have subjects return to clinic within 24 hours for repeat liver chemistries, liver event follow up assessments (see below), and close monitoring;
- A specialist or hepatology consultation is recommended;
- Monitor subjects twice weekly until liver chemistries (ALT, AST, alkaline phosphatase, bilirubin) resolve, stabilize or return to within baseline values;

Consider the following additional tests to further evaluate the liver event:

- Viral hepatitis serology including:
  - Hepatitis A IgM antibody;
  - HBsAg and Hepatitis B Core Antibody (IgM);
  - Hepatitis C RNA;
  - Hepatitis E IgM antibody;
- Cytomegalovirus IgM antibody;
- Epstein-Barr viral capsid antigen IgM antibody (or if unavailable, obtain heterophileantibody or monospot testing);
- Syphilis screening
- Drugs of abuse screen including alcohol
- Serum acetaminophen test (APAP adduct test)
- Serum creatine phosphokinase (CPK) and lactate dehydrogenase (LDH);
- Fractionate bilirubin, if total bilirubin is greater than 1.5xULN;
- Obtain complete blood count with differential to assess eosinophilia;
- Anti-nuclear antibody, anti-smooth muscle antibody, and Type 1 anti-liver kidney microsomal antibodies;
- Liver imaging (ultrasound, magnetic resonance, or computerized tomography) to evaluate liver disease;

- Record the appearance or worsening of clinical symptoms of hepatitis, or hypersensitivity, fatigue, decreased appetite, nausea, vomiting, abdominal pain, jaundice, fever, or rash as relevant on the AE report form;
- Record use of concomitant medications, acetaminophen, herbal remedies, other over the counter medications, or putative hepatotoxins, on the concomitant medications report form. Record alcohol use on the liver event alcohol intake case report form.

10.3.2 Allergic Reaction

Subjects may continue DTG for Grade 1 or 2 allergic reactions at the discretion of the Investigator. The subject should be advised to contact the Investigator immediately if there is any worsening of symptoms or if further systemic signs or symptoms develop. Antihistamines, topical corticosteroids, or antipruritic agents may be prescribed.

Subjects with Grade ≥3 allergic reactions that are considered to be possibly or probably related to DTG should permanently discontinue the investigational product regimen and the subject should be withdrawn from the study. Subjects should be treated as clinically appropriate and followed until resolution of the AE.

10.3.3 Rash

Mild to moderate rash is an expected adverse reaction for DTG- containing ART. Episodes generally occur within the first ten weeks of treatment, rarely require interruptions or discontinuations of therapy and tend to resolve within two to three weeks. No instances of serious skin reaction, including Stevens-Johnson Syndrome (SJS), Toxic Epidermal Necrolysis (TEN) and erythema multiforme, have been reported for DTG in clinical trials.

Subjects with an isolated Grade 1 rash may continue DTG at the Investigator’s discretion. The subject should be advised to contact the Investigator immediately if there is any worsening of the rash, if any systemic signs or symptoms worsen, or if mucosal involvement develops. Any patient who presents with a rash should have their liver function tests checked.

Subjects may continue DTG for an isolated Grade 2 rash. However, DTG (and all other concurrent medication(s) suspected in the Investigators causality assessment) should be permanently discontinued for any Grade ≥2 rash that is associated with an increase in ALT. The subject should be advised to contact the physician immediately if rash fails to resolve (after more than two weeks), if there is any worsening of the rash, if any systemic signs or allergic symptoms develop, or if mucosal involvement develops.

Subjects should permanently discontinue DTG (and all other concurrent medication(s) suspected in the Investigators causality assessment) for an isolated Grade 3 or 4 rash, and the subject should be withdrawn from the study. Subjects should be treated as clinically appropriate and followed until resolution of the AE.

The rash and any associated symptoms should be reported as adverse events (see Section 10.1) and appropriate toxicity ratings should be used to grade the events (based on DAIDS toxicity gradings).

If the etiology of the rash can be definitely diagnosed as being unrelated to DTG and due to a specific medical event or a concomitant non-study medication, routine management should be performed and documentation of the diagnosis provided.

10.3.4 Suicidal Risk Monitoring

Subjects with HIV infection may occasionally present with symptoms of depression and/or suicidality (suicidal ideation or behaviour). In addition, there have been some reports of depression, suicidal ideation and behaviour (particularly in patients with a pre-existing history of depression or psychiatric illness) in some patients being treated with integrase inhibitors, including DTG. Therefore, it is appropriate to monitor subjects for suicidality before and during treatment.

Subjects should be monitored appropriately and observed closely for suicidal ideation and behaviour or any other unusual changes in behaviour at all study visits. It is recommended that the investigator consider mental health consultation or referral for subjects who experience signs of suicidal ideation or behaviour. The Edinburgh Postnatal Depression Scale (Appendix 5) will also be administered at the early postnatal visit (see Section 9.2).

If any subject experiences a possible suicidality-related adverse event (PSRAE) while participating in this study that is considered by the Investigator to meet International Conference on Harmonization (ICH)-E2A definitions for seriousness, the Investigator will collect information using a PSRAE CRF form (see Section 10.1).

10.3.5 Decline in Renal Function

Treatment with any fixed dose combination NRTI products containing 3TC, FTC or TDF must be discontinued in any Subject developing moderate to severe renal impairment during the study, as indicated by creatinine clearance measuring <50 mL/min via Cockroft-Gault.

10.4 Safety Reporting

10.4.1 Pregnancy Reporting

Pregnancy complications (e.g., preeclampsia or eclampsia, prolonged hospitalization after delivery, for wound infections etc, seizures) and elective terminations for medical reasons must be reported as an AE (Section 10.1) or SAE (Section 10.2). Spontaneous abortions must be reported as an SAE (Section 10.2).

Additionally, Investigators are requested to register each Maternal subject with the Antiretroviral Pregnancy Registry (APR) prospectively as soon as possible after the subject starts treatment as part of the study on Day 1, and before the pregnancy outcome (otherwise there is less likelihood of the subject being included in the APR). More information including copies of applicable CRFs and fax numbers are available at www.apregistry.com.

10.5 Adverse Event Reporting

All observed or volunteered AEs regardless of treatment group or suspected causal relationship will be reported. For all AEs, the investigator must pursue and obtain information adequate both to determine the outcome of the AE and to assess whether it meets the criteria for classification as an SAE requiring immediate notification.

Each AE is to be assessed to determine if it meets the criteria for SAEs. If an SAE occurs that meets the IRB, local and international regulations for reporting, expedited reporting will be done.. All SAEs will be reported to the sponsor within 24 hours of knowing about the event. Further relevant follow-up information will be given as soon as possible. Follow-up will continue until the event resolves.

All SAEs must be reported immediately. The SAEs should be reported immediately to the Principal Investigator (within 24 hours of a member of the study team becoming aware of the event). A SAE form should be completed, and an assessment of whether the SAE is a Suspected Unexpected Serious Adverse Reaction (SUSAR) conducted. The Principal Investigator is responsible for determining whether the SAE is a Suspected Unexpected Serious Adverse Reaction (SUSAR) and for reporting this in accordance with Good Clinical Practice (GCP) and applicable regulatory requirements. All SAE must be reported in accordance with local protocols for reporting adverse study events.

. All AEs will be reported on the AE page(s) of the CRF. For example, the same AE term should be used on both forms. AEs should be reported using concise medical terminology on the CRFs as well as on the form for collection of SAE information.

10.6 Management of Post-recruitment Illness

All subjects with post-recruitment illness will be monitored until symptoms resolve, laboratory changes return to baseline or until there is a satisfactory explanation for the changes observed. Patients will receive medical care including admission at the hospital associated with the study and patients will be managed in accordance with national treatment guidelines.

11 DATA AND STATISTICAL ANALYSIS

Primary PK endpoints will be estimated using non-compartmental methods (WinNonlin, Pharsight). For study drug concentrations, concentrations will be summarized descriptively (n, mean, SD, CV, median, minimum, maximum, geometric mean and its associated CV and confidence intervals) by day and nominal time. The CIs will be determined using logarithms of the individual geometric mean values; the calculated values will be then expressed as linear values. The changes in PK parameters will be considered significant when the CI do not cross the value 1. Individual subject and median profiles of the concentration-time data will be plotted by dose, cycle and day (single dose and steady-state) using nominal times. Median profiles will be presented on both linear-linear and log-linear scales. The use of mathematical techniques(e.g. non-linear mixed effects) for modelling and simulation will also be explored, and data pooling (subject to quality assessment) with other studies is allowed.

12 STUDY MANAGEMENT

The day-to-day management of the DolPHIN study will be undertaken by the Study Team. A Trial Steering Committee (TSC) will oversee the conduct of the study, and will receive reports from the Study Team, and the IDSMB. The TSC will be able to stop or modify the study, acting on its own judgement or upon any recommendation from the IDSMB.

Thestudy sitewill be responsible for conducting the trial. The study site will collaborate with the affiliated antenatal clinics for this project for patient recruitment and follow up. Safety laboratory tests will be performed at laboratories which have achieved appropriate accreditation by the relevant professional body.

12.1 Liverpool

The Liverpool Bioanalytical Facility (BAF) is GCLP-accredited, and provides mass spectrometry based drug concentration measurement in plasma, CSF, breast milk, genital secretions, tissue and cells. We currently undertake work for large clinical trials consortia (funded by the UK MRC, Wellcome Trust, Gates Foundation, NIHR and other funders) as well as for the pharmaceutical industry. Data analyses are supported by model based (population PK) approaches established through Wellcome Trust Programme Grant funding.

12.2 Sample Processing

Following collection, study bloods will be processed and stored locally at IDI or UCT prior to shipment and analysis at the BAF laboratory. A Material Transfer Agreement between the Infectious Diseases institute or University of Cape Town and the University of Liverpool will be developed prior to commencement of the study.

12.3 Clinical Monitoring

At each study day visit including screening, the patient will be assessed by the research physician or nurse. In addition, patients will be given details of how to reach a member of the study team in an emergency.

12.4 Laboratory Monitoring

Safety bloods (haemoglobin, white cell count, platelets, urea, creatinine, electrolytes, ALT) will be performed at every study visit. Additionally, should an adverse event be reported, further samples will be taken as soon as practically possible. The anti-retroviral efficacy of the novel combination compared with standard of care will be monitored by measurement of CD4 count and HIV viral load at baseline, and delivery. Severity of abnormalities will be defined according to the DAIDS criteria in Appendix 4.

12.5 Data Safety and Monitoring Group

An independent data safety and monitoring group (IDSMB) will be formed and will monitor study data according to the IDSMB charter. The IDSMB is scheduled to meet prior to the enrollment of the first patient, during the scheduled interim analysis (after the first 16 patients have delivered) and upon completion of the study. At each point the IDSMBwill review laboratory and clinical data relating to mother and child. At the interim analysis, the IDSMBwill discuss (in closed session) whether the study should be stopped, continued or modified, and will

convey this recommendation to the Trial Steering Group. Further IDSMB meetings may be convened (at the discretion of the IDSMBchair, or upon request from the Trial Steering Committee) if unexpected data emerge, or following any modification of the study. Any Serious Adverse Events (SAEs) or Suspected Unexpected Serious Adverse Reaction (SUSARs) (see Section 11 of the protocol) will be reported to the IDSMBin a timely manner.

12.6 Termination of the trial

The Sponsor or Investigator may terminate either part of, or the entire study for safety or administrative reasons. A written statement fully documenting the reasons for such a termination will be provided to the Ethics Committee and the Regulatory Authority as appropriate.

13 DATA HANDLING

13.1 Recording of Data

The data collected during the study will be recorded in an individual, subject specific Case Report Form (CRF). In order to maintain confidentiality, the subject will be identified only by subject number and initials on the CRF. All CRFs should be completed legibly by an appropriate member of the study team who should be identified. A CRF must be completed for each subject who signs a consent form and undergoes any screening procedures.

Corrections to the data on the CRF will only be made by drawing a single line through the incorrect data (so as not to obscure the original entry) and inserting the correct data next to the original entry. The incorrect data must never be obliterated using correction fluid. Each correction will be initialed and dated by the person making the correction.

13.2 Source Documentation and Study Records

The subject’s number and date of entry into the study, along with a study identifier, should be recorded in the subject’s study records. The following should also be recorded in the study records; confirmation of written consent, the subject’s clinical status, date of every study visit, date study medication was given, concomitant medications, copies of all relevant reports and laboratory tests, comments on results and reference to any adverse events.

13.3 Data management

Data will be entered into a study specific database by designated staff on a regular basis from completed CRFs, to ensure that it is up to date. The database will be kept on a secure PC. Access to the database will be given to authorised personnel only and a log of authorised personnel will be stored in the trial master file.

Any data anomalies or values found to be outside normal ranges will be checked with the Investigator. When corrections are required these will be made on CRF and the study database will be amended.

Data sharing with other studies is allowed, on discretion of the study team, who will evaluate potential collaborations on a case by case basis, depending on the scientific merit, research

governance, costs and data quality. The guiding principle will always be to maximize the wider benefits of this study wherever possible.

13.4 Archiving and storage of data

Following completion of the study, subject records, CRF and other study documentation will be retained by the Investigator in accordance with Good Clinical Practice and applicable regulatory requirements.

14 QUALITY CONTROL AND QUALITY ASSURANCE

14.1 Monitoring Arrangements

The purpose of monitoring is to verify the rights and well-being of human subjects are protected; that trial data is accurate, complete and verifiable with source data; that the trial is conducted in compliance with the protocol, GCP and the applicable regulatory requirements.

The Sponsor will act as monitor of the study. The Investigator must agree to allow the study monitor to inspect all CRF and corresponding source documents, e.g. original medical records, subject records and laboratory raw data, access to the clinical supplies, dispensing and storage areas and agree to assist with their activities if requested. The Investigator should provide adequate time and space for monitoring visits.

The monitor will query any missing or spurious data with the Investigator, which should be resolved in a timely manner. A monitoring log will be maintained recording each visit, the reason for the visit, the monitor's signature and Investigator's or designee's confirmation signature.

14.2 Quality Assurance

For the purpose of compliance with GCP and regulatory agency guidelines, it may be necessary for sponsor authorised Quality Assurance personnel and/or authorised personnel from an external regulatory agency to conduct an audit/inspection of an Investigational site. The purpose of an audit is to assess the quality of data with regard to accuracy, adequacy and consistency, and to assure that studies are in accordance with GCP and Regulatory Agency guidelines. Having the highest quality data from studies is an essential aspect of drug development.

The Investigator will be given sufficient notice to prepare for such visits, which are planned to take usually between one and two days and may be conducted at any stage during the study. The audit will involve the review of all study related documentation, which is required by GCP to be maintained by each site, review of drug storage, dispensing and return, review of all study related supplies and review of source documents against the CRF to assure the adequacy and accuracy of the information which has been recorded, including the verification of any AE which have occurred.

15 ETHICS & ADMINISTRATION

15.1 Ethics Approval

The study protocol, subject information and consent form, available safety information, subject recruitment procedures (e.g. advertisements), information about payments and compensation available to the subjects and documentation evidencing the Investigator's qualifications will be submitted to the Ethics Committee for ethical review and approval according to local regulations,

prior to the study start. Any changes, which may need to be made, will be submitted in the form of numbered and dated protocol amendments in accordance with local regulations.

15.2 Regulatory Approval

As required by local regulations, approval of the appropriate regulatory bodies will be obtained, prior to study initiation.

15.3 Publication Policy

A whole or part of this study results will be communicated, orally presented, and/or published in appropriate scientific journals. Full anonymity of subject's details will be maintained throughout. All communications will be available on our website www.hiv-druginteractions.org.

15.4 Drug Accountability

The investigator will ensure that the IP will only be used in accordance with the protocol. Drug supplies will be kept in a secure, limited access storage area under the recommended storage conditions. A drug accountability log will be kept with the investigational supplies for reconciliation purposes. This should be used to record the identification of the subject to whom the IP was dispensed, the date and quantity of IP dispensed. This will be verified by the study monitor.

15.5 Financial Aspects

This trial is being funded by ViiV. The University of Liverpool will administer the funds, act as Sponsor for this study, and provide appropriate insurance in accordance with current arrangements for clinical trials conducted in sub-Saharan Africa.

16 REFERENCES

(2011). Malawi Integrated Guidelines for Providing HIV Services. Lilongwe, Ministry of Health, Malawi.

Cohen, K. and G. Meintjes (2010). "Management of individuals requiring antiretroviral therapy and TB treatment." Curr Opin HIV AIDS **5**(1): 61-69.

Dooley, K. E., P. Sayre, et al. (2013). "Safety, tolerability, and pharmacokinetics of the HIV integrase inhibitor dolutegravir given twice daily with rifampin or once daily with rifabutin: results of a phase 1 study among healthy subjects." J Acquir Immune Defic Syndr **62**(1): 21-27.

Flys, T., D. V. Nissley, et al. (2005). "Sensitive drug-resistance assays reveal long-term persistence of HIV-1 variants with the K103N nevirapine (NVP) resistance mutation in some women and infants after the administration of single-dose NVP: HIVNET 012." J Infect Dis **192**(1): 24-29.

Hamers, R. L., M. Siwale, et al. (2010). "HIV-1 drug resistance mutations are present in six percent of persons initiating antiretroviral therapy in Lusaka, Zambia." J Acquir Immune Defic Syndr **55**(1): 95-101.

Hedt, B. L., N. Wadonda-Kabondo, et al. (2008). "Early warning indicators for HIV drug resistance in Malawi." Antivir Ther **13 Suppl 2**: 69-75.

Jacob, M. S., A. Durairaj, et al. (2011). "Detection of HIV drug resistance mutations in pregnant women receiving single dose Nevirapine in south India." Indian J Pathol Microbiol **54**(2): 359-361.

Koole, O. and R. Colebunders (2011). "Reducing mortality from HIV infection and tuberculosis." Lancet Infect Dis **11**(7): 494-495.

Lawn, S. D., L. Myer, et al. (2006). "Burden of tuberculosis in an antiretroviral treatment programme in sub-Saharan Africa: impact on treatment outcomes and implications for tuberculosis control." AIDS**20**(12): 1605-1612.

Manasa, J., D. Katzenstein, et al. (2012). "Primary drug resistance in South Africa: data from 10 years of surveys." AIDS Res Hum Retroviruses**28**(6): 558-565.

Messiaen, P., A. M. Wensing, et al. (2013). "Clinical Use of HIV Integrase Inhibitors: A Systematic Review and Meta-Analysis." PLoS One**8**(1): e52562.

Min, S., L. Sloan, et al. (2011). "Antiviral activity, safety, and pharmacokinetics/pharmacodynamics of dolutegravir as 10-day monotherapy in HIV-1-infected adults." AIDS**25**(14): 1737-1745.

Musiime, V., F. Ssali, et al. (2009). "Response to nonnucleoside reverse transcriptase inhibitor-based therapy in HIV-infected children with perinatal exposure to single-dose nevirapine." AIDS Res Hum Retroviruses**25**(10): 989-996.

Niemi, M., J. T. Backman, et al. (2003). "Pharmacokinetic interactions with rifampicin : clinical relevance." Clin Pharmacokinet**42**(9): 819-850.

Rockstroh, J. K., E. Dejesus, et al. (2013). "Durable Efficacy and Safety of Raltegravir versus Efavirenz When Combined With Tenofovir/Emtricitabine In Treatment-Naive HIV-1 Infected Patients: Final Five-Year Results From STARTMRK." J Acquir Immune Defic Syndr.

Sax, P. E., E. DeJesus, et al. (2012). "Co-formulated elvitegravir, cobicistat, emtricitabine, and tenofovir versus co-formulated efavirenz, emtricitabine, and tenofovir for initial treatment of HIV-1 infection: a randomised, double-blind, phase 3 trial, analysis of results after 48 weeks." Lancet**379**(9835): 2439-2448.

Teasdale CA, Marais BJ, Abrams EJ. HIV: prevention of mother-to-child transmission. Clin Evid (Online). 2011 Jan 17;2011

van Lunzen, J., F. Maggiolo, et al. (2012). "Once daily dolutegravir (S/GSK1349572) in combination therapy in antiretroviral-naive adults with HIV: planned interim 48 week results from SPRING-1, a dose-ranging, randomised, phase 2b trial." Lancet Infect Dis**12**(2): 111-118.

Wadonda-Kabondo, N., R. Banda, et al. (2012). "Prevalence of transmitted HIV drug resistance among newly diagnosed antiretroviral therapy-naive pregnant women in Lilongwe and Blantyre, Malawi." Clin Infect Dis**54 Suppl 4**: S324-327.

Wagner, T. A., C. M. Kress, et al. (2010). "Detection of HIV-1 drug resistance in women following administration of a single dose of nevirapine: comparison of plasma RNA to cellular DNA by consensus sequencing and by oligonucleotide ligation assay." J Clin Microbiol**48**(5): 1555-1561.

Walmsley, S., A. Antela, et al. (2012). Dolutegravir (DTG; S/GSK 1349572) + Abacavir/Lamivudine once daily statistically superior to Tenofovir/Emtricitabine/Efavirenz: 48-week results - SINGLE (ING114467). ICAAC. Glasgow.

WHO (2010). Antiretroviral Therapy for HIV Infection in Adults and Adolescents: Recommendations for a Public Health Approach. 2010 Revision. WHO. Geneva, World Health Organisation.

WHO (2012). Programmatic Update: Use of Antiretroviral Drugs for Treating Pregnant Women and Preventing HIV Infection in Infants, World Health Organisation.

WHO (2012). WHO HIV drug resistance report 2012. Geneva, WHO.

Zeh, C., P. J. Weidle, et al. (2011). "HIV-1 drug resistance emergence among breastfeeding infants born to HIV-infected mothers during a single-arm trial of triple-antiretroviral prophylaxis for prevention of mother-to-child transmission: a secondary analysis." PLoS Med**8**(3): e1000430.

17 PROTOCOL SIGNATURE PAGE

I agree to conduct the trial in accordance with ICH-GCP and the applicable regulatory requirements and with the approved protocol.

I agree to comply with the procedures for data recording/reporting

I agree to permit monitoring, auditing and inspection and to retain the trial related essential documentation for the period of time required according to ICH-GCP.

Name of Chief Investigator: Professor Saye Khoo

Signature:

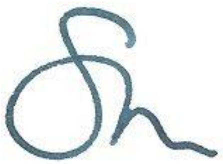

Date: 10th June 2016

18 APPENDIX 1 SAFETY QUESTIONNAIRE

a) Maternal Safety Questionnaire

1) Since your last clinic visit have you had any new symptoms? Y/N

2) If Y, which of the following:

   Nausea, vomiting, diarrhea

   Fever, chills, sweating

   Skin rash, itch

   Chest pain, breathlessness, fast heart beat

   Headache, drowsiness, visual disturbance,

   Dizziness, poor sleep, low mood

   Eyes going yellow, dark urine

   Change in bowel habit

   Other (state): ___________________________

3) Have you taken your medication as prescribed? Y/N
4) If not, why not? ____
5) Have you taken any new medication since your last visit? Y/N
6) If Y, which? ____
7) Have you any other new concerns about your health? __________________________

b) Infant Safety Questionnaire

1) Have you any concerns about your infant's health? Y/N

2) If Y, which of the following?

- Poor feeding
- Irritability, lethargy
- Difficulty breathing
- Skin rash
- Fever, chills
- Eyes going yellow, or dark urine
- Loose motions Diarrhea?
- Other (state): ______________________________

19 APPENDIX 2 DAIDS GRADING SCALE

Division of Aids Table for Grading the Severity of Adult and Paediatric Adverse Events (2004)

Note: as the inclusion criteria for this trial is people ≥18 years old, the paediatric (<18years) AE's were removed from the appendices.

The Division of AIDS Table for Grading the Severity of Adult and Paediatric Adverse Events ("DAIDS AE grading table") is a descriptive terminology which can be utilized for Adverse Event (AE) reporting. A grading (severity) scale is provided for each AE term.

General Instructions

Estimating Severity Grade

If the need arises to grade a clinical AE that is not identified in the DAIDS AE grading table, use the category "Estimating Severity Grade". For AEs that are not listed in the table but will be collected systematically for a study/trial, protocol teams are highly encouraged to define study specific severity scales within the protocol or an appendix to the protocol. (Please see "Template Wording for the Expedited Adverse Event Reporting Section of DAIDS-sponsored Protocols".) This is particularly important for laboratory values because the "Estimating Severity Grade" category only applies to clinical symptoms.

Grading Adult and Paediatric AEs

The DAIDS AE grading table includes parameters for grading both Adult and Paediatric AEs. When a single set of parameters is not appropriate for grading specific types of AEs for both Adult and Paediatric populations, separate sets of parameters for Adult and/or Paediatric populations (with specified respective age ranges) are given in the table. If there is no distinction in the table between Adult and Paediatric values for a type of AE, then the single set of parameters listed is to be used for grading the severity of both Adult and Paediatric events of that type.

Determining Severity Grade

If the severity of an AE could fall under either one of two grades (e.g., the severity of an AE could be either Grade 2 or Grade 3), select the higher of the two grades for the AE.

Definitions

Basic Self-care Functions	Adult<br>Activities such as bathing, dressing, toileting, transfer/movement, continence, and feeding.<br>Young Children<br>Activities that are age and culturally appropriate (e.g., feeding self with culturally appropriate eating implement).
LLN	Lower limit of normal
Medical Intervention	Use of pharmacologic or biologic agent(s) for treatment of an AE.
NA	Not Applicable
Operative Intervention	Surgical OR other invasive mechanical procedures.
ULN	Upper limit of normal
Usual Social & Functional Activities	Adult<br>Adaptive tasks and desirable activities, such as going to work, shopping, cooking, use of transportation, pursuing a hobby, etc.<br>Young Children<br>Activities that are age and culturally appropriate (e.g., social interactions, play activities, learning tasks, etc.).

CLINICAL				
**PARAMETER**	**GRADE 1**<br><br>**MILD**	**GRADE 2**<br><br>**MODERATE**	**GRADE 3**<br><br>**SEVERE**	**GRADE 4**<br><br>**POTENTIALLY LIFE-THREATENING**
**ESTIMATING SEVERITY GRADE**				
Clinical adverse event NOT identified elsewhere in this DAIDS AE grading table	Symptoms causing no or minimal interference with usual social & functional activities	Symptoms causing greater than minimal interference with usual social & functional activities	Symptoms causing inability to perform usual social & functional activities	Symptoms causing inability to perform basic self-care functions OR Medical or operative intervention indicated to prevent permanent impairment, persistent disability, or death
**SYSTEMIC**				
Acute systemic allergic reaction	Localized urticaria (wheals) with no medical intervention indicated	Localized urticaria with medical intervention indicated OR Mild angioedema with no medical intervention indicated	Generalized urticaria OR Angioedema with medical intervention indicated OR Symptomatic mild bronchospasm	Acute anaphylaxis OR Life-threatening bronchospasm OR laryngeal edema
Chills	Symptoms causing no or minimal interference with usual social & functional activities	Symptoms causing greater than minimal interference with usual social & functional activities	Symptoms causing inability to perform usual social & functional activities	NA
Fatigue<br><br>Malaise	Symptoms causing no or minimal interference with usual social & functional activities	Symptoms causing greater than minimal interference with usual social & functional activities	Symptoms causing inability to perform usual social & functional activities	Incapacitating fatigue/ malaise symptoms causing inability to perform basic self-care functions
Fever (nonaxillary)	37.7 – 38.6°C	38.7 – 39.3°C	39.4 – 40.5°C	> 40.5°C

Pain (indicate body site)<br><br>DO NOT use for pain due to injection (See Injection Site Reactions: Injection site pain)<br><br>See also Headache, Arthralgia, and Myalgia	Pain causing no or minimal interference with usual social & functional activities	Pain causing greater than minimal interference with usual social & functional activities	Pain causing inability to perform usual social & functional activities	Disabling pain causing inability to perform basic self-care functions OR Hospitalization (other than emergency room visit) indicated
Unintentional weight loss	NA	5 – 9% loss in body weight from baseline	10 – 19% loss in body weight from baseline	≥ 20% loss in body weight from baseline OR Aggressive intervention indicated [e.g., tube feeding or total parenteral nutrition (TPN)]
**INFECTION**				
Infection (any other than HIV infection)	Localized, no systemic antimicrobial treatment indicated AND Symptoms causing no or minimal interference with usual social & functional activities	Systemic antimicrobial treatment indicated OR Symptoms causing greater than minimal interference with usual social & functional activities	Systemic antimicrobial treatment indicated AND Symptoms causing inability to perform usual social & functional activities OR Operative intervention (other than simple incision and drainage) indicated	Life-threatening consequences (e.g., septic shock)
**INJECTION SITE REACTIONS**				
Injection site pain (pain without touching) Or Tenderness (pain when area is touched)	Pain/tenderness causing no or minimal limitation of use of limb	Pain/tenderness limiting use of limb OR Pain/tenderness causing greater than minimal interference with usual social & functional activities	Pain/tenderness causing inability to perform usual social & functional activities	Pain/tenderness causing inability to perform basic self-care function OR Hospitalization (other than emergency room visit) indicated for management of pain/tenderness
Injection site reaction (localized)				
Adult > 15 years	Erythema OR Induration of 5x5 cm – 9x9 cm (or 25 cm2 – 81cm2)	Erythema OR Induration OR Edema > 9 cm any diameter (or > 81 cm2)	Ulceration OR Secondary infection OR Phlebitis OR Sterile abscess OR Drainage	Necrosis (involving dermis and deeper tissue)

Pruritis associated with injection<br><br>See also Skin: Pruritis (itching - no skin lesions)	Itching localized to injection site AND Relieved spontaneously or with < 48 hours treatment	Itching beyond the injection site but not generalized OR Itching localized to injection site requiring ≥ 48 hours treatment	Generalized itching causing inability to perform usual social & functional activities	NA
**SKIN – DERMATOLOGICAL**				
Alopecia	Thinning detectable by study participant (or by caregiver for young children and disabled adults)	Thinning or patchy hair loss detectable by health care provider	Complete hair loss	NA
Cutaneous reaction – rash	Localized macular<br><br>rash	Diffuse macular, maculopapular, or morbilliform rash OR Target lesions	Diffuse macular, maculopapular, or morbilliform rash with vesicles or limited number of bullae OR Superficial ulcerations of mucous membrane limited to one site	Extensive or generalized bullous lesions OR Stevens-Johnson syndrome OR Ulceration of mucous membrane involving two or more distinct mucosal sites OR Toxic epidermal necrolysis (TEN)
Hyperpigmentation	Slight or localized	Marked or generalized	NA	NA
Hypopigmentation	Slight or localized	Marked or generalized	NA	NA
Pruritis (itching – no skin lesions)<br><br>(See also Injection Site Reactions: Pruritis associated with injection)	Itching causing no or minimal interference with usual social & functional activities	Itching causing greater than minimal interference with usual social & functional activities	Itching causing inability to perform usual social & functional activities	NA
**CARDIOVASCULAR**				
Cardiac arrhythmia (general)<br><br>(By ECG or physical exam)	Asymptomatic AND No intervention indicated	Asymptomatic AND Non-urgent medical intervention indicated	Symptomatic, non-life threatening AND Non urgent medical intervention indicated	Life-threatening arrhythmia OR Urgent intervention indicated

Cardiac ischemia/ infarction	NA	NA	Symptomatic ischemia (stable angina) OR Testing consistent with ischemia	Unstable angina OR Acute myocardial infarction
Haemorrhage (significant acute blood loss)	NA	Symptomatic AND No transfusion indicated	Symptomatic AND Transfusion of ≤ 2 units packed RBCs (for children ≤ 10 cc/kg) indicated	Life-threatening hypotension OR Transfusion of > 2 units packed RBCs (for children > 10 cc/kg) indicated
Hypertension				
Adult > 17 years (with repeat testing at same visit)	> 140 – 159 mmHg systolic OR > 90 – 99 mmHg diastolic	> 160 – 179 mmHg systolic OR > 100 – 109 mmHg diastolic	> 180 mmHg systolic OR > 110 mmHg diastolic	Life-threatening consequences (e.g., malignant hypertension) OR Hospitalization indicated (other than emergency room visit)
Hypotension	NA	Symptomatic, corrected with oral fluid replacement	Symptomatic, IV fluids indicated	Shock requiring use of vasopressors or mechanical assistance to maintain blood pressure
Pericardial effusion	Asymptomatic, small effusion requiring no intervention	Asymptomatic, moderate or larger effusion requiring no intervention	Effusion with non-life threatening physiologic consequences OR Effusion with non-urgent intervention indicated	Life-threatening consequences (e.g., tamponade) OR Urgent intervention indicated
Prolonged PR interval				
Adult > 16 years	PR interval 0.21 – 0.25 sec	PR interval > 0.25 sec	Type II 2nd degree AV block OR Ventricular pause > 3.0 sec	Complete AV block
Prolonged QTc				
Adult > 16 years	Asymptomatic, QTc interval 0.45 – 0.47 sec OR Increase interval < 0.03 sec above baseline	Asymptomatic, QTc interval 0.48 – 0.49 sec OR Increase in interval 0.03 – 0.05 sec above baseline	Asymptomatic, QTc interval ≥ 0.50 sec OR Increase in interval ≥ 0.06 sec above baseline	Life-threatening consequences, e.g. Torsade de pointes or other associated serious ventricular dysrhythmia

Thrombosis/embolism	NA	Deep vein thrombosis AND No intervention indicated (e.g., anticoagulation, lysis filter, invasive procedure)	Deep vein thrombosis AND Intervention indicated (e.g., anticoagulation, lysis filter, invasive procedure)	Embolic event (e.g., pulmonary embolism, life-threatening thrombus)
Vasovagal episode (associated with a procedure of any kind)	Present without loss of consciousness	Present with transient loss of consciousness	NA	NA
Ventricular dysfunction (congestive heart failure)	NA	Asymptomatic diagnostic finding AND intervention indicated	New onset with symptoms OR Worsening symptomatic congestive heart failure	Life-threatening congestive heart failure
**GASTROINTESTINAL**				
Anorexia	Loss of appetite without decreased oral intake	Loss of appetite associated with decreased oral intake without significant weight loss	Loss of appetite associated with significant weight loss	Life-threatening consequences OR Aggressive intervention indicated [e.g., tube feeding or total parenteral nutrition (TPN)]
Ascites	Asymptomatic	Symptomatic AND Intervention indicated (e.g., diuretics or therapeutic paracentesis)	Symptomatic despite intervention	Life-threatening consequences
Cholecystitis	NA	Symptomatic AND Medical intervention indicated	Radiologic, endoscopic, or operative intervention indicated	Life-threatening consequences (e.g., sepsis or perforation)
Constipation	NA	Persistent constipation requiring regular use of dietary modifications, laxatives, or enemas	Constipation with manual evacuation indicated	Life-threatening consequences (e.g., obstruction)
Diarrhoea				

Adult and Paediatric ≥1 year	Transient or intermittent episodes of unformed stools OR Increase of ≤ 3 stools over baseline per 24-hour period	Persistent episodes of unformed to watery stools OR Increase of 4 – 6 stools over baseline per 24-hour period	Bloody diarrhoea OR Increase of ≥ 7 stools per 24-hour period OR IV fluid replacement indicated	Life-threatening consequences (e.g., hypotensive shock)
Dysphagia-<br>Odynophagia	Symptomatic but able to eat usual diet	Symptoms causing altered dietary intake without medical intervention indicated	Symptoms causing severely altered dietary intake with medical intervention indicated	Life-threatening reduction in oral intake
Mucositis/stomatitis (clinical exam) Indicate site (e.g., larynx, oral)<br>See Genitourinary for Vulvovaginitis<br>See also Dysphagia-Odynophagia and Proctitis	Erythema of the mucosa	Patchy pseudomembranes or ulcerations	Confluent pseudomembranes or ulcerations OR Mucosal bleeding with minor trauma	Tissue necrosis OR Diffuse spontaneous mucosal bleeding OR Life-threatening consequences (e.g., aspiration, choking)
Nausea	Transient (< 24 hours) or intermittent nausea with no or minimal interference with oral intake	Persistent nausea resulting in decreased oral intake for 24 – 48 hours	Persistent nausea resulting in minimal oral intake for > 48 hours OR Aggressive rehydration indicated (e.g., IV fluids)	Life-threatening consequences (e.g., hypotensive shock)
Pancreatitis	NA	Symptomatic AND Hospitalization not indicated (other than emergency room visit)	Symptomatic AND Hospitalization indicated (other than emergency room visit)	Life-threatening consequences (e.g., circulatory failure, haemorrhage, sepsis)
Proctitis (functional-symptomatic)<br>Also see Mucositis/stomatitis for clinical exam	Rectal discomfort AND No intervention indicated	Symptoms causing greater than minimal interference with usual social & functional activities OR Medical intervention indicated	Symptoms causing inability to perform usual social & functional activities OR Operative intervention indicated	Life-threatening consequences (e.g., perforation)
Vomiting	Transient or intermittent vomiting with no or minimal interference with oral intake	Frequent episodes of vomiting with no or mild dehydration	Persistent vomiting resulting in orthostatic hypotension OR Aggressive rehydration indicated (e.g., IV fluids)	Life-threatening consequences (e.g., hypotensive shock)

NEUROLOGIC				
Alteration in personality-behaviour or in mood (e.g., agitation, anxiety, depression, mania, psychosis)	Alteration causing no or minimal interference with usual social & functional activities	Alteration causing greater than minimal interference with usual social & functional activities	Alteration causing inability to perform usual social & functional activities	Behaviour potentially harmful to self or others (e.g., suicidal and homicidal ideation or attempt, acute psychosis) OR Causing inability to perform basic self-care functions
Altered Mental Status<br><br>For Dementia, see Cognitive and behavioural/attentional disturbance (including dementia and attention deficit disorder)	Changes causing no or minimal interference with usual social & functional activities	Mild lethargy or somnolence causing greater than minimal interference with usual social & functional activities	Confusion, memory impairment, lethargy, or somnolence causing inability to perform usual social & functional activities	Delirium OR obtundation, OR coma
Ataxia	Asymptomatic ataxia detectable on exam OR Minimal ataxia causing no or minimal interference with usual social & functional activities	Symptomatic ataxia causing greater than minimal interference with usual social & functional activities	Symptomatic ataxia causing inability to perform usual social & functional activities	Disabling ataxia causing inability to perform basic self-care functions
Cognitive and behavioural/attentional disturbance (including dementia and attention deficit disorder)	Disability causing no or minimal interference with usual social & functional activities OR Specialized resources not indicated	Disability causing greater than minimal interference with usual social & functional activities OR Specialized resources on part-time basis indicated	Disability causing inability to perform usual social & functional activities OR Specialized resources on a full-time basis indicated	Disability causing inability to perform basic self-care functions OR Institutionalization indicated
CNS ischemia (acute)	NA	NA	Transient ischemic attack	Cerebral vascular accident (CVA, stroke) with neurological deficit

Developmental delay – **Paediatric ≤16 years**	Mild developmental delay, either motor or cognitive, as determined by comparison with a developmental screening tool appropriate for the setting	Moderate developmental delay, either motor or cognitive, as determined by comparison with a developmental screening tool appropriate for the setting	Severe developmental delay, either motor or cognitive, as determined by comparison with a developmental screening tool appropriate for the setting	Developmental regression, either motor or cognitive, as determined by comparison with a developmental screening tool appropriate for the setting
Headache	Symptoms causing no or minimal interference with usual social & functional activities	Symptoms causing greater than minimal interference with usual social & functional activities	Symptoms causing inability to perform usual social & functional activities	Symptoms causing inability to perform basic self-care functions OR Hospitalization indicated (other than emergency room visit) OR Headache with significant impairment of alertness or other neurologic function
Insomnia	NA	Difficulty sleeping causing greater than minimal interference with usual social & functional activities	Difficulty sleeping causing inability to perform usual social & functional activities	Disabling insomnia causing inability to perform basic self-care functions
Neuromuscular weakness (including myopathy & neuropathy)	Asymptomatic with decreased strength on exam OR Minimal muscle weakness causing no or minimal interference with usual social & functional activities	Muscle weakness causing greater than minimal interference with usual social & functional activities	Muscle weakness causing inability to perform usual social & functional activities	Disabling muscle weakness causing inability to perform basic self-care functions OR Respiratory muscle weakness impairing ventilation
Neurosensory alteration (including paresthesia and painful neuropathy)	Asymptomatic with sensory alteration on exam or minimal paresthesia causing no or minimal interference with usual social & functional activities	Sensory alteration or paresthesia causing greater than minimal interference with usual social & functional activities	Sensory alteration or paresthesia causing inability to perform usual social & functional activities	Disabling sensory alteration or paresthesia causing inability to perform basic self-care functions

Seizure: (new onset) – **Adult ≥ 18 years**<br><br>See also Seizure: (known pre-existing seizure disorder)	NA	1 seizure	2 – 4 seizures	Seizures of any kind which are prolonged, repetitive (e.g., status epilepticus), or difficult to control (e.g., refractory epilepsy)
Seizure: (known pre-existing seizure disorder) **– Adult ≥ 18 years**<br><br>For worsening of existing epilepsy the grades should be based on an increase from previous level of control to any of these levels.	NA	Increased frequency of pre-existing seizures (non-repetitive) without change in seizure character OR Infrequent breakthrough seizures while on stable medication in a previously controlled seizure disorder	Change in seizure character from baseline either in duration or quality (e.g., severity or focality)	Seizures of any kind which are prolonged, repetitive (e.g., status epilepticus), or difficult to control (e.g., refractory epilepsy)
Seizure **– Paediatric < 18 years**	Seizure, generalized onset with or without secondary generalization, lasting < 5 minutes with < 24 hours post ictal state	Seizure, generalized onset with or without secondary generalization, lasting 5 – 20 minutes with < 24 hours post ictal state	Seizure, generalized onset with or without secondary generalization, lasting > 20 minutes	Seizure, generalized onset with or without secondary generalization, requiring intubation and sedation
Syncope (not associated with a procedure)	NA	Present	NA	NA
Vertigo	Vertigo causing no or minimal interference with usual social & functional activities	Vertigo causing greater than minimal interference with usual social & functional activities	Vertigo causing inability to perform usual social & functional activities	Disabling vertigo causing inability to perform basic self-care functions
**RESPIRATORY**				
Bronchospasm (acute)	FEV1 or peak flow reduced to 70 – 80%	FEV1 or peak flow 50 – 69%	FEV1 or peak flow 25 – 49%	Cyanosis OR FEV1 or peak flow < 25% OR Intubation
Dyspnea or respiratory distress				

**Adult ≥ 14 years**	Dyspnea on exertion with no or minimal interference with usual social & functional activities	Dyspnea on exertion causing greater than minimal interference with usual social & functional activities	Dyspnea at rest causing inability to perform usual social & functional activities	Respiratory failure with ventilatory support indicated
**MUSCULOSKELETAL**				
Arthralgia<br><br>See also Arthritis	Joint pain causing no or minimal interference with usual social & functional activities	Joint pain causing greater than minimal interference with usual social & functional activities	Joint pain causing inability to perform usual social & functional activities	Disabling joint pain causing inability to perform basic self-care functions
Arthritis<br><br>See also Arthralgia	Stiffness or joint swelling causing no or minimal interference with usual social & functional activities	Stiffness or joint swelling causing greater than minimal interference with usual social & functional activities	Stiffness or joint swelling causing inability to perform usual social & functional activities	Disabling joint stiffness or swelling causing inability to perform basic self-care functions
Bone Mineral Loss				
**Adult ≥ 21 years**	BMD t-score -2.5 to -1.0	BMD t-score < -2.5	Pathological fracture (including loss of vertebral height)	Pathologic fracture causing life-threatening consequences
**Paediatric < 21 years**	BMD z-score -2.5 to -1.0	BMD z-score < -2.5	Pathological fracture (including loss of vertebral height)	Pathologic fracture causing life-threatening consequences
Myalgia (non-injection site)	Muscle pain causing no or minimal interference with usual social & functional activities	Muscle pain causing greater than minimal interference with usual social & functional activities	Muscle pain causing inability to perform usual social & functional activities	Disabling muscle pain causing inability to perform basic self-care functions
Osteonecrosis	NA	Asymptomatic with radiographic findings AND No operative intervention indicated	Symptomatic bone pain with radiographic findings OR Operative intervention indicated	Disabling bone pain with radiographic findings causing inability to perform basic self-care functions
**GENITOURINARY**				

Cervicitis (symptoms) (For use in studies evaluating topical study agents)<br><br>For other cervicitis see Infection: Infection (any other than HIV infection)	Symptoms causing no or minimal interference with usual social & functional activities	Symptoms causing greater than minimal interference with usual social & functional activities	Symptoms causing inability to perform usual social & functional activities	Symptoms causing inability to perform basic self-care functions
Cervicitis (clinical exam)<br><br>(For use in studies evaluating topical study agents)<br><br>For other cervicitis see Infection: Infection (any other than HIV infection)	Minimal cervical abnormalities on examination (erythema, mucopurulent discharge, or friability) OR Epithelial disruption < 25% of total surface	Moderate cervical abnormalities on examination (erythema, mucopurulent discharge, or friability) OR Epithelial disruption of 25 – 49% total surface	Severe cervical abnormalities on examination (erythema, mucopurulent discharge, or friability) OR Epithelial disruption 50 – 75% total surface	Epithelial disruption > 75% total surface
Inter-menstrual bleeding (IMB)	Spotting observed by participant OR Minimal blood observed during clinical or colposcopic examination	Inter-menstrual bleeding not greater in duration or amount than usual menstrual cycle	Inter-menstrual bleeding greater in duration or amount than usual menstrual cycle	Haemorrhage with life threatening hypotension OR Operative intervention indicated
Urinary tract obstruction (e.g., stone)	NA	Signs or symptoms of urinary tract obstruction without hydronephrosis or renal dysfunction	Signs or symptoms of urinary tract obstruction with hydronephrosis or renal dysfunction	Obstruction causing life threatening consequences
Vulvovaginitis (symptoms)<br><br>(Use in studies evaluating topical study agents)<br><br>For other vulvovaginitis see Infection: Infection (any other than HIV infection)	Symptoms causing no or minimal interference with usual social & functional activities	Symptoms causing greater than minimal interference with usual social & functional activities	Symptoms causing inability to perform usual social & functional activities	Symptoms causing inability to perform basic self-care functions

Vulvovaginitis (clinical exam)<br><br>(Use in studies evaluating topical study agents)<br><br>For other vulvovaginitis see Infection: Infection (any other than HIV infection)	Minimal vaginal abnormalities on examination OR Epithelial disruption < 25% of total surface	Moderate vaginal abnormalities on examination OR Epithelial disruption of 25 - 49% total surface	Severe vaginal abnormalities on examination OR Epithelial disruption 50 - 75% total surface	Vaginal perforation OR Epithelial disruption > 75% total surface
**OCULAR/VISUAL**				
Uveitis	Asymptomatic but detectable on exam	Symptomatic anterior uveitis OR Medical intervention indicated	Posterior or pan-uveitis OR Operative intervention indicated	Disabling visual loss in affected eye(s)
Visual changes (from baseline)	Visual changes causing no or minimal interference with usual social & functional activities	Visual changes causing greater than minimal interference with usual social & functional activities	Visual changes causing inability to perform usual social & functional activities	Disabling visual loss in affected eye(s)
**ENDOCRINE/METABOLIC**				
Abnormal fat accumulation (e.g., back of neck, breasts, abdomen)	Detectable by study participant (or by caregiver for young children and disabled adults)	Detectable on physical exam by health care provider	Disfiguring OR Obvious changes on casual visual inspection	NA
Diabetes mellitus	NA	New onset without need to initiate medication OR Modification of current medications to regain glucose control	New onset with initiation of medication indicated OR Diabetes uncontrolled despite treatment modification	Life-threatening consequences (e.g., ketoacidosis, hyperosmolar nonketotic coma)
Gynecomastia	Detectable by study participant or caregiver (for young children and disabled adults)	Detectable on physical exam by health care provider	Disfiguring OR Obvious on casual visual inspection	NA

Hyperthyroidism	Asymptomatic	Symptomatic causing greater than minimal interference with usual social & functional activities OR Thyroid suppression therapy indicated	Symptoms causing inability to perform usual social & functional activities OR Uncontrolled despite treatment modification	Life-threatening consequences (e.g., thyroid storm)
Hypothyroidism	Asymptomatic	Symptomatic causing greater than minimal interference with usual social & functional activities OR Thyroid replacement therapy indicated	Symptoms causing inability to perform usual social & functional activities OR Uncontrolled despite treatment modification	Life-threatening consequences (e.g., myxedema coma)
Lipoatrophy (e.g., fat loss from the face, extremities, buttocks)	Detectable by study participant (or by caregiver for young children and disabled adults)	Detectable on physical exam by health care provider	Disfiguring OR Obvious on casual visual inspection	NA

**LABORATORY**				
**PARAMETER**	**GRADE 1 MILD**	**GRADE 2**<br>**MODERATE**	**GRADE 3**<br>**SEVERE**	**GRADE 4**<br>**POTENTIALLY**<br>**LIFE-THREATENING**
**HEMATOLOGY** ***Standard International Units are listed in italics***				
Absolute CD4+ count **– Adult and Paediatric > 13 years** (HIV NEGATIVE ONLY)	300 – 400/mm3<br>*300 – 400/µL*	200 – 299/mm3<br>*200 – 299/µL*	100 – 199/mm3<br>*100 – 199/µL*	< 100/mm3<br>*< 100/µL*
Absolute lymphocyte count **– Adult and Paediatric > 13 years** (HIV NEGATIVE ONLY)	600 – 650/mm3<br>*0.600 x 109 –*<br>*0.650 x 109/L*	500 – 599/mm3<br>*0.500 x 109 –*<br>*0.599 x 109/L*	350 – 499/mm3<br>*0.350 x 109 –*<br>*0.499 x 109/L*	< 350/mm3<br>*< 0.350 x 109/L*
Absolute neutrophil count (ANC)				
**Adult and Paediatric, > 7 days**	1,000 – 1,300/mm3<br>*1.000 x 109 –*<br>*1.300 x 109/L*	750 – 999/mm3<br>*0.750 x 109 –*<br>*0.999 x 109/L*	500 – 749/mm3<br>*0.500 x 109 –*<br>*0.749 x 109/L*	< 500/mm3<br>*<0.500 x 109/L*
Fibrinogen, decreased	100 – 200 mg/dL<br>*1.00 – 2.00 g/L*<br>OR<br>0.75 – 0.99 x LLN	75 – 99 mg/dL<br>*0.75 – 0.99 g/L*<br>OR<br>0.50 – 0.74 x LLN	50 – 74 mg/dL<br>*0.50 – 0.74 g/L*<br>OR<br>0.25 – 0.49 x LLN	< 50 mg/dL<br>*< 0.50 g/L* OR<br>< 0.25 x LLN OR<br>Associated with gross bleeding
Haemoglobin (Hgb)				
**Adult and Paediatric ≥ 57 days** (HIV POSITIVE ONLY)	8.5 – 10.0 g/dL<br>*1.32 – 1.55 mmol/L*	7.5 – 8.4 g/dL<br>*1.16 – 1.31 mmol/L*	6.50 – 7.4 g/dL<br>*1.01 – 1.15 mmol/L*	< 6.5 g/dL<br>*< 1.01 mmol/L*

LABORATORY					
PARAMETER		GRADE 1 MILD	GRADE 2<br>MODERATE	GRADE 3<br>SEVERE	GRADE 4<br>POTENTIALLY<br>LIFE-THREATENING
	**Adult and Paediatric ≥ 57 days** (HIV NEGATIVE ONLY)	10.0 – 10.9 g/dL<br>*1.55 – 1.69 mmol/L* OR<br>Any decrease<br>2.5 – 3.4 g/dL<br>*0.39 – 0.53 mmol/L*	9.0 – 9.9 g/dL<br>*1.40 – 1.54 mmol/L* OR<br>Any decrease<br>3.5 – 4.4 g/dL<br>*0.54 – 0.68 mmol/L*	7.0 – 8.9 g/dL<br>*1.09 – 1.39 mmol/L* OR<br>Any decrease<br>≥ 4.5 g/dL<br>*≥ 0.69 mmol/L*	< 7.0 g/dL<br>*< 1.09 mmol/L*
International Normalized Ratio of prothrombin time (INR)		1.1 – 1.5 x ULN	1.6 – 2.0 x ULN	2.1 – 3.0 x ULN	> 3.0 x ULN
Methemoglobin		5.0 – 10.0%	10.1 – 15.0%	15.1 – 20.0%	> 20.0%
Prothrombin Time (PT)		1.1 – 1.25 x ULN	1.26 – 1.50 x ULN	1.51 – 3.00 x ULN	> 3.00 x ULN
Partial Thromboplastin Time (PTT)		1.1 – 1.66 x ULN	1.67 – 2.33 x ULN	2.34 – 3.00 x ULN	> 3.00 x ULN
Platelets, decreased		100,000 – 124,999/mm^3<br>*100.000 x 10^9 – 124.999 x 10^9/L*	50,000 – 99,999/mm^3<br>*50.000 x 10^9 – 99.999 x 10^9/L*	25,000 – 49,999/mm^3<br>*25.000 x 10^9 – 49.999 x 10^9/L*	< 25,000/mm^3<br>*< 25.000 x 10^9/L*
WBC, decreased		2,000 – 2,500/mm^3<br>*2.000 x 10^9 – 2.500 x 10^9/L*	1,500 – 1,999/mm^3<br>*1.500 x 10^9 – 1.999 x 10^9/L*	1,000 – 1,499/mm^3<br>*1.000 x 10^9 – 1.499 x 10^9/L*	< 1,000/mm^3<br>*< 1.000 x 10^9/L*
**CHEMISTRIES** ***Standard International Units are listed in italics***					
Acidosis		NA	pH < normal, but ≥ 7.3	pH < 7.3 without life threatening consequences	pH < 7.3 with life threatening consequences
Albumin, serum, low		3.0 g/dL – < LLN<br>*30 g/L – < LLN*	2.0 – 2.9 g/dL<br>*20 – 29 g/L*	< 2.0 g/dL<br>*< 20 g/L*	NA
Alkaline Phosphatase		1.25 – 2.5 x ULN†	2.6 – 5.0 x ULN†	5.1 – 10.0 x ULN†	> 10.0 x ULN†

LABORATORY				
**PARAMETER**	**GRADE 1 MILD**	**GRADE 2 MODERATE**	**GRADE 3 SEVERE**	**GRADE 4 POTENTIALLY LIFE-THREATENING**
Alkalosis	NA	pH > normal, but ≤ 7.5	pH > 7.5 without life threatening consequences	pH > 7.5 with life threatening consequences
ALT (SGPT)	1.25 – 2.5 x ULN	2.6 – 5.0 x ULN	5.1 – 10.0 x ULN	> 10.0 x ULN
AST (SGOT)	1.25 – 2.5 x ULN	2.6 – 5.0 x ULN	5.1 – 10.0 x ULN	> 10.0 x ULN
Bicarbonate, serum, low	16.0 mEq/L – < LLN<br>*16.0 mmol/L – < LLN*	11.0 – 15.9 mEq/L<br>*11.0 – 15.9 mmol/L*	8.0 – 10.9 mEq/L<br>*8.0 – 10.9 mmol/L*	< 8.0 mEq/L<br>*< 8.0 mmol/L*
Bilirubin (Total)				
**Adult and Paediatric >14 days**	1.1 – 1.5 x ULN	1.6 – 2.5 x ULN	2.6 – 5.0 x ULN	> 5.0 x ULN
Calcium, serum, high (corrected for albumin)				
**Adult and Paediatric ≥ 7 days**	10.6 – 11.5 mg/dL<br>*2.65 – 2.88 mmol/L*	11.6 – 12.5 mg/dL<br>*2.89 – 3.13 mmol/L*	12.6 – 13.5 mg/dL<br>*3.14 – 3.38 mmol/L*	> 13.5 mg/dL<br>*> 3.38 mmol/L*
Calcium, serum, low (corrected for albumin)				
**Adult and Paediatric ≥ 7 days**	7.8 – 8.4 mg/dL<br>*1.95 – 2.10 mmol/L*	7.0 – 7.7 mg/dL<br>*1.75 – 1.94 mmol/L*	6.1 – 6.9 mg/dL<br>*1.53 – 1.74 mmol/L*	< 6.1 mg/dL<br>*< 1.53 mmol/L*
Cardiac troponin I (cTnI)	NA	NA	NA	Levels consistent with myocardial infarction or unstable angina as defined by the manufacturer
Cardiac troponin T (cTnT)	NA	NA	NA	≥ 0.20 ng/mL OR Levels consistent with myocardial infarction or unstable angina as defined by the manufacturer
Cholesterol (fasting)				

LABORATORY				
**PARAMETER**	**GRADE 1 MILD**	**GRADE 2 MODERATE**	**GRADE 3 SEVERE**	**GRADE 4 POTENTIALLY LIFE-THREATENING**
**Adult ≥ 18 years**	200 – 239 mg/dL<br>*5.18 – 6.19 mmol/L*	240 – 300 mg/dL<br>*6.20 – 7.77 mmol/L*	> 300 mg/dL<br>*>7.77 mmol/L*	NA
Creatinine Kinase	3.0 – 5.9 x ULN†	6.0 – 9.9 x ULN†	10.0 – 19.9 x ULN†	≥ 20.0 x ULN†
Creatinine	1.1 – 1.3 x ULN†	1.4 – 1.8 x ULN†	1.9 – 3.4 x ULN†	≥ 3.5 x ULN†
Glucose, serum, high				
Non fasting	116 – 160 mg/dL<br>*6.44 – 8.88 mmol/L*	161 – 250 mg/dL<br>*8.89 – 13.88 mmol/L*	251 – 500 mg/dL<br>*13.89 – 27.75 mmol/L*	> 500 mg/dL<br>*> 27.75 mmol/L*
Fasting	110 – 125 mg/dL<br>*6.11 – 6.94 mmol/L*	126 – 250 mg/dL<br>*6.95 – 13.88 mmol/L*	251 – 500 mg/dL<br>*13.89 – 27.75 mmol/L*	> 500 mg/dL<br>*> 27.75 mmol/L*
Glucose, serum, low				
**Adult and Paediatric ≥ 1 month**	55 – 64 mg/dL<br>*3.05 – 3.55 mmol/L*	40 – 54 mg/dL<br>*2.22 – 3.06 mmol/L*	30 – 39 mg/dL<br>*1.67 – 2.23 mmol/L*	< 30 mg/dL<br>*< 1.67 mmol/L*
Lactate	< 2.0 x ULN without acidosis	≥ 2.0 x ULN without acidosis	Increased lactate with pH < 7.3 without life threatening consequences	Increased lactate with pH < 7.3 with life threatening consequences
LDL cholesterol (fasting)				
**Adult ≥ 18 years**	130 – 159 mg/dL<br>*3.37 – 4.12 mmol/L*	160 – 190 mg/dL<br>*4.13 – 4.90 mmol/L*	≥ 190 mg/dL<br>*≥ 4.91 mmol/L*	NA
Lipase	1.1 – 1.5 x ULN	1.6 – 3.0 x ULN	3.1 – 5.0 x ULN	> 5.0 x ULN
Magnesium, serum, low	1.2 – 1.4 mEq/L<br>*0.60 – 0.70 mmol/L*	0.9 – 1.1 mEq/L<br>*0.45 – 0.59 mmol/L*	0.6 – 0.8 mEq/L<br>*0.30 – 0.44 mmol/L*	< 0.60 mEq/L<br>*< 0.30 mmol/L*
Pancreatic amylase	1.1 – 1.5 x ULN	1.6 – 2.0 x ULN	2.1 – 5.0 x ULN	> 5.0 x ULN
Phosphate, serum, low				

LABORATORY

**PARAMETER**	**GRADE 1 MILD**	**GRADE 2 MODERATE**	**GRADE 3 SEVERE**	**GRADE 4 POTENTIALLY LIFE-THREATENING**
**Adult and Paediatric > 14 years**	2.5 mg/dL – < LLN<br>*0.81 mmol/L – < LLN*	2.0 – 2.4 mg/dL<br>*0.65 – 0.80 mmol/L*	1.0 – 1.9 mg/dL<br>*0.32 – 0.64 mmol/L*	< 1.00 mg/dL<br>*< 0.32 mmol/L*
Potassium, serum, high	5.6 – 6.0 mEq/L<br>*5.6 – 6.0 mmol/L*	6.1 – 6.5 mEq/L<br>*6.1 – 6.5 mmol/L*	6.6 – 7.0 mEq/L<br>*6.6 – 7.0 mmol/L*	> 7.0 mEq/L<br>*> 7.0 mmol/L*
Potassium, serum, low	3.0 – 3.4 mEq/L<br>*3.0 – 3.4 mmol/L*	2.5 – 2.9 mEq/L<br>*2.5 – 2.9 mmol/L*	2.0 – 2.4 mEq/L<br>*2.0 – 2.4 mmol/L*	< 2.0 mEq/L<br>*< 2.0 mmol/L*
Sodium, serum, high	146 – 150 mEq/L<br>*146 – 150 mmol/L*	151 – 154 mEq/L<br>*151 – 154 mmol/L*	155 – 159 mEq/L<br>*155 – 159 mmol/L*	≥ 160 mEq/L<br>*≥ 160 mmol/L*
Sodium, serum, low	130 – 135 mEq/L<br>*130 – 135 mmol/L*	125 – 129 mEq/L<br>*125 – 129 mmol/L*	121 – 124 mEq/L<br>*121 – 124 mmol/L*	≤ 120 mEq/L<br>*≤ 120 mmol/L*
Triglycerides (fasting)	NA	500 – 750 mg/dL<br>*5.65 – 8.48 mmol/L*	751 – 1,200 mg/dL<br>*8.49 – 13.56 mmol/L*	> 1,200 mg/dL<br>*> 13.56 mmol/L*
Uric acid	7.5 – 10.0 mg/dL<br>*0.45 – 0.59 mmol/L*	10.1 – 12.0 mg/dL<br>*0.60 – 0.71 mmol/L*	12.1 – 15.0 mg/dL<br>*0.72 – 0.89 mmol/L*	> 15.0 mg/dL<br>*> 0.89 mmol/L*
**URINALYSIS** *Standard International Units are listed in italics*				
Hematuria (microscopic)	6 – 10 RBC/HPF	> 10 RBC/HPF	Gross, with or without clots OR with RBC casts	Transfusion indicated
Proteinuria, random collection	1 +	2 – 3 +	4 +	NA
Proteinuria, 24 hour collection				
**Adult and Paediatric ≥ 10 years**	200 – 999 mg/24 h<br>*0.200 – 0.999 g/d*	1,000 – 1,999 mg/24 h<br>*1.000 – 1.999 g/d*	2,000 – 3,500 mg/24 h<br>*2.000 – 3.500 g/d*	> 3,500 mg/24 h<br>*> 3.500 g/d*

* Values are for term infants.
† Use age and sex appropriate values (e.g., bilirubin), including preterm infants.

20 Appendix 3 Liverpool Causality Assessment Tool for Adverse Drug Reactions

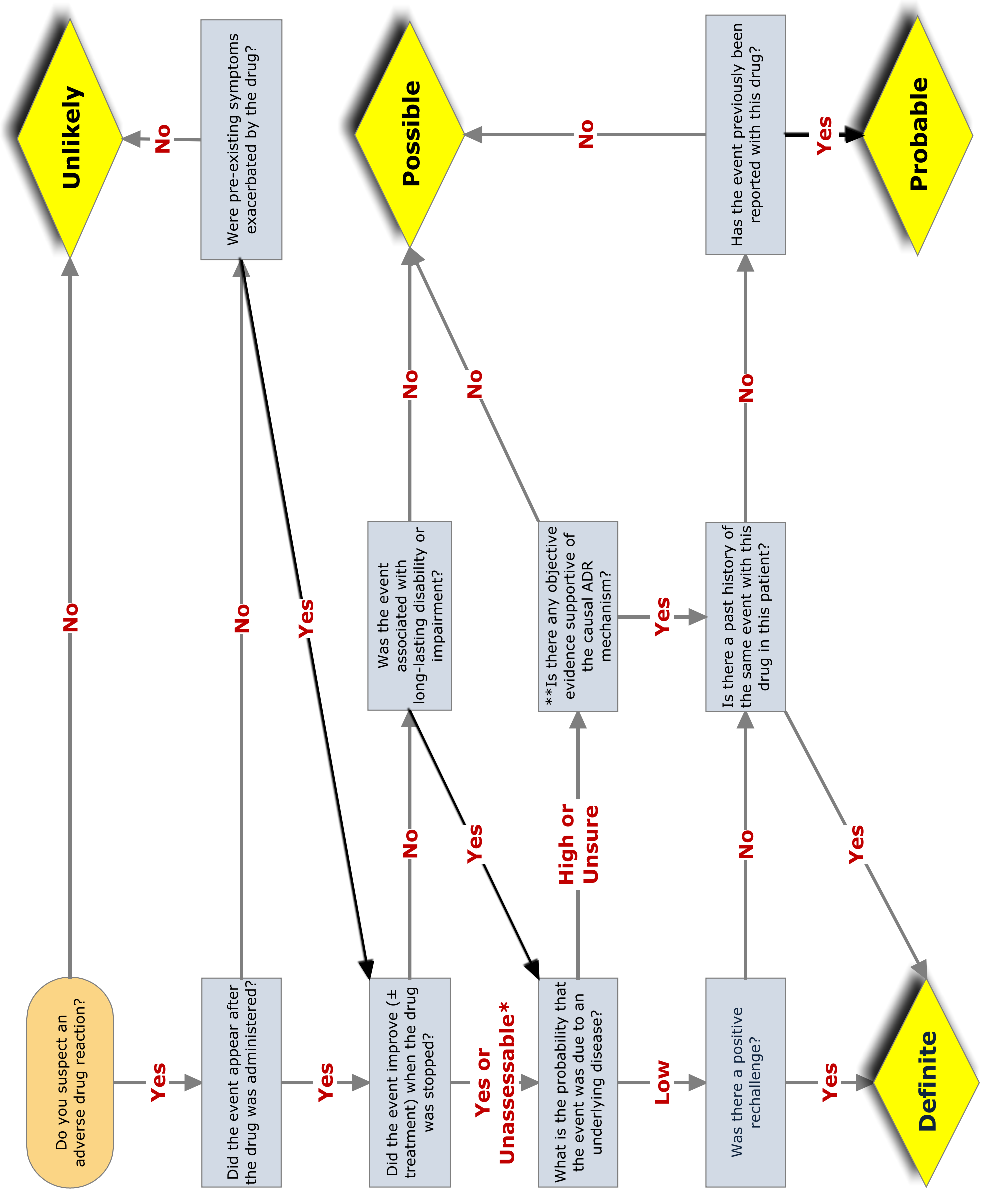

21 Appendix 4 Community Consultation

Potential Concern	Response	Safeguard
Why is the study targeting pregnant women? These are a very vulnerable group of individuals.	We recognise that pregnant women with a new HIV diagnosis are indeed vulnerable, socially, emotionally, mentally and importantly also from the point of view of having a higher than usual risk of transmitting HIV to their infant. Because mother to child transmission is potentially preventable, and in women presenting late in pregnancy the window of opportunity to provide effective treatment is very small indeed, we believe that this group is especially important to study, since dolutegravir may well become the drug of first choice in this scenario.	Within the existing maternity service, trained nurses and counsellors are available to help with psychosocial issues. We accept a new diagnosis will be traumatic, and we will not be recruiting mothers who have evidence of mental illness or excessive stress, or decline to give consent.<br><br>We will be submitting this proposal to ethics committees in the UK and in the countries where the study is to be undertaken
Is the drug really safe in pregnancy? What if toxicity or side effects develop?	Most drugs that are licensed for use lack safety data in pregnancy, because these studies are rarely performed. The almost universal scenario is for a new drug to become licensed (with only reproductive toxicity and pregnancy date derived from animal studies guiding FDA pregnancy risk category). That drug then becomes widely deployed including to pregnant women. For a few drugs (including antiretrovirals) specific pregnancy registers are established in certain countries in order to track drug safety in mothers and their newborn infants.<br><br>There is an ethical argument that states that if a drug is going to be widely deployed (eg. a first or second line antiretroviral agent), and inevitably administered to large numbers of pregnant women, it is better to know in advance of any registry, that the dosing is optimised, and ideally a small number of women and their babies are closely followed in order to ascertain safety and efficacy. The problem with registries is that although they may contain large numbers of people, safety	We will conduct close virological, haematological and biochemical monitoring for toxicity. These will include existing toxicities which are already recognised, plus unanticipated toxicities in the mother and the infant.<br><br>We will also establish a data safety monitoring group (IDSMB). This will be an independent international committee made up of experts in drug safety and HIV pharmacology, as well as community representatives. The IDSMB is scheduled to meet prior to the enrollment of the first patient, during the interim analysis (after the first 10 patients have delivered) and upon completion of the study. At each point the IDSMB will review laboratory and clinical data relating to mother and child. The committee will have an independent Chair, and have the power to stop the study at any stage if it feels that the safety of patients

	monitoring data (eg blood tests) tend to be sparse.	is being compromised. Equally, if during the course of the study (and before all patients are enrolled) there is clear evidence of statistical superiority, or likely futility of the study, the committee is able to recommend that the study is terminated prematurely.
You expect this drug to be better than the current standard of care in Uganda. What if that doesn't hold true in pregnancy?	We will be tracking HIV viral load closely during the course of the study up to the point of delivery. We will also be confirming (with molecular tests) infection in the newborn infant.	IDSMB as above
The researchers propose to use a combination of ARVs which differs from the currently used national policy (based on WHO B+ guidance). Whilst the data obtained may be useful, is this safe for the individual mother?	Studies investigating the safety and efficacy of dolutegravir in non-pregnant patients have shown it to have few side effects, and to bring about a rapid reduction in HIV viral load, which will be a great advantage to women who are diagnosed with HIV towards the end of their pregnancy. We specifically aim to recruit women who, if given the current standard regimen, would remain at substantial risk of transmitting the HIV to their infant. We therefore anticipate a beneficial effect for the individual.<br><br>From what is known about how the body handles drugs during pregnancy, there is no reason to anticipate that the effects of the drug will differ during pregnancy.	Community advisory group (Friends Council), Uganda<br><br>Ongoing review by IDSMB<br><br>Balanced against any potential risk to a group of closely monitored volunteers, is an ethical imperative to make sure that treatment of pregnant women is evidence based, rather than occurring on a widespread basis with no clinical or virological monitoring
What relevance is this drug to Africa? Will such a new drug be affordable? Or are people in developing countries being 'used' in studies which only people in Europe and America will benefit from?	The manufacturer of dolutegravir (ViiV Healthcare) is working with generic manufacturers to prioritise generic versions of dolutegravir (target -within eighteen months of FDA licensing on 12th August 2013). ViiV are already in discussion with key generic manufacturers. The World Health Organisation has been discussing the potential role of dolutegravir as either a second line or even a first line regimen for low and middle income countries. The Clinton Foundation has estimated that with scale up of manufacturing costs, the potential pricing of a generic version of dolutegravir could match the cheapest component of the current first line regimen in Uganda.	There is strong evidence that dolutegravir will be affordable, available, and widely utilised in Sub Saharan Africa.

What are the side effects?	All ARVs have some side effects. For dolutegravir, these are most commonly nausea or gastro-intestinal disturbance, headaches or a feeling of weakness and fatigue.	Patients enrolled in the study will be monitored closely for any adverse effects which are either noticeable clinically, or detectable on blood tests. As detailed above, the DSMB will closely monitor the individuals enrolled in the study to ensure safety
How safe is this drug for my baby ?	There are no safety data in newborn infants (and this study will contribute important knowledge). Dolutegravir is classified as an FDA Category B drug (Animal reproduction studies have failed to demonstrate a risk to the fetus and there are no adequate and well-controlled studies in pregnant women).	If introduced into first/second line treatment, many infants will be exposed in Africa without any close monitoring or evaluation. Our safety assessments are clinical (neonatology examination, infant safety evaluation questionnaire) , pharmacological and virological.
Why is dolutegravir being continued for 2 weeks after delivery (when the mother is breastfeeding) ?	It is essential to compare drug exposure in the same person during and after pregnancy- otherwise changes in drug exposure could be related to diet, ethnicity and other factors. Late presentation renders a mother unlikely to be undetectable at the time of delivery, and many infants could also be receiving an oral dose of HIV during breastfeeding, so effective ‘post exposure prophylaxis’ to the infant may carry potential benefits as well as risks.	Close monitoring of infant safety (see above), and virological studies of breast milk, and PK studies in maternal and infant plasma as well as breastmilk.
Who is funding this study, and what are their roles?	This study is being funded by ViiV Healthcare, who will also be providing drug. The study design, execution and analysis will be carried out by a team of independent researchers based at the University of Liverpool, Infectious Diseases Institute based at Makerere University and the antenatal clinic at SRH.<br><br>The protocol will be discussed with ViiV, who will be asked for input, and also to provide any unpublished data on safety in pregnancy. The responsibility for the final version of the protocol rests with the Principal Investigator.	Transparency of funding will be ensured. All study personnel and IDSMB members will be asked for a Declaration of Interests, to be individually signed and dated.

22 Appendix 5:

EDINBURGH POSTNATAL DEPRESSION SCALE (EPDS)

NAME: ____

Baby's Age: ______________________

As you have recently had a baby, we would like to know how you are feeling. Please UNDERLINE which comes closest to how you have felt IN THE PAST 7 DAYS, not just how you feel today.

Here is an example, already completed.
I have felt happy:

Yes, all the time.
Yes, most of the time.
No, not very often.
No, not at all.

This would mean, "I have felt happy most of the time" during the past week. Please complete the other questions in the same way.

In the Past 7 Days:

1. I have been able to laugh and see the funny side of things as much as I always could.

   0 – As much as I always could
   1 – Not quite so much now.
   2 – Definitely not so much now
   3 – Not at all

2. I have looked forward with enjoyment to things.

   0 – As much as I ever did
   1 – Rather less than I used to
   2 – Definitely less than I used to
   3– Hardly at all

3. I have blamed myself unnecessarily when things went wrong.
   3 – Yes, most of the time.
   2 – Yes, some of the time
   1 – Not very often
   0 – No, never

4. I have been anxious or worried for no good reasons.

   0 – No, not at all.
   1 - Hardly, ever
   2 – Yes, sometimes
   3 - Yes, very often

5. I have felt scared or panicky for no very good reason.

   3– Yes, quite a lot
   2 – Yes, sometimes
   1 – No, not much
   0 – No, not at all

6. Things have been getting on top of me.

   3– Yes, most of the time I haven't been able to cope at all
   2 - Yes, sometimes I haven't been coping as well as usual

1 – No, most of the time I have coped quite will
0 – No, I have been coping as well as ever

7. I have been so unhappy that I have had difficulty sleeping

3– Yes, most of the time
2 – Yes, sometimes
1 – Not very often
0 – No, not at all

8. I have felt sad or miserable

3-Yes, most of the time
2- Yes, quite often
1- Not very often
0- No, not at all

9. I have been so unhappy that I have been crying
3-Yes, most of the time
2- Yes, quite often
1 -Only occasionally
0 – No, not at all

10. The thought of harming myself has occurred to me.
3-Yes, quite often
2-Sometimes
1-Hardly ever
0-Never

Screening Tool for PPD

Edinburgh Postnatal Depression Scale (EPDS) [Cox, Holden & Sagovsky 1987]

The EPDS is a self-rated questionnaire that has been used in Europe and Australia for over 10 years to screen women for PPD. It asks women to rate how they have been feeling in the last 7 days and consists of 10 short statements of common depressive symptoms with 4 choices per statement. Each statement is rated on a scale of 0 – 3 with possible total scores ranging from 0 – 30.

To administer the test you give the woman a pen and the questionnaire and ask her to answer the questions in relation to the past 7 days. The questionnaire should only take a few minutes to complete.

Scoring the questionnaire only take a couple of minutes with practice.
Questions 3,5,6,7,8,9 and 10 are scored: statement 1 = 3 points, statement 2 = 2 points, statement 3 = 1 point and statement 4 = 0 points.

A cut-off score of 12.5 has been shown to detect major depression and a woman who meets this threshold can be further assessed. Asking a woman to complete such a questionnaire not only makes her stop and think about how she has been feeling but also indicates a willingness on the part of the person giving the questionnaire to listen to how she is feeling.
